# Comprehensive analysis of microorganisms accompanying human archaeological remains

**DOI:** 10.1093/gigascience/gix044

**Published:** 2017-06-13

**Authors:** Anna Philips, Ireneusz Stolarek, Bogna Kuczkowska, Anna Juras, Luiza Handschuh, Janusz Piontek, Piotr Kozlowski, Marek Figlerowicz

**Affiliations:** 1 European Center for Bioinformatics and Genomics, Institute of Bioorganic Chemistry, Polish Academy of Sciences, Poznan, 61-704, Poland; 2 Department of Human Evolutionary Biology, Institute of Anthropology, Faculty of Biology, Adam Mickiewicz University in Poznan, Poznan, 61-614, Poland; 3 Department of Hematology and Bone Marrow Transplantation, University of Medical Sciences, Poznan, 60-569, Poland; 4 Institute of Technology and Chemical Engineering, Poznan University of Technology, Poznan, 60-965, Poland; 5 Institute of Computing Science, Poznan University of Technology, Poznan, 60-965, Poland

**Keywords:** microbiome, ancient DNA, NGS, metagenomics

## Abstract

Metagenome analysis has become a common source of information about microbial communities that occupy a wide range of niches, including archaeological specimens. It has been shown that the vast majority of DNA extracted from ancient samples come from bacteria (presumably modern contaminants). However, characterization of microbial DNA accompanying human remains has never been done systematically for a wide range of different samples. We used metagenomic approaches to perform comparative analyses of microorganism communities present in 161 archaeological human remains. DNA samples were isolated from the teeth of human skeletons dated from 100 AD to 1200 AD. The skeletons were collected from 7 archaeological sites in Central Europe and stored under different conditions. The majority of identified microbes were ubiquitous environmental bacteria that most likely contaminated the host remains not long ago. We observed that the composition of microbial communities was sample-specific and not correlated with its temporal or geographical origin. Additionally, traces of bacteria and archaea typical for human oral/gut flora, as well as potential pathogens, were identified in two-thirds of the samples. The genetic material of human-related species, in contrast to the environmental species that accounted for the majority of identified bacteria, displayed DNA damage patterns comparable with endogenous human ancient DNA, which suggested that these microbes might have accompanied the individual before death. Our study showed that the microbiome observed in an individual sample is not reliant on the method or duration of sample storage. Moreover, shallow sequencing of DNA extracted from ancient specimens and subsequent bioinformatics analysis allowed both the identification of ancient microbial species, including potential pathogens, and their differentiation from contemporary species that colonized human remains more recently.

## Background

During the last 2 decades, a number of methods that permit isolation and sequencing of ancient DNA (aDNA) extracted from archaeological specimens have been elaborated. As a result, several complete genome sequences of long-dead organisms have been determined [1–5]. Typically, aDNA is sampled from teeth or bones as these are the densest tissues in vertebrates, which supports the preservation of aDNA in crystal aggregates [6, 7]. Ancient remains are usually deposited in soils for decades, so DNA extracted is a mix of host DNA fragments and DNA from different organisms inhabiting the environment. To avoid the contamination that is usually present on bone/teeth surfaces (e.g., modern human, bacterial, fungal, or plant DNA), aDNA is sampled from interior parts, where the amount of aDNA is the highest. Despite applying rigorous DNA extraction protocols, the endogenous aDNA usually constitutes much less than 5% of the total extracted DNA, e.g., 1–5% for a Neanderthal [2] and 4% for a Mal’ta boy (24 000-year-old human) [8]. Of the remaining DNA, typically >95% is DNA of different microorganisms that have colonized the remains and have been acquired from the environment. When younger remains are considered (100–200 years old), the amount of endogenous aDNA is not much higher [9]; however, it is possible to obtain a sample containing even up to 70% of endogenous aDNA [4, 10]. This is because the preservation of DNA depends on many environmental factors [11, 12]. For example, cold temperatures [13, 14], microclimate of caves where remains have been buried [11], and swampy sediments [12] are known to enhance DNA stability. Moreover, it has been shown that the vast majority of DNA isolated from archaeological human remains belongs to bacteria that have colonized the remains [15, 16]. Bacteria amplify the porosity of bone and teeth [17, 18], making them more accessible to water, which may lead to so-called endogenous aDNA leaching [19] and replacement by exogenous DNA.

Some target enrichment procedures have been proposed to increase the amount of endogenous aDNA [20–24], and among them is the 2-step digestion method [14, 25, 26]. Interestingly, Orlando and colleagues showed that 2-step digestion does not influence the composition of bacterial communities (e.g., is the same in aDNA samples obtained after the first and second digestion runs) [9]. This observation suggests that niches exist deep within the bones and teeth. The environmental bacteria may reach these niches and preserve there.

Metagenome analysis has become a common source of information about microbial communities that occupy a wide range of ecosystems. Until today, environmental components [27] as well as flora of different human sites [28], e.g., oral [29, 30], skin [31], or intestinal [32–35], have been well characterized. In our study, we used this approach to analyze microorganisms that accompany archaeological human remains, which until now have not been exhaustively compared. Prior findings are limited to the rough identification of environmental bacteria [16] or concern a singular species, usually pathogenic. In the latter cases, the analyses were mostly undertaken after the identification of visible symptoms of past disease [36–38]. Efforts have also been undertaken to characterize human mummy intestinal [39] and colon [40] microbes, as well as the ancient oral microbiome [41–44]. They showed that aDNA of species that colonized the organism before death may be obtained. However, comprehensive characterization of microbial DNA accompanying human remains has never been done.

The current study was performed to characterize microorganisms associated with human archaeological remains. We used shotgun sequencing of DNA isolated from 161 human teeth collected from 7 archaeological sites dated from 100 AD to 1200 AD and stored under different conditions (e.g., museum or grave). For each individual sample, the microbiome was determined using Metagenomic Phylogenetic Analysis (MetaPhlAn2) based on multiple specific marker sequences derived from the genomes of microorganisms [45, 46]. Within this study, we focused on bacteria and archaea, which are known to constitute the majority of exogenous DNA in human archaeological remains [15, 16]. We checked whether microbial communities associated with specimens from different archaeological sites or of different ages were taxonomically and functionally distinct. We also attempted to identify microbes that may accompany the organism even before death and to distinguish bacteria/archaea that stem from postmortem contamination from those of original flora by studying their DNA damage patterns.

## Data Description

**Figure 1:**
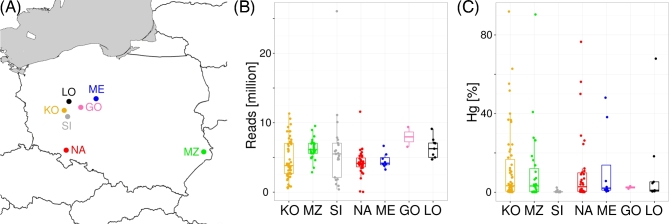
(**A**) The geographical positions of archaeological sites. KO and MZ are from the Roman Age group, and SI, NA, ME, GO, and LO are from the Medieval Group. Samples from ME were collected directly at the archaeological site. (**B**) Number of filtered reads (y-axis) per archaeological site (x-axis). (**C**) Percentage of reads mapped to the human genome (y-axis) per archaeological site (x-axis).

We analyzed 161 human bone samples collected from 7 archaeological sites in Central Europe (Fig. 1A). As shown in Table 1, the samples differed by age (Roman Age group [KO and MZ] or Medieval group [GO, SI, NA, ME, and LO]) and by storage conditions (specimens that were in museum deposits for at least 20 years [long deposit: KO, MZ, SI, NA, and GO], relatively freshly discovered specimens [stored in museum deposit <5 years, short deposit: LO], or samples taken directly from an archaeological site [arch. site: ME]). Carbon isotope dating of the selected samples correlated well with dating based on archaeological analysis (see Supplementary Table S1).

**Table 1:** Characteristics of the samples extracted from ancient human remains

Archaeological site	ID	Sample no.	Sample no. that passed selection	Dating	Date of excavation	Storage conditions	Sample type
Roman Age group							
Kowalewko	KO	58	48	100–300 AD	1990s	Long deposit	Tooth
Masłomęcz	MZ	27	24	200–400 AD	1970–1990	Long deposit	Tooth
Medieval group							
Sowinki	SI	21	19	1000–1100 AD	1980s	Long deposit	Tooth
Niemcza	NA	36	31	900–1000 AD	1960s	Long deposit	Tooth
Markowice	ME	8	8	1000–1200 AD	2014	Arch. site	Tooth
Gniezno	GO	2	2	1000–1200 AD	1980s	Long deposit	Tooth
Łęgowo	LO	9	8	1000–1200 AD	2013–2015	Short deposit	Tooth

Ancient DNA was always extracted from the roots of teeth. We drilled those parts of the roots that include both dentine and cementum. In all cases, enamel and cementum were preserved. Subsequently, all DNA samples were subjected to shallow next-generation sequencing (NGS) with the usage of an Illumina single-end standard protocol (including blunt-end DNA repair) and 75 bp sequencing run. Altogether, 846.5 million reads were obtained. On average, 98.6% of reads passed trimming and quality filtration. After filtration, for 161 samples, the average number of reads per sample was 5 143 975 (median = 4 730 243; range = 34 857–26 055 295). In further analysis, we removed 8 samples that did not meet the arbitrary criterion of minimal raw reads number (<1 million). The average numbers of reads differ between archaeological sites (Kruskal-Wallis: *P* = 0.0166), but not between types of sample storage (Wilcoxon: *P* = 0.2685) or age (Wilcoxon: *P* = 0.5607) (Fig. 1B). Detailed information on each sample is summarized in Supplementary Table S1.

All reads were mapped to the reference human genome, and the percentage of human reads was determined for each sample. As shown in Fig. 1C, the fraction of human aDNA ranged from 0.01% to 91.9%; however, in most cases (100 samples), it was less than 5%. Nine samples had more than 50% human aDNA content. Differences in the amount of human aDNA content were observed for different archeological sites (Kruskal-Wallis: *P* = 6.124e-05), but not for freshly recovered and stored in museum samples (Wilcoxon: *P* = 0.3160). Marginal statistical significance was observed between older (KO, MZ) and younger (SI, NA, ME, GO, LO) samples (Wilcoxon: *P* = 0.0467), with a higher share of endogenous human DNA in older samples (average = 11.7% and 7.8%, median = 3.2% and 0.75%, for older and younger samples, respectively).

## Analyses

### Microbiomes of human archaeological remains

To characterize the microbiomes of analyzed archaeological samples, we used MetaPhlAn2. The program identifies bacteria/archaea, viruses/viroids, and unicellular eukaryotes using homology-based classification of NGS reads by alignment with predefined taxa-specific marker sequences [45]. The number of reads mapped to MetaPhlAn2 markers ranged from 708 (sample KO_014) to 95 950 (sample KO_006). Two samples with <1000 reads mapped to the marker sequences were removed from further analyses as the marker coverage is crucial for proper microorganism detection [46].

**Figure 2:**
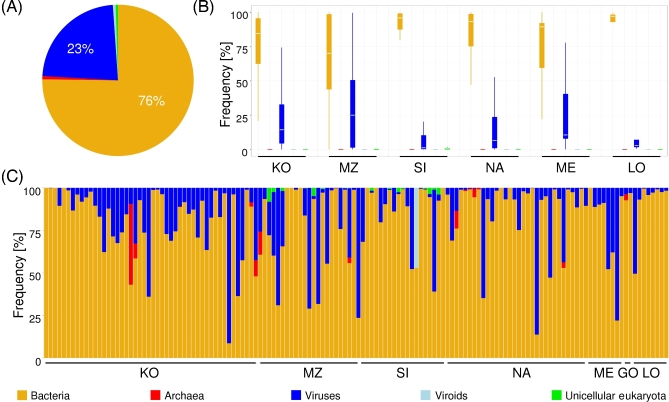
Microorganism kingdoms detected in analyzed archaeological samples. (**A**) Pie chart representing overall frequency of microorganism kingdoms in archaeological samples. (**B**) Box and whisker plot representing the distribution of frequencies of particular microorganism kingdoms in archaeological sites (GO not shown as it includes only 2 samples). (**C**) Stacked barplot indicating the frequency of microorganism kingdoms in a particular sample. Each bar represents an individual sample. Samples are ordered by the archeological sites. The color legend for all plots is shown at the bottom.

For the remaining 151 samples, our analyses (Fig. 2A) showed that the majority of reads mapped to bacterial or archaeal markers (76.4%) and 23.4% to virus/viroid markers. The remaining 0.2% constituted eukaryotes (present in 13 samples; 0.6–8.2%), which were subsequently identified as fungi, protists, or protozoa. The contributions of the particular types of microorganisms differed substantially between individual samples (in 12 samples, we found only bacteria; in sample KO_28, only viruses were identified) (Fig. 2C). However, these differences did not correlate with archaeological site (multivariate analysis of variance [MANOVA]: *P* = 0.0532) (Fig. 2B), sample age (MANOVA: *P* = 0.2054), or storage conditions (MANOVA: *P* = 0.7672).

The virus fraction varied from 0.1% to 99% between samples. Analysis of virus taxa showed that most of them were associated with plants; hence, we reasoned that they may have been acquired from the environment and were possibly indigenous flora. The most abundant viruses, *Dasheen mosaic virus* (58% of all identified viruses/viroids) and *Vicia cryptic virus* (26.7%), are both known to infect plants. Subsequently, 5 viruses and 1 viroid constituted less than 2.5% each of all identified viruses/viroids, and also all were found to be associated with plant genera (*Ageratum, Sauropus, Cichorium*, or *Malvastrum*). The remaining viruses were of low abundance (<1%) and were usually present in no more than a single sample. It is also noteworthy that we identified within our samples *Propionibacterium* phage—a double-stranded DNA (dsDNA) virus that is associated with oral microbiome [47, 48]. Detailed information on the microorganism composition in individual samples is available in Supplementary Table S2.

### Characterization of bacteria and archaea in human archaeological remains

In the next step, we focused on the prokaryotic component of the analyzed microbiomes. We decided to exclude from this analysis samples with a very high fraction of viruses/viroids. As a result, 11 samples with fewer than 1000 reads mapping exclusively to bacterial/archaeal MetaPhlaAn2 marker sequences were removed as they did not ensure a reliable microbiome profiling.

**Figure 3:**
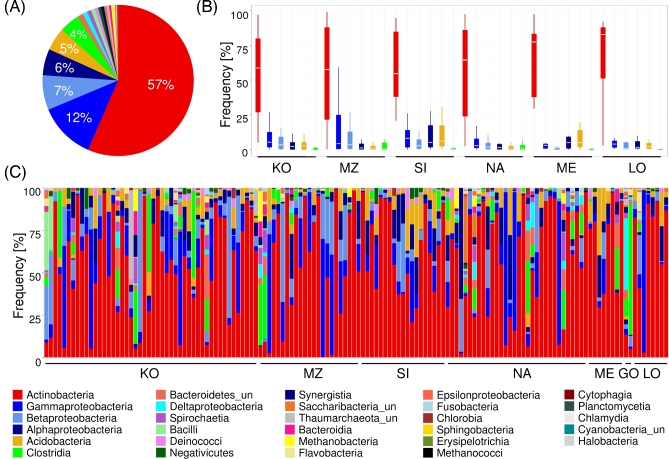
Bacterial and archaeal classes detected in analyzed archaeological samples. (**A**) Pie chart representing overall frequency of bacterial and archaeal classes in archaeological samples. (**B**) Box and whisker plot representing the distribution of frequencies of the 6 most abundant bacterial classes (present in at least 1%) of archaeological sites (GO not shown as it includes only 2 samples). (**C**) Stacked barplot indicating the frequency of bacterial and archaeal classes in a particular sample. Each stacked bar represents an individual sample. Samples are ordered by the archeological sites. The color legend for all plots is shown at the bottom.

Altogether, 25 bacterial and 4 archaeal classes were identified in exogenous DNA of the analyzed samples, and among them, 6 bacterial classes accounted for >1% of identified bacteria/archaea. The most abundant classes were *Actinobacteria* (average = 57%; range = 0.18–98.9%), 3 classes of *Proteobacteria* (*Alphaproteobacteria* [average = 6%; range = 0–65.5%], *Betaproteobacteria* [average = 7%; range = 0–83.6%], *Gammaproteobacteria* [average = 12%; range = 0–95.4%]), *Acidobacteria* (average = 5%; range = 0–39.7%), and *Clostridia* (average = 4%; range =0–76.8%) (Fig. 3A). Although most of the bacteria belonging to the first 5 classes are typically found in the environment (wide range of soils, waters) [27, 49], some of their taxa were human flora components. For example, *Corynebacterium matruchotii* (*Actinobacteria*) [50] and *Lautropia mirabilis* (*Betaproteobacteria*) [51, 52] represented more than 5% of the DNA in 4 samples: KO_046b, NA_121, NA_123, LO_166 and KO_005, KO_006, KO_046b, LO_166, respectively (Supplementary Table S2). *Clostridia* and *Bacteroidetes* are known to include many species inhabiting the human oral cavity or intestines [29]. Additionally, we found, in individual samples, markers characteristic for human pathogens, e.g., *Pseudoramibacter alactolyticus* (*Clostridia*) in sample MZ_88 [53] and *Bordetella parapertussis (Betaproteobacteria*) [54] in sample SI_084; *Clostridium sordellii* and *Clostridium tetani* (*Clostridia*) [55, 56] were found in 2 samples and 1 sample, respectively. Prokaryotic profiles differed substantially between individual samples (Fig. 3C) but did not differ between specific archaeological sites (MANOVA: *P* = 0.3650) (Fig. 3B), sample ages (MANOVA: *P* = 0.3550), or storage conditions (MANOVA: *P* = 0.4729). Similar high variation between individual samples and lack of specificity to archaeological sites was observed when prokaryotes were divided into groups based on gram +/- type (MANOVA: *P* = 0.4364) or oxygen requirements (aerobic, facultative aerobic, anaerobic, facultative anaerobic; MANOVA: *P* = 0.5726) (see Supplementary Figs S1 and S2).

**Figure 4:**
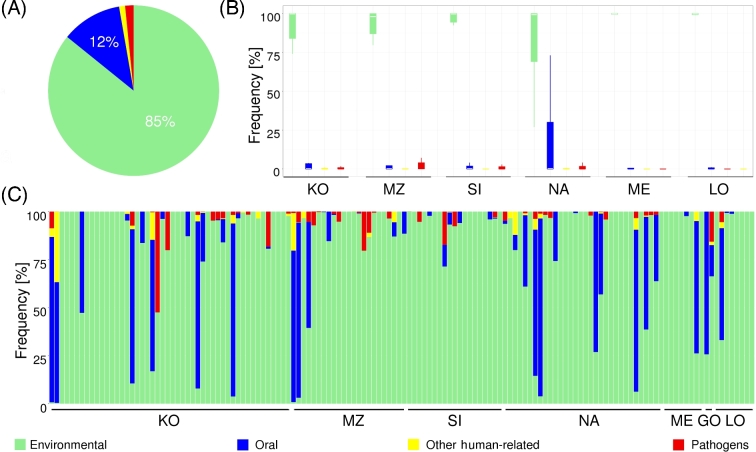
Bacterial and archaeal types (environmental [light green], oral [blue], other [yellow], and pathogenic [red]) detected in analyzed archaeological samples. (**A**) Pie chart representing overall frequency of bacterial and archaeal types in archaeological samples. (**B**) Box and whisker plot representing the distribution of frequencies of bacterial and archaeal types in archaeological sites (GO not shown as it includes only 2 samples). (**C**) Stacked barplot indicating the frequency of bacterial and archaeal types in a particular sample. Each stacked bar represents an individual sample. Samples are ordered by the archeological sites. The color legend for all plots is shown at the bottom.

The identification of singular prokaryotic taxa that are human- rather than environment-related motivated us to determine the fraction of microbes potentially associated with humans. All identified bacteria and archaea were divided on a genus level into 2 groups: environmental and human-related. The latter was further divided into 3 subgroups: oral, potential pathogens, and other (mostly gut). The genus characteristics were inferred based on the features of species identified by MetaPhlAn2. A genus was classified as human-related only if all species of this genus identified in our samples were human-related. The analysis showed that the majority (85.19%) of all bacteria/archaea were environmental (coming from soil and/or water); however, a substantial fraction of the investigated taxa (14.81%) were human-related, including 12.43% of microbes typical for human oral flora, 1.33% of potentially pathogenic bacteria, and 1.05% of other (see Fig. 4A and B). As shown in Fig. 4C, the fraction of human-related genera varied significantly among samples, and some of these genera constituted most of the exogenous DNA. Although the fraction of human-related genera did not differ significantly between archaeological sites (1-way ANOVA: *P* = 0.7480), it was noteworthy that this fraction was highest in NA, the archaeological site dated to the Middle Ages, from which the samples had been stored in a deposit for more than 20 years (see Fig. 4B). Interestingly, there was no relation between prevalence of human-related microbes and the levels of virus/viroid accumulation or the level of endogenous human aDNA (see Supplementary Table S1). The identification of human-related species in ancient remains raised the question of whether some of them accompanied the individual even before death.

Among all samples, the most frequent genera were the soil bacteria *Brevibacterium* (8.5% of all; present in 53 samples >1%; max. 71%) and *Kribbella* (8.4% of all; present in 60 samples >1%; max. 70%). The most abundant oral genera were *Bacteroidetes (*1.6% of all; present in 23 samples >1%; max. 28%), *Desulfobulbus* (1.4% of all; present in 25 samples >1%; max. 44%), and *Eubacterium* (1.4% of all; present in 20 samples >1%; max. 32%). *Methanobrevibacter* (0.8% of all; in 7 samples >1%; max. 34%), typically found in the human digestive system and in the oral cavity, was the most abundant taxon in the other human-related group as only *M. smithii* (human gut flora component) were identified in our samples (Supplementary Table S2). However, it must be pointed out that the genus *Methanobrevibacter* also contains species commonly found in the oral flora, e.g., *M. oralis*, which was not identified within analyzed samples; *Bordetella* was the most abundant taxon classified as a potential human pathogen (*B. pertussis* is known to cause pertussis; 1.2% of all; in 16 samples >1%; max. 60%).

In general, 89% of the analyzed prokaryotes were aerobic or facultative aerobic (Supplementary Fig. S1), and 63% were gram-positive (Supplementary Fig. S2). However, in the human-related group only (Table 2), the percentage of aerobic or facultative aerobic taxa was smaller (24%, 49%, and 54% for oral, pathogen, and other groups, respectively). Additionally, we found that the gram-negative prokaryotes dominated in the oral group (55%) and gram-positive prokaryotes in the other human-related group (70%). This slight dominance of gram-negative taxa in the oral group might be caused by lysozyme presence in an oral cavity that preferentially protects against gram-positive bacteria [57]. We additionally noticed that gram-negative species dominated (68%) in the potential pathogen group. These characteristics seem very useful for preliminary assessment of bacterial populations accompanying human remains.

**Table 2:** The percentage of bacteria/archaea of a given respiratory type (facultative [aerobic/anaerobic] and gram stain type [positive/negative] within environmental and human-related groups [oral, pathogenic, or other])

Group	(Facultative) anaerobic	(Facultative) aerobic	Gram-positive	Gram-negative
Environmental	4%	96%	66%	34%
Oral	76%	24%	45%	55%
Pathogenic	51%	49%	32%	68%
Other human-related	46%	54%	70%	30%

**Figure 5:**
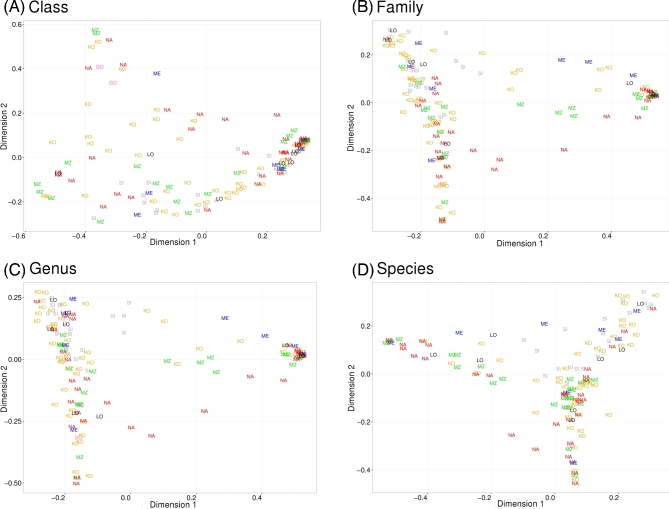
Principal coordinate analysis of microbial compositions at 4 taxonomic levels: (**A**) class, (**B**) family, (**C**) genus, and (**D**) species. Samples from certain archaeological sites are marked in different colors and labeled with an archaeological site ID.

To further investigate whether the prokaryotic profile permits classification of individual samples into specific groups (e.g., samples of similar age or storage conditions or samples from the same archaeological site), we performed Principal Coordinates Analysis (PCoA; Jaccard distance) on 4 taxonomic levels (class, family, genus, and species) (Fig. 5). Samples grouped into 1 big cluster in graphs created on all taxonomic levels. In the PCoA graphs generated on the family, genus, and species levels, there was 1 more significantly smaller cluster visible. Importantly, none of these clusters segregated samples according to the abovementioned features (age, storage, and site). Principal Component Analysis (PCA) (see Supplementary Fig. S3) and the Shannon diversity index (see Supplementary Table S1) again revealed high variation between individual samples at all analyzed taxonomic levels but did not show separation by sample source (species level, 1-way analysis of variance (ANOVA): *P* = 0.5660), sample age (species level, t-test: *P* = 0.5535), or storage type (species level, t-test: *P* = 0.3516). We also tested a hypothesis that the occurrence of some human-related or environmental bacteria might be associated with archeological sites. We performed PCA (Supplementary Fig. S4) and hierarchical clustering (Supplementary Fig. S5) on selected bacterial genera and found out that neither human-related nor environmental microbes segregated samples according to the archeological site, age, or storage type. Finally, we clustered samples based on 10-mer distances between exogenous reads (see the Methods section) and again observed no segregation according to the archeological site, age, or storage type (Supplementary Fig. S6).

In order to confirm that the major source of microbes observed in human archeological samples was the environment, we compared their microbiomes with the microbiomes of humans [58] and soils [27] by PCoA at the genus level (see Supplementary Fig. S7).

### Validation of data obtained using shallow sequencing

**Figure 6:**
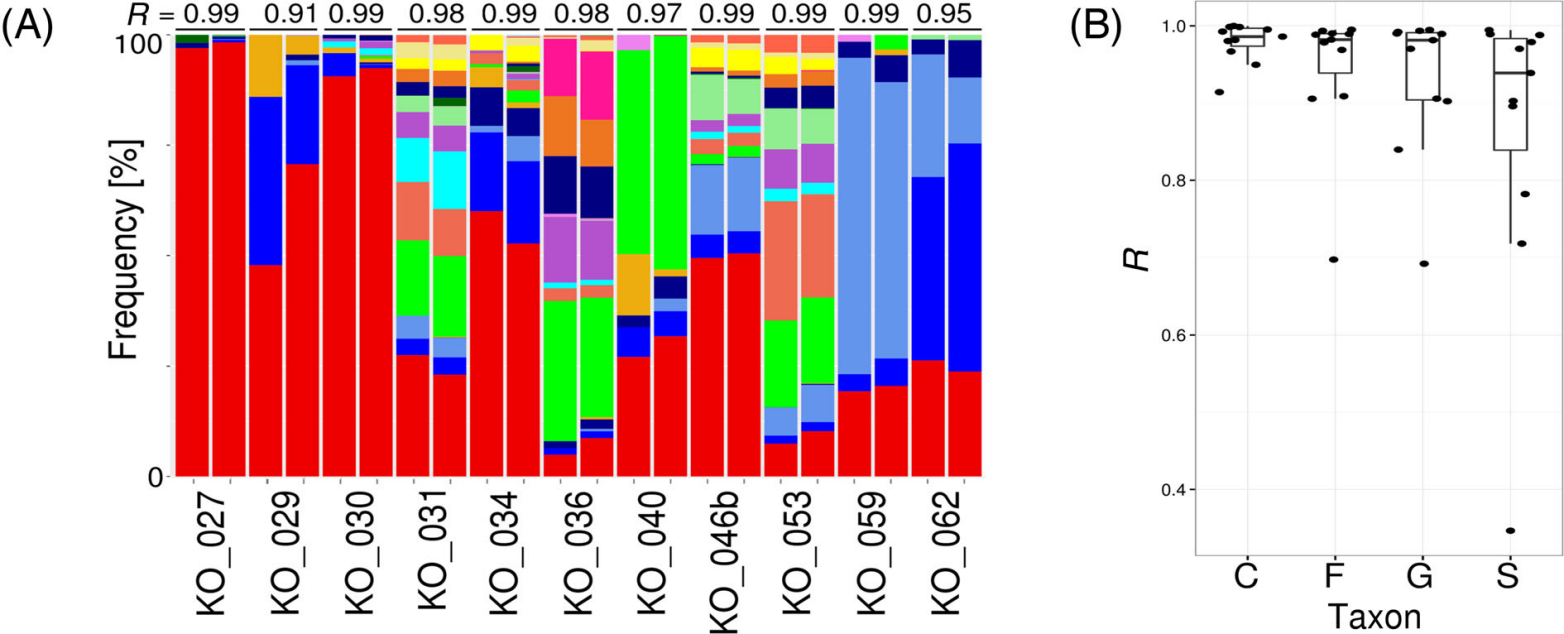
(**A**) Comparison of bacterial and archaeal profiles (stacked barplot) on the class level based on shallow and deep sequencing of the selected 11 samples (sample ID is indicated on the x-axis; the first bar in a pair is shallow, and the second is deep sequencing). The correlation coefficient *R* is placed above each shallow/deep stacked bar pair. The color legend is the same as in Fig. 3. (**B**) Correlation *R* values (y-axis) for shallow and deep sequencing pairs on different taxonomic levels (C: class; F: family; G: genus; S: species).

All results presented above were obtained with the use of datasets generated by relatively shallow sequencing (on average ∼5 million reads per sample). To check the reliability of our results, we determined for the selected samples to what extent the composition of microbiomes (on the class level) is affected by the depth of sequencing. For this analysis, we used 11 representative samples differentiated in terms of (i) filtered read numbers obtained in the shallow sequencing experiment (∼2–8 million), (ii) number of reads mapped to the MetaPhlAn2 markers (∼1000–60 000), and (iii) prokaryotic fraction (∼10–90%). Eight samples were sequenced to the depth of ∼50 million reads and 3 to the depth of ∼100 million reads. Subsequently, we ran a MetaPhlAn2 profiling analysis on deep sequencing datasets. As expected, the total number of filtered reads as well as the number of reads mapping to the MetaPhlAn2 marker sequences increased significantly (about 9-fold); however, the Shannon diversity indexes and microbial compositions remained intact (correlation *R* = 0.91–0.99) (Fig. 6A; Supplementary Table S3). We obtained similar results when we analyzed 3 other taxonomic levels with somehow decreasing *R* with the depth of taxonomic level (average *R* = 0.96, 0.90, 0.88, 0.78 for class, family, genus, and species levels, respectively) (Fig. 6B; Supplementary Fig. S8). It is noteworthy that sample KO_030, second lowest in the number of raw reads, displayed very low correlation (*R* = 0.35) on a species level when results obtained based on shallow sequencing (∼2.6 million reads) and deep sequencing (∼47 million reads) were compared (Supplementary Fig. S8 and Supplementary Table S3). Overall, the correlation coefficient *R* and statistical significance values (*P* < 0.0001 in most cases) (see Supplementary Fig. S8) were still very high and confirmed that the microbial profiles obtained based on the shallow sequencing datasets are reliable and do not change significantly when datasets generated in much deeper sequencing are used to establish them.

### Analysis of age-related aDNA damage patterns

Finally, to verify whether identified human-related prokaryotes are ancient species that colonized the human body before death or are modern contaminants, we analyzed the signatures of age-related DNA damage. Age-related DNA damage was evaluated with the usage of mapDamage2.0 [59], which simulates the posterior distribution of (i) deamination in single-stranded DNA (δ_s_), (ii) deamination in dsDNA (δ_d_), and (iii) the level of DNA fragmentation (λ, represented as: 1/λ-1) [60, 61].

For this analysis, we used sequences of 77 complete genomes of the most representative prokaryotes of 313 identified in our samples (Supplementary Table S4). The 77 selected species constituted 93% of all identified bacteria/archaea, and each of the selected species accounted for at least 10% in at least 1 sample (Supplementary Table S4A). The remaining species represented only 7% of the total microbial DNA, and they typically accounted for less than 1% of an individual sample. Subsequently, for each sample, we mapped all reads against (i) all 77 selected genomes; (ii) a subset of 55 environmental bacteria genomes; (iii) a subset of 14 oral bacteria genomes; (iv) a subset of 3 gut bacteria and archaea genomes; (v) a subset of 5 potential pathogen genomes; and (vi) a subset consisting of all human-related bacterial genomes (22 genomes; oral, gut, and pathogens). Additionally, we mapped reads against a reference human genome to compare in each sample the level of DNA damage in human and microbial genomes. The comparison of DNA damage signatures in human and microbial DNA in individual samples is presented in Supplementary Fig. S9 and Supplementary Fig. S10.

**Figure 7:**
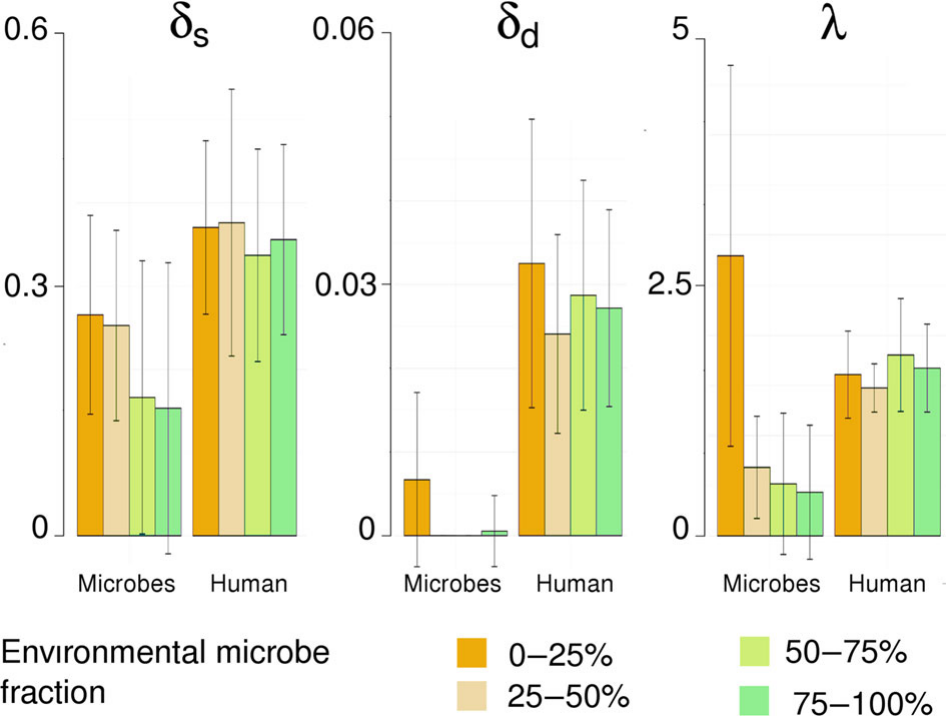
The DNA damage in samples with different fractions of environmental bacteria/archaea. Barplots indicating deamination rate in single-stranded DNA overhangs (δ_s_) and double-stranded DNA fragments (δ_d_) in microbial (left-hand site) and human DNA (right-hand site), grouped based on the fraction of environmental bacteria/archaea in the sample and the length of single-stranded DNA overhangs (λ, expressed as: 1/λ-1) calculated for 77 representative bacteria/archaea and endogenous human aDNA. Samples were grouped based on the fraction of environmental bacteria/archaea in a sample (0–25%, 25–50%, 50–75%, and 75–100%).

As shown in Fig. 7, the average DNA damage determined for all 77 microbial genomes decreased with the increase in environmental bacteria fractions. Microbial DNA damage values differed significantly between samples with different fractions of environmental components (1-way ANOVA: (δ_s_) *P* = 0.0413; (δ_d_) *P* = 0.0001; (1/λ-1) *P* < 0.0001). The samples with the lowest (<25%) contribution of environmental bacteria displayed the highest level of microbial DNA damage (on average: δ_s_ = 0.2643, δ_d_ = 0.0067, 1/λ-1 = 2.7933), comparable with those observed for endogenous human aDNA (on average: δ_s_ = 0.3571, δ_d_ = 0.0279, 1/λ-1 = 1.6667). Noticeably, the damage of human aDNA did not depend on the amount of environmental bacteria in a sample (1-way ANOVA: (δ_s_) *P* = 0.8630; (δ_d_) *P* = 0.3530; (1/λ-1) *P* = 0.4770) (Fig. 7).

**Figure 8:**
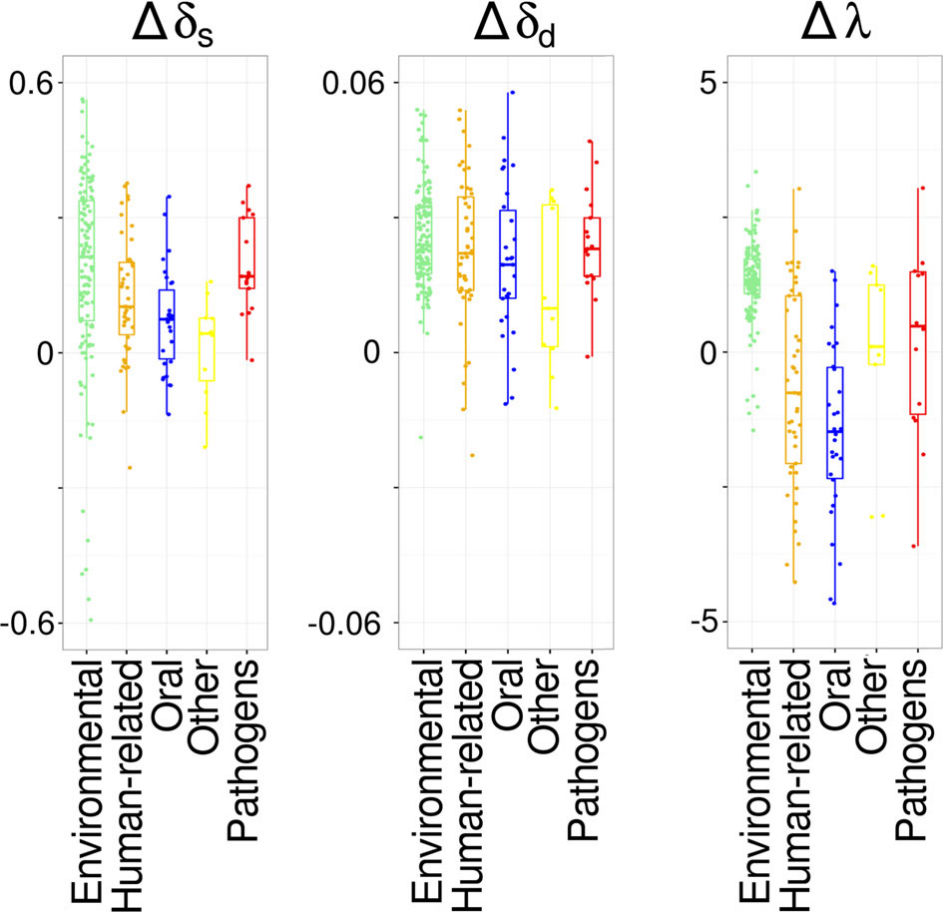
The differences of DNA damage levels (Δδ_s_, Δδ_d_, Δλ, expressed as: Δ(1/λ-1)) of bacteria/archaea species belonging to the 5 groups (environmental, all human-related, oral, gut, and pathogen) in comparison to damage levels in human aDNA. Boxes, whiskers, and dots represent the distribution of differences in DNA damage levels of particular bacterial/archaeal groups. Each dot represents the difference in an individual sample. The color legend is the same as in Fig. 4 (all human-related species are in orange).

In the next step, for each sample, we calculated the DNA damage values separately for the following groups of bacterial species: (i) environmental; (ii) all human-related; (iii) oral; (iv) gut; and (v) potential pathogens. We compared these values with corresponding values determined for the endogenous human aDNA in the same sample. As is shown in Fig. 8, the highest differences between the levels of human and microbial DNA damage were observed for environmental bacteria that showed very little DNA damage (on average: Δδ_s_ = 0.1767; Δδ_d_ = 0.0264; Δ(1/λ-1) = 1.3089). It is also shown in Fig. 8 that the DNA damage of human-related species is similar to that observed for human aDNA (average: Δδ_s_ = 0.1224; Δδ_d_ = 0.0278; Δ(1/λ-1) = –0.8805). The variations in the obtained values may result from different rates of microbial DNA decay as well as from misclassification of some microbial species.

**Figure 9:**
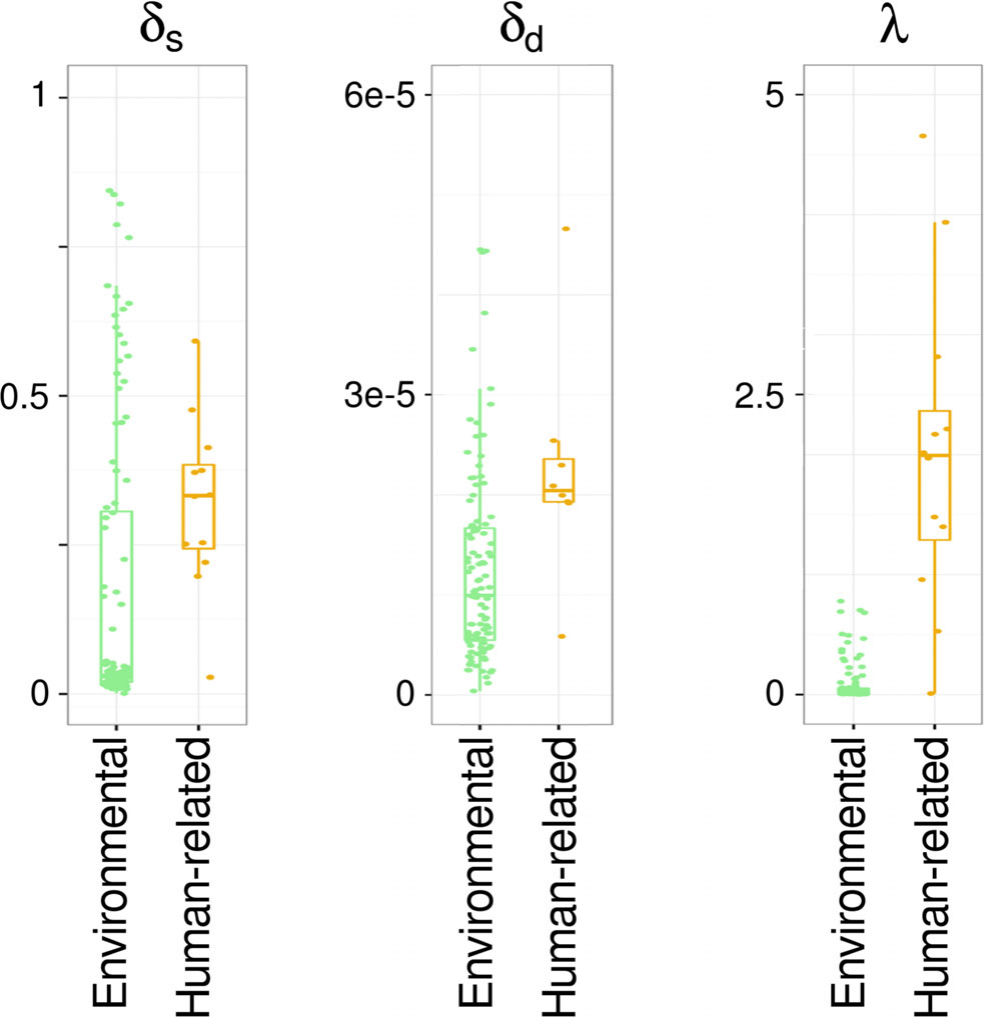
DNA damage level (δ_s_, δ_d_, 1/λ-1) in environmental and all human-related *Actinobacteria* species. Boxes, whiskers, and dots represent the distribution of DNA damage levels in particular samples. The color legend is the same as in Fig. 4 (all human-related species are in orange).

*Actinobacteria*, as well as all classes known to be non-spore-forming, are more durable than other bacteria [62]. Thus, we used *Actinobacteria* (the most abundant class in our study) to analyze whether the differences between environmental and human-related species in DNA damage levels were influenced by different rates of damage in various microbe types. Within the human-related group (oral), we identified 3 species belonging to *Actinobacteria*, present in 12 samples in >5%. Within the environmental group, we identified 12 species, present in 104 samples as >5%. The DNA damage pattern comparison again showed a higher damage rate in human-related rather than environmental *Actinobacteria* (t-test: (δ_s_) *P* = 0.0091; (δ_d_) *P* = 0.0299; (1/λ-1) *P* = 0.0004) (Fig. 9). This finding confirmed that the larger accumulation of DNA damage observed for human-related species was not microbe type–specific. Therefore, different DNA damage levels in environmental and human-related bacteria did not result from differences in the stability of bacterial genomes but from their age.

## Discussion

This study represents one of the most comprehensive analyses of the microbiomes that accompany ancient human skeletal remains. Accordingly, the analyzed DNA could come from (i) microorganisms that formed the human microbiome and existed in the human organism before death or (ii) environmental species that contaminated human remains or participated in the body's decomposition process.

In this study, we analyzed 161 datasets (total sequencing > 63 Bbp) collected from 7 different archaeological sites. We employed a novel approach based on a clade-specific genes analysis (MetaPhlAn2) [63]. This method relies on the database of marker sequences derived from whole genomes that unequivocally allows for the identification of microbial taxa down to the species level. Moreover, this method works not only for prokaryotes but also for all unicellular organisms and viruses. In contrast, a traditional approach based on the analysis of a singular 16S rRNA marker gene [64] is limited to the identification of bacteria/archaea at the genus level at most, thus being less accurate [65]. The applied methodology allowed us to determine the amount and type of viruses and fungi as well as bacteria and archaea in the analyzed samples. Notably, we showed that shallow sequencing (the average number of reads per analyzed sample was ∼5 million) permitted retrieval of reliable microorganism profiles. The result, validated using deeper sequencing (to 50–100 million reads), confirmed that our findings from shallow sequencing were trustworthy, although it has to be noted that the accuracy slightly decreased with the taxonomic levels (Fig. 6).

The thorough analyses of all microorganisms as well as only prokaryotes revealed that there are substantial differences between individual samples, but the differences were not characteristic for particular sample types. We showed that there was no correlation between the composition of microbial population and geographical place, sample age, or storage history. It has to be noted, however, that our results do not exclude completely the effect of storage on microbial composition. Such an effect may exist, but it is too low to be detected due to very high variation in microbial composition between individual samples. On the other hand, the high variance may suggest that pores in the teeth constitute independent variable micro-environments, some easily accessible to an exogenous DNA, while others not (or temporarily not), which promotes the stochastic and unique microbial composition. Moreover, the comparison between museum specimens (more than 20 years from excavation) and relatively freshly sampled materials suggested that the treatment applied before storage (e.g., washing) and storage itself do not influence the microorganism composition in tooth niches. Most likely, the migration of bacteria or a diffusion of microbial DNA and other microorganisms must be most intense when the remains are in direct contact with soil or water and negligible when placed in a relatively sterile environment, such as a museum deposit. These findings are of a certain importance as they indicate that studying ancient microbiome museum specimens may be as good as studying freshly discovered specimens.

Overall, we identified 25 microbial classes; the genetic material of 6 of them comprised more than 1% of all bacterial and archaeal DNA (Fig. 3A). Most of identified genera were ubiquitous bacteria belonging to the *Actinobacteria* class, such as *Brevibacterium, Kribbella, Actinoplanes*, and *Streptosporangium*, which are typically found in a wide range of soils and waters (Fig. 4). The obtained results are in line with previous findings [15, 16, 49], as well as with the common notion that DNA contamination of fossil remains comes from the soil and water. In addition, in some samples, we identified a substantial portion of microbes associated with the human body, mainly with the oral cavity, belonging predominantly to the *Clostridia* (*Eubacterium*, *Pseudoramibacter*), *Actinobacteria* (*Propionibacterium, Corynebacterium, Actinomyces*), and *Bacteroidia* (*Tannerella*) classes. Moreover, we identified 2 bacterial and 1 archaeal genera typical of the human digestive system: *Neisseria*, *Escherichia* (*Proteobacteria* class), and *Methanobrevibacter* (*Methanobacteria* class), as well as 4 potential human pathogens: *Bordetella*, *Stenotrophomonas*, *Bartonella* (*Proteobacteria* class), and *Clostridium* (*Clostridia* class).

The analyses of viruses present in the aDNA samples revealed that the substantial fraction of them accounted for 2 plant RNA viruses, whose genomes are composed of ssRNA: *Dasheen mosaic virus* (58% of all identified viruses/viroids) and *Vicia cryptic virus* (26.7%). As our NGS library preparation protocol was not designed for RNA sequencing (lack of the reverse transcription step), this result is rather unexpected and has to be interpreted with caution. Identification of RNA viruses may be potentially explained by (i) unintended reverse transcription of viral RNA either by some environmental reverse transcriptase or by DNA polymerase used for the NGS library preparation (DNA polymerase can display residual activity on RNA template, especially if the latter is in a relatively high concentration); or (ii) missmapping of some reads to markers of RNA viruses and consequently microbial misclassification. The second possibility may be enhanced by the very high genetic variability of RNA viruses. Thus, further studies are required to solve this problem.

DNA damage pattern analysis of the identified environmental and human-related microbes showed that the DNA of human-related species had significantly higher numbers of C → T and G → A substitutions, which are typical of aDNA. Moreover, their damage levels were comparable with those observed for endogenous human aDNA in the corresponding samples (see Supplementary Figs S9 and S10). According to the assumption that environmental microbes colonized archaeological bones relatively recently, DNA of environmental microbes displays a minimal amount of aDNA characteristic signatures. There is a possible bias caused by different dynamics of postmortem DNA modifications in various bacteria types [66]. It has been shown that non-spore-forming *Actinobacteria* are more durable than endospore formers such as *Bacillaceae* and *Clostridiaceae* [62]. The DNA damage analysis within the *Actinobacteria* class only revealed that human-related *Actinobacteria* species manifested aDNA damage patterns and that the environmental species showed the opposite pattern. This additional analysis supported our results and showed that the different levels of aDNA damage in environmental and human-related groups were not caused by the differences in bacterial genome stability. This also suggested that the identified human-related species may truly accompany the individual even before death. For environmental components, it seems that their DNA is relatively young and must have been acquired recently. One possible explanation is that some niches in the teeth are open and DNA exchange occurs continuously with the environment, whereas other niches are hardly accessible, so only endogenous species may reach and be preserved in these niches.

Many human pathogens belong to the same genera as environmental species [67]. For example, *Bordetella bronchiseptica* can survive in the environment and is present in a wide range of animals [68, 69]. The genus *Bordetella* also contains species that are commonly found in the environment, such as *B. petrii*. *Clostridium tetani* is known to be the causative agent of tetanus, but it is often found in soils and participates in the body's decomposition process. Hence, the identification of potential pathogens in body remains may not certainly mean that the individuals were infected with the bacterium before death. In fact, our analyses revealed that the DNA of some of the identified potential pathogens showed the DNA damage degree closer to the damage of environmental microbes than to the damage of human-associated ones.

We showed that identification of candidate bacteria/archaea species accompanying the organism before death is possible using standard aDNA extraction protocols and shallow shotgun sequencing. The use of microbial markers derived from whole genomes is crucial as aDNA typically lacks huge blocks of information and using only the 16S rRNA gene as a marker may be not sufficient.

Our results indicated that not only fresh samples but also museum specimens seem to be good sources of ancient microbial DNA. Moreover, this methodology may be employed for screening remains without visible signs of disease, which provides the huge possibility of finding ancient pathogens for further analysis. In particular, this may provide additional knowledge to the fields of epidemiology and bacterial population genomics, allowing for the investigation of the rate of bacterial evolution, and may even bring forth some information on the ancient human diet.

## Potential Implications

Here, we showed that the composition of the microbiome of archeological remains is highly variable but does not show any evident correlation with the method or duration of sample storage. That opens up a possibility to study on a wide range the microbiomes present in human and also non-human remains. We also demonstrated that it is possible to obtain reliable profiles of microbiomes from single-end shallow next-generation sequencing that allow the cutting of time and costs for any microbiome study. The presented procedures might be used as a first step of ancient pathogen identification, especially when a large set of samples with no apparent infection symptoms is considered. Finally, our studies revealed that by analyzing the DNA damage pattern, one can identify the putative ancient microorganisms present in the microbiome of archeological remains.

## Methods

### Experimental procedures

DNA extraction from teeth was performed in the ancient DNA laboratory at the Faculty of Biology, Adam Mickiewicz University, Poznan, Poland. To avoid contamination that might be introduced through laboratory manipulations, all reagents used for DNA purification (buffers, water) and small plastic materials were UV irradiated (254 nm) for 1 hour. The surface of the teeth was cleaned with 0.5–5% NaOCl, rinsed with sterile and UV-irradiated water, and exposed to UV (254 nm) for 2 hours per each site. Following UV irradiation, the roots of the teeth were drilled using Dremel®, and bone powder was collected to sterile tubes (2 ml) and digested for 48 hours at 56°C in a buffer containing EDTA, UREA, and proteinase K, as described in Juras et al. [70]. After digestion, DNA was purified using the MinElute kit (QIAGEN, RRID:SCR_008539) according to Yang et al. [71] and Malmstrom et al. [72]. Genomic libraries preparation was performed as described in Meyer and Kircher [73]. The protocol comprised a blunt-end repair step. A single-stranded DNA overhanging 5^΄^- and 3^΄^-ends was filled in or removed by T4 DNA polymerase. Typical T4 DNA polymerase removes 3^΄^-overhangs and fills in 5^΄^-overhangs. Shallow sequencing was conducted following the Illumina single-end standard protocol on GAIIx using a 75-bp sequencing run. Deep sequencing was conducted following the Illumina pair-end standard protocol on GAIIx using a 100-bp sequencing run.

### Contamination control

DNA contamination from the laboratory environment and reagents was controlled through setting up negative controls during DNA extraction, genomic libraries preparation, and amplification in parallel with the samples at all experimental steps. DNA concentrations in negative controls were undetectable with Qubit dsDNA HS Assay (Thermo Fisher Scientific) and Bioanalyzer 2100 HS DNA Assay (Agilent), implying concentrations below 0.01 ng/uL. Concentrations of the libraries built from ancient human teeth were between 1.1 and 125.5 ng/uL (on average, 18.76 ng/uL). The amount of DNA in negative controls was at least 100-fold lower than for ancient samples and was not subjected to the sequencing.

### Bioinformatics procedures

All reads were trimmed, and adapters were removed using the AdapterRemoval tool (AdapterRemoval, RRID:SCR_011834) [74]. The minimal length of reads was set to 25, and the minimal base quality was set to 30.

To investigate the composition of microbial communities in each sample, we used the MetaPhlAn2 program with default settings (MetaPhlAn, RRID:SCR_004915) [46]. To avoid bias in the assessment of microorganism abundance, we mapped (using Bowtie2 [Bowtie2, RRID:SCR_005476] [75] and the recommended sensitive global alignment strategy) all reads against the MetaPhlAn2 markers database and removed PCR duplicates with Picard MarkDuplicates tool 1.82 (Picard, RRID:SCR_006525). Next, we ran MetaPhlAn2 with the option “-a” to determine all taxonomic levels.

To assess the amount of endogenous DNA, reads were mapped against human nuclear (hg19) [76] and complete mitochondrial genomes (GenBank Accession no. NC 012920.1) [77].

To investigate aDNA damage patterns, we employed mapDamage2.0 with the default settings (mapDamage, RRID:SCR_001240) [59]. All plots were generated using R 3.3.2 ggplot2 package (ggplot2, RRID:SCR_014601).

### Statistical analysis

Shannon diversity, PCA, and PCoA on 4 taxonomic levels (class, genus, family, species) were run in R (functions: diversity(), prcomp(), and pcoa(), respectively) for all identified microorganisms and for bacteria/archaea only. PCoA was run on the Jaccard, and Bray-Curtis distance tables were calculated from the taxon abundance. To determine whether low-abundance taxa (<1%) may have influenced the analysis, we also ran PCoA without them (data not shown). To determine if *k*-mers of exogenous reads might segregate samples according to their age, storage, or archeological site, we followed the approach described in Dubinkina et al. [78].

To test if certain groups displayed statistically significant differences, we applied a 1-way ANOVA, followed by a Tukey HSD and a t-test (R functions: aov(), TukeyHSD(), t.test()), as well as the following non-parametric tests: Kruskal-Wallis and Wilcoxon (R functions: kruskal.test(), wilcox.test()).

Correlation *R* was calculated as a Pearson correlation coefficient.

## Additional files

Supplementary Table S1. Summarized information on NGS datasets used within this study. The first column from the left lists sample IDs. Column 2 comprises information on C14 dating of selected samples. Column 3 and column 4 describe the depth of sequencing (number of raw and filtered reads). Columns 5 and 6 describe the reads that map to the human genome (number, percentage). Columns 7 and 8 describe reads mapping to the Metaphlan2 markers DB (number, percentage). Column 9 describes the number of reads that mapped to the prokaryotic markers only. Columns 10–16 describe the percentage (within a sample) of viruses/viroids, eukaryote, all prokaryote, environmental prokaryote, oral prokaryote, other human-related prokaryote, and potential pathogens, respectively. Columns 17–22 describe the number of identified bacterial/archaeal taxa and Shannon index on class, family, and species level, respectively.

Supplementary Table S2. Summarized information on bacterial/archaeal taxa (column 1) identified within samples (columns 5–165). Column 2–4 describe taxon gram stain type, respiratory type, and its typical habitat, respectively.

Supplementary Table S3. The information on 11 samples used for the validation of results obtained in a shallow sequencing experiment. The first column from the left lists sample IDs. Column 2 describes the total number of filtered reads. Columns 3 and 4 describe the reads that mapped to the Metaphlan2 markers DB (number, percentage). Column 5 describes the percentage of prokaryote identified in a sample. Columns 6 and 7 describe the number of bacterial/archaeal classes and the Shannon index.

Supplementary Table S4. Summarized information on 77 bacterial and archaeal species (column 1) selected for aDNA damage analysis. Columns 2–4 describe species gram stain type, respiratory type, and its habitat, respectively. Columns 5–12 describe the number of samples in which the species were present in more % than the threshold (80%, 70%, 60%, 50%, 40%, 30%, 20%, 10%, 1%, respectively). Column 13 describes the maximal percentage of a species observed. Column 14 describes the overall percentage of a species in all samples. Columns 15–154 describe the species percentage in an individual sample. A) Table summarizes the number of samples with species present in more than the threshold and their percentage with respect to the all the identified species.

aDNA_microorganisms_Figlerowicz_Supplementary_ Figures.pdf

aDNA_microorganisms_Figlerowicz_Supplementary_ Tables.xlsx

aDNA_microorganisms_Figlerowicz_Supplementary_Tables_ leg.docx

## Abbreviations

aDNA ancient DNA

dsDNA double-stranded DNA

NGS next-generation sequencing.

## Supplementary Material

GIGA-D-17-00056_Original_Submission.pdf

GIGA-D-17-00056_Revision_2.pdf

GIGA-D-17-00056_Revision_1.pdf

Response_to_Reviewer_Comments_Original_Submission.pdf

Response_to_Reviewer_Comments_Revision_1.pdf

Reviewer_1_Report_(Original_Submission).pdf

Reviewer_1_Report_(Revision_1).pdf

Reviewer_2_Report_(Original_Submission).pdf

Reviewer_2_Report_(Revision_1).pdf

aDNA_microorganisms_Figlerowicz_Supplementary_Figures.pdf

aDNA_microorganisms_Figlerowicz_Supplementary_Tables.xlsx

aDNA_microorganisms_Figlerowicz_Supplementary_Tables_leg.docx

## Availability of supporting data and materials

Other data further supporting this work can be found in the *GigaScience* repository, GigaDB [79]. The datasets supporting the conclusions of this article are available in the National Center for Biotechnology Information Sequence Read Archive repository, SRP093814 [80].
