# Supplementary material for: Comprehensive analysis of microorganisms accompanying human archaeological remains
Source: Gigascience. 2017 Jun 13;6(7):1–13. doi: 10.1093/gigascience/gix044 (PMC5965364; doi:10.1093/gigascience/gix044)
Supplement: GIGA-D-17-00056_Original_Submission.pdf [file gix044_giga-d-17-00056_original_submission.pdf]

GigaScience

Comprehensive analysis of microorganisms accompanying human archaeological remains

--Manuscript Draft--

**Manuscript Number:**	GIGA-D-17-00056
**Full Title:**	Comprehensive analysis of microorganisms accompanying human archaeological remains
**Article Type:**	Research
**Funding Information:**	Narodowe Centrum Nauki (2014/12/W/NZ2/00466) — Prof. Marek Figlerowicz
**Abstract:**	Background<br>Metagenome analyses has become a common source of information about microbial communities that occupy a wide range of niches, including archaeological specimens. It has been shown that vast majority of DNA extracted from ancient samples come from bacteria (presumably modern contaminants). However, comprehensive characterization of microbial DNA accompanying human remains has never been done.<br><br>Findings<br>We used metagenomic approaches to perform comparative analyses of microorganism communities present in 161 archaeological human remains. DNA samples were isolated from the teeth of human skeletons dated from 100 AD to 1,200 AD. The skeletons were collected from seven archaeological sites in Central Europe and stored under different conditions. The majority of identified microbes were ubiquitous environmental bacteria that most likely contaminated the host remains not long ago. We observed that the composition of microbial communities was sample-specific and not correlated with its temporal or geographical origin. Additionally, traces of bacteria and archaea typical for human oral/gut flora as well as potential pathogens were identified in two-thirds of the samples. The genetic material of human-related species, in contrast to the environmental species that accounted for the majority of identified bacteria, displayed DNA damage patterns comparable with endogenous human aDNA, which suggested that these microbes might have accompanied the individual before death.<br><br>Conclusions<br>Our study showed that the microbiome observed in an individual sample is not reliant on the method or duration of sample storage. Moreover, shallow sequencing of DNA extracted from ancient specimens and subsequent bioinformatics analysis allowed both the identification of ancient microbial species, including potential pathogens, and their differentiation from contemporary species that colonized human remains more recently.
**Corresponding Author:**	Marek Figlerowicz<br>Institute of Bioorganic Chemistry PAS<br>Poznań, WLKP POLAND
**Corresponding Author Secondary Information:**	
**Corresponding Author's Institution:**	Institute of Bioorganic Chemistry PAS
**Corresponding Author's Secondary Institution:**	
**First Author:**	Anna Philips
**First Author Secondary Information:**	
**Order of Authors:**	Anna Philips<br>Ireneusz Stolarek

Powered by Editorial Manager® and ProduXion Manager® from Aries Systems Corporation

	Bogna Kuczkowska
	Anna Juras
	Luiza Handschuh
	Janusz Piontek
	Piotr Kozłowski
	Marek Figlerowicz
**Order of Authors Secondary Information:**	
**Opposed Reviewers:**	
**Additional Information:**	
**Question**	**Response**
Are you submitting this manuscript to a special series or article collection?	Yes
Please select an option from the menu: as follow-up to "Are you submitting this manuscript to a special series or article collection?"	Functional Metagenomics
**Experimental design and statistics**<br><br>Full details of the experimental design and statistical methods used should be given in the Methods section, as detailed in our Minimum Standards Reporting Checklist. Information essential to interpreting the data presented should be made available in the figure legends.<br><br>Have you included all the information requested in your manuscript?	Yes
**Resources**<br><br>A description of all resources used, including antibodies, cell lines, animals and software tools, with enough information to allow them to be uniquely identified, should be included in the Methods section. Authors are strongly encouraged to cite Research Resource Identifiers (RRIDs) for antibodies, model organisms and tools, where possible.<br><br>Have you included the information requested as detailed in our Minimum Standards Reporting Checklist?	Yes
**Availability of data and materials**<br><br>All datasets and code on which the	Yes

conclusions of the paper rely must be either included in your submission or deposited in publicly available repositories (where available and ethically appropriate), referencing such data using a unique identifier in the references and in the "Availability of Data and Materials" section of your manuscript.<br><br>Have you have met the above requirement as detailed in our Minimum Standards Reporting Checklist?	

Powered by Editorial Manager® and ProduXion Manager® from Aries Systems Corporation

Manuscript

Click here to download Manuscript aDNA_microorganisms_Figlerowicz_Revised.docx

Comprehensive analysis of microorganisms accompanying human archaeological remains

Anna Philips[1], Ireneusz Stolarek[1], Bogna Kuczkowska[1], Anna Juras[2], Luiza Handschuh[1,3,4], Janusz Piontek[2], Piotr Kozlowski[1,4,*], Marek Figlerowicz[1,5,*]

[1] European Center for Bioinformatics and Genomics, Institute of Bioorganic Chemistry, Polish Academy of Sciences, Poznan, 61-704, Poland

[2] Department of Human Evolutionary Biology, Institute of Anthropology, Faculty of Biology, Adam Mickiewicz University in Poznan, 61-614 Poznan, Poland

[3] Department of Hematology and Bone Marrow Transplantation, University of Medical Sciences, 60-569 Poznan, Poland

[4] Institute of Technology and Chemical Engineering, Poznan University of Technology, Poznan, 60-965, Poland

[5] Institute of Computing Science, Poznan University of Technology, Poznan, 60-965, Poland

* To whom correspondence should be addressed. MF: Tel: (+48) 61 852 8919; Email: marekf@ibch.poznan.pl. Correspondence may also be addressed to PK: Tel: (+48) 61 852 8503; Email: kozlowp@yahoo.com.

ABSTRACT

Background

Metagenome analyses has become a common source of information about microbial communities that occupy a wide range of niches, including archaeological specimens. It has been shown that vast majority of DNA extracted from ancient samples come from bacteria (presumably modern contaminants). However, comprehensive characterization of microbial DNA accompanying human remains has never been done.

Findings

We used metagenomic approaches to perform comparative analyses of microorganism communities present in 161 archaeological human remains. DNA samples were isolated from the teeth of human skeletons dated from 100 AD to 1,200 AD. The skeletons were collected from seven archaeological sites in Central Europe and stored under different conditions. The majority of identified microbes were ubiquitous environmental bacteria that most likely contaminated the host remains not long ago. We observed that the composition of microbial communities was sample-specific and not correlated with its temporal or geographical origin. Additionally, traces of bacteria and archaea typical for human oral/gut flora as well as potential pathogens were identified in two-thirds of the samples. The genetic material of human-related species, in contrast to the environmental species that accounted for the majority of identified bacteria, displayed DNA damage patterns comparable with endogenous human aDNA, which suggested that these microbes might have accompanied the individual before death.

Conclusions

Our study showed that the microbiome observed in an individual sample is not reliant on the method or duration of sample storage. Moreover, shallow sequencing of DNA extracted from ancient specimens and subsequent bioinformatics analysis allowed both the identification of ancient microbial species, including potential pathogens, and their differentiation from contemporary species that colonized human remains more recently.

Keywords

Microbiome, ancient DNA, NGS, metagenomics

BACKGROUND

During the last two decades, a number of methods that permit isolation and sequencing of ancient DNA (aDNA) extracted from archaeological specimens have been elaborated. As a result, several complete genome sequences of long-dead organisms have been determined [1-5]. Typically, aDNA is sampled from teeth or bones as these are the densest tissues in vertebrates, which supports the preservation of aDNA in crystal aggregates [6, 7]. Ancient remains are usually deposited in soils for decades, so DNA extracted is a mix of host DNA fragments and DNA form different organisms inhabiting the environment. To avoid the contamination that is usually present on bone/teeth surfaces (e.g., modern human, bacterial, fungal or plant DNA), aDNA is sampled from interior parts, where amount of aDNA is the highest. Despite applying rigorous DNA extraction protocols, the endogenous aDNA usually constitutes much less than 5% of the total extracted DNA, e.g., 1–5% for a Neanderthal [2] and 4% for a Mal'ta boy (24,000-year-old human) [8]. Of the remaining DNA, typically >>95% is DNA of different microorganisms that have colonized the remains and have been acquired from the environment. When younger remains are considered (100–200 years old), the amount of endogenous aDNA is not much higher [9]; however, it is possible to obtain a sample containing even up to 70% of endogenous aDNA [4, 10]. This is because the preservation of DNA depends on many environmental factors [11, 12]. For example, cold temperatures [13, 14], microclimate of caves where remains have been buried [11], or swampy sediments [12] are known to enhance DNA stability. Moreover, it has been shown that the vast majority of DNA isolated from archaeological human remains belongs to bacteria that have colonized the remains [15, 16]. Bacteria amplify the porosity of bone and teeth [17, 18], making it more accessible to water, which may lead to so-called endogenous aDNA leaching [19] and it replacing by exogenous DNA.

Some target enrichment procedures have been proposed to increase the amount of endogenous aDNA [20-24] and among them is the two-step digestion method [14, 25, 26]. Interestingly, Orlando and colleagues showed that two-step digestion does not influence the composition of bacterial communities (e.g., is the same in aDNA samples obtained after the first and second digestion runs) [9]. This observation suggests that niches exist deep within the bones and teeth. The environmental bacteria may rich these niches and preserve there.

Metagenome analysis has become a common source of information about microbial communities that occupy a wide range of niches. Until today, environmental components [27] as well as flora of

different human sites [28], e.g., oral [29, 30], skin [31] or intestinal [32-35], have been well characterized. In our study, we used this approach to analyze microorganisms that accompany archaeological human remains, which until now have been poorly profiled. Prior findings are limited to the rough identification of environmental bacteria [16] or concern a singular species, usually pathogenic. In the latter cases, the analyses were usually undertaken after the identification of visible symptoms of past disease [36-38]. Efforts have also been undertaken to characterize human mummy intestinal [39] and colon [40] microbes, as well as ancient oral microbiome ([41-44]). They showed that aDNA of species that colonized the organism before its death may be obtained. However, comprehensive characterization of microbial DNA accompanying human remains has never been done.

The current study was performed to characterize microorganisms associated with human archaeological remains. We used shotgun sequencing of DNA isolated from 161 human teeth collected from seven archaeological sites dated from 100 AD to 1,200 AD and stored under different conditions (e.g., museum or grave). For each individual sample its microbiome was determined using MetaPhlAn2 (Metagenomic Phylogenetic Analysis) based on multiple specific marker sequences derived from the genomes of microorganisms [45, 46]. Within this study, we focused on bacteria and archaea, which are known to constitute the majority of exogenous DNA in human archaeological remains [15, 16]. We checked whether microbial communities associated with specimens from different archaeological sites or of different ages were taxonomically and functionally distinct. We also attempted to identify microbes that may accompany the organism even before death and to distinguish bacteria/archaea that stem from *post-mortem* contamination from those of original flora by studying their DNA damage patterns.

DATA DESCRIPTION

We analyzed 161 human bone samples collected from seven archaeological sites in Central Europe (Figure 1 A). As shown in Table 1, the samples differed by age [Roman Age group (KO and MZ) or Medieval group (GO, SI, NA, ME, and LO)] and by storage conditions [specimens that were in museum deposits for at least 20 years (long deposit: KO, MZ, SI, NA, and GO), relatively freshly discovered specimens (stored in museum deposit <5 years, short deposit: LO) or samples taken directly from an archaeological site (Arch. site: ME)]. Carbon isotope dating of the selected samples

correlated well with dating based on archaeological analysis (see Supplementary Table 1).

Ancient DNA was always extracted from the roots of teeth. We drilled those parts of the roots which include both dentine and cementum. In all cases enamel and cementum were preserved. Subsequently, all DNA samples were subjected for shallow NGS sequencing with the usage of an Illumina single-end standard protocol (including blunt-end DNA repair) and 75 bp sequencing run. Altogether, 846.5 million reads were obtained. On average, 98.6% of reads passed trimming and quality filtration. After filtration, for 161 samples, the average number of reads per sample was 5,143,975 (median: 4,730,243, range: 34,857 to 26,055,295). In further analysis, we removed eight samples that did not meet the arbitrary criterion of minimal raw reads number (<1 million). The average number of reads did not differ substantially between archaeological sites (one way ANOVA: p=0.1120) or type of storage (t-test: p=0.4179), Figure 1 B. Detailed information on each sample is summarized in Supplementary Table 1.

All reads were mapped to the reference human genome, and the percentage of human reads was determined for each sample. As shown in Figure 1 C, the fraction of human aDNA ranges from 0.01% to 91.9%; however, in most cases (100 samples), it was less than 5%. Nine samples had more than 50% of human aDNA content. No evident differences in the amount of human aDNA content were observed for different archeological sites (one way ANOVA: p=0.1580), older (KO, MZ) and younger (SI, NA, ME, GO, LO) sample sets (t-test: p=0.1773) as well as for freshly recovered and stored in museum samples (t-test: p=0.6680).

ANALYSES

Microbiomes of human archaeological remains

To characterize the microbiomes of analyzed archaeological samples, we used MetaPhlAn2. The program identifies bacteria/archaea, viruses/viroids and unicellular eukaryotes using homology-based classification of NGS reads by alignment with predefined taxa-specific marker sequences [45]. The number of reads mapped to MetaPhlAn2 markers ranged from 708 (sample KO_014) to 95,950 (sample KO_006). Two samples with <1,000 reads mapped to the marker sequences were removed from further analyses as the marker coverage is crucial for proper microorganisms detection ([46]).

For the remaining 151 samples our analyses (Figure 2 A) showed that the majority of reads mapped to bacterial or archaeal markers (76.4%) and 23.4% to virus/viroid markers. The remaining

0.2% constituted eukaryotes (present in 13 samples; 0.6–8.2%), which were subsequently identified as fungi, protists or protozoa. The contribution of the particular types of microorganisms differed substantially between individual samples (in 12 samples, we found only bacteria; in sample KO_28, only viruses were identified; Figure 2 C). However, these differences did not correlate with archaeological site (MANOVA: p=0.0532, Figure 2 B), sample age (MANOVA: p=0.2054) or storage conditions (MANOVA: p=0.7672) .

The virus fraction varied from 0.1% to 99% between samples. Analysis of virus taxa showed that most of them were associated with plants; hence, we reasoned that they may have been acquired from the environment and were possibly indigenous flora. The most abundant viruses: *Dasheen mosaic virus* (58% of all identified viruses/viroids) and *Vicia cryptic virus* (26.7%) are both known to infect plants. Five viruses and one viroid constituted less than 2.5% each of all identified viruses/viroids, and also all were found to be associated with plant genera (*Ageratum, Sauropus, Cichorium* or *Malvastrum*). The remaining viruses were of low-abundance (<1%) and were usually present in no more than a single sample. It is also noteworthy, that we identified within our samples *Propionibacterium* phage - dsDNA virus that is associated with oral microbiome [47, 48]. Detailed information on the microorganism composition in individual samples is available in Supplementary Table 2.

Characterization of bacteria and archaea in human archaeological remains

In the next step, we focused on the prokaryotic component of the analyzed microbiomes. We decided to exclude from this analysis samples with a very high fraction of viruses/viroids. As a result, 11 samples with less than 1,000 reads mapping exclusively to bacterial/archaeal MetaPhlaAn2 marker sequences were removed as did not ensured a reliable microbiome profiling.

Altogether, 25 bacterial and 4 archaeal classes were identified in exogenous DNA of the analyzed samples, and among them, 6 bacterial classes accounted for >1% of identified bacteria/archaea. The most abundant classes were *Actinobacteria* (average 57%; range 0.18–98.9%), 3 classes of *Proteobacteria* [*Alphaproteobacteria* (6%; 0–65.5%), *Betaproteobacteria* (7%; 0–83.6%), *Gammaproteobacteria* (12%; 0–95.4%)], *Acidobacteria* (5%; 0–39.7%), and *Clostridia* (4%; 0–76.8%) (Figure 3 A). Although most of the bacteria belonging to the first five classes are typically found in the environment (wide range of soils, waters) [27, 49], some of their taxa were human flora components.

For example, *Corynebacterium matruchotii* (*Actinobacteria*) [50] and *Lautropia mirabilis* (*Betaproteobacteria*) [51, 52] represented more than 5% of the DNA in 4 samples: KO_046b, NA_121, NA_123, LO_166 and KO_005, KO_006, KO_046b, LO_166, respectively (Supplementary Table 2). *Clostridia* and *Bacteroidetes* are known to include many species inhabiting the human oral cavity or intestines [29]. Additionally, we found, in individual samples, markers characteristic for human pathogens, e.g., *Pseudoramibacter alactolyticus* (*Clostridia*) in sample MZ_88 [53] and *Bordetella parapertussis (Betaproteobacteria) [54]* in sample SI_084; *Clostridium sordellii* and *Clostridium tetani* [55, 56] (*Clostridia*) were found in 2 samples and 1 sample, respectively. Prokaryotic profiles differed substantially between individual samples (Figure 3 C) but did not differ between specific archaeological sites (MANOVA: p=0.3650, Figure 3 B), sample ages (MANOVA: p=0.3550) or storage conditions (MANOVA: p=0.4729). Similar high variation between individual samples and lack of specificity to archaeological sites were observed when prokaryotes were divided into groups based on gram +/- type (MANOVA: p=0.4364) or oxygen requirements (aerobic, facultative aerobic, anaerobic, facultative anaerobic, MANOVA: p=0.5726); see Supplementary Figure 1, Supplementary Figure 2.

The identification of singular prokaryotic taxa that are human- rather than environment-related motivated us to determine the fraction of microbes potentially associated with humans. All identified bacteria and archaea were divided on a genus level, into 2 groups: environmental and human-related. The latter was further divided into 3 subgroups: oral, potential pathogens and other (mostly gut). The genus characteristics were inferred based on the features of species identified by MetaPhlAn2. A genus was classified as human-related only if all species of this genus identified in our samples were human-related. The analysis showed that the majority (85.19%) of all bacteria/archaea were environmental (coming from soil and/or water); however, a substantial fraction of the investigated taxa (14.81%) were human-related, including 12.43% of microbes typical for human oral flora, 1.33% of potentially pathogenic bacteria and 1.05% of other (see Figure 4 A, B). As shown in Figure 4 C, the fraction of human-related genera varied significantly among samples, and some of these genera constituted most of the exogenous DNA. Although the fraction of human-related genera did not differ significantly between archaeological sites (one way ANOVA: p=0.7480), it was noteworthy that this fraction was highest in NA, the archaeological site dated to the Middle Ages, from which the samples had been stored in a deposit for more than 20 years (see Figure 4 B). Interestingly, there was no relation between prevalence of human-related microbes and the levels of viruses/viroids accumulation

or the level of endogenous human aDNA (see Supplementary Table 1). The identification of human-related species in ancient remains raised a question whether some of them accompanied the individual even before death.

Among all samples, the most frequent genera were the soil bacteria *Brevibacterium* (8.5% of all; present in 53 samples >1%; max. 71%) and *Kribbella* (8.4% of all; present in 60 samples >1%; max. 70%). The most abundant oral genera were *Bacteroidetes (*1.6% of all; present in 23 samples >1%; max. 28%), *Desulfobulbus (*1.4% of all; present in 25 samples >1%; max. 44%) and *Eubacterium* (1.4% of all; present in 20 samples >1%; max. 32%). *Methanobrevibacter* (0.8% of all; in 7 samples >1%; max. 34%), typically found in the human digestive system, was the most abundant taxon in other human-related group; *Bordetella* was the most abundant taxon classified as a potential human pathogen (*B. pertussis* is known to cause pertussis; 1.2% of all; in 16 samples >1%; max. 60%).

In general, 89% of the analyzed prokaryotes were aerobic or facultative aerobic (Supplementary Figure 1), and 63% were gram-positive (Supplementary Figure 2). However, in the human-related group only (Table 2), the percentage of aerobic or facultative aerobic taxa was smaller (24%, 49% and 54% for oral, pathogen, and other groups, respectively). Additionally, we found that the gram-negative prokaryotes dominated in the oral group (55%) and gram-positive prokaryotes in the other human-related group (70%). This slight dominance of gram-negative taxa in the oral group might be caused by lysozyme presence in an oral cavity that preferentially protects against gram-positive bacteria [57]. We additionally noticed that gram-negative species dominated (68%) in the potential pathogen group. These characteristics seem very useful for preliminary assessment of bacterial populations accompanying human remains.

To further investigate whether the prokaryotic profile permits classification of individual samples into specific groups (e.g., samples of similar age or storage conditions or samples from the same archaeological site), we performed Principal Coordinates Analysis (PCoA, Jaccard distance) on four taxonomic levels (class, family, genus, and species; Figure 5). Samples grouped into one big cluster in graphs created on all taxonomic levels. In the PCoA graphs generated on the family, genus and species levels, there was one more significantly smaller cluster visible. Importantly, none of these clusters segregated samples according to the abovementioned features (age, storage, and site). Principal Component Analysis (PCA, see Supplementary Figure 3) and the Shannon diversity index

(see Supplementary Table 1) again revealed high variation between individual samples at all analyzed taxonomic levels but did not show separation by sample source (species level, one way ANOVA: p=0.5660), sample age (species level, t-test: p=0.5535) or storage type (species level, t-test: p= 0.3516). We also tested a hypothesis that the occurrence of some human-related or environmental bacteria might be associated with archeological sites. We performed PCA analyses (Supplementary Figure 4) and hierarchical clustering (Supplementary Figure 5) on selected bacterial genera and found out that neither human-related nor environmental microbes segregated samples according to the archeological site, age or storage type.

Lastly, in order to confirm that the major source of microbes observed in human archeological samples was the environment we compared their microbiomes with the microbiomes of human [58] and soils [27] by PCoA at the genus level (See Supplementary Figure 6).

Validation of data obtained using shallow sequencing

All results presented above were obtained with the use of datasets generated by relatively shallow sequencing (on average ~5 million reads per sample). To check the reliability of our results, we determined for the selected samples to what extent the composition of microbiomes (on the class level) is affected by the depth of sequencing. For this analysis, we used 11 representative samples differentiated in terms of: (1) filtered reads number obtained in the shallow sequencing experiment (~2-8 million), (2) number of reads mapped to the MetaPhlAn2 markers (~1–60 thousand), and (3) prokaryotic fraction (~10–90%). Eight samples were sequenced to the depth of ~50 million reads and 3 to the depth of ~100 million reads. Subsequently, we ran a MetaPhlAn2 profiling analysis on deep sequencing datasets. As expected, the total number of filtered reads as well as the number of reads mapping to the MetaPhlAn2 marker sequences increased significantly (about nine fold); however, the Shannon diversity indexes and microbial compositions remained intact (correlation R: 0.91-0.99, Figure 6 A, Supplementary Table 3). We obtained similar results when we analyzed three other taxonomic levels with somehow decreasing R with the depth of taxonomic level (average R: 0.96, 0.90, 0.88, 0.78 for class, family, genus and species levels, respectively Figure 6 B, Supplementary Figure 7). It is noteworthy that sample KO_030, second lower in the number of raw reads, displayed very low correlation R=0.35 on a species level when results obtained based on shallow sequencing (~2.6 million reads) and deep sequencing (47 million reads) were compared (Supplementary Figure 7,

Supplementary Table 3). Overall, the correlation coefficient R and statistical significance ($p<0.0001$ in most cases, see Supplementary Figure 7) values were still very high and confirmed that the microbial profiles obtained based on the shallow sequencing datasets are reliable and do not change significantly when datasets generated in much deeper sequencing are used to establish them.

Analysis of age-related aDNA damage patterns

Finally, to verify whether identified human-related prokaryotes are ancient species that colonized the human body before death or are modern contaminants, we analyzed the signatures of age-related DNA damage. Age-related DNA damage was evaluated with the usage of mapDamage2.0 [59], which simulates the posterior distribution of (1) deamination in single strand DNA, ssDNA (δ_s), (2) deamination in double strand DNA, dsDNA (δ_d), and (3) the level of DNA fragmentation (λ, represented as: $1/\lambda$-1). [60, 61].

For this analysis, we used sequences of 77 complete genomes of the most representative prokaryotes of 313 identified in our samples (Supplementary Table 4). The 77 selected species consisted of 93% of all identified bacteria/archaea, and each of the selected species accounted for at least 10% in at least one sample (Supplementary Table 4A). The remaining species represented only 7% of the total microbial DNA, and they typically accounted for less than 1% of an individual sample. Subsequently, for each sample, we mapped all reads against (1) all 77 selected genomes; (2) a subset of 55 environmental bacteria genomes; (3) a subset of 14 oral bacteria genomes; (4) a subset of three gut bacteria and archaea genomes; (5) a subset of five potential pathogen genomes; and (6) a subset consisting of all human-related bacterial genomes (22 genomes; oral, gut and pathogens). Additionally, we mapped reads against a reference human genome to compare in each sample the level of DNA damage in human and microbial genomes. The comparison of DNA damage signatures in human and microbial DNA in individual samples are presented in Supplementary Figure 8 and Supplementary Figure 9.

As shown in Figure 7, the average DNA damage determined for all 77 microbial genomes decreased with the increase in environmental bacteria fractions. Microbial DNA damage values differed significantly between samples with different fractions of environmental components (one way ANOVA: (δ_s) $p=0.04130$; (δ_d) $p=0.00012$; ($1/\lambda$-1) $p<0.0001$). The samples with the lowest (<25%) contribution of environmental bacteria displayed the highest level of microbial DNA damage (on

average, δ_s=0.2643, δ_d=0.0067, 1/λ-1=2.7933), comparable with those observed for endogenous human aDNA (on average: δ_s=0.3571, δ_d=0.0279, 1/λ-1=1.6667). Noticeably, the damage of human aDNA did not depend on the amount of environmental bacteria in a sample (one way ANOVA: (δ_s) p=0.8630; (δ_d) p=0.3530; (1/λ-1) p=0.4770, Figure 7).

In the next step, for each sample, we calculated the DNA damage values separately for the following groups of bacterial species: (1) environmental; (2) all human-related; (3) oral; (4) gut and (5) potential pathogens. We compared these values with corresponding values determined for the endogenous human aDNA in the same sample. As is shown in Figure 8, the highest differences between the levels of human and microbial DNA damage were observed for environmental bacteria that showed very little DNA damage (average: $\Delta\delta_s$=0.1767; $\Delta\delta_d$=0.0264; Δ(1/λ-1)=1.3089). It is also shown in Figure 8 that the DNA damage of human-related species is similar to that observed for human aDNA (average: $\Delta\delta_s$=0.1224; $\Delta\delta_d$=0.0278; Δ(1/λ-1)=-0.8805). The variations in the obtained values may result from different rates of microbial DNA decay as well as from misclassification of some microbial species.

Actinobacteria, as well as all classes known to be non-spore forming, are more durable than other bacteria [62]. Thus, we used *Actinobacteria* (the most abundant class in our study) to analyze whether the differences between environmental and human-related species in DNA damage levels were influenced by different rates of damage in various microbe types. Within the human-related group (oral), we identified 3 species belonging to *Actinobacteria* present in 12 samples in >5%. Within the environmental group, we identified 12 species, present in 104 samples as >5%. The DNA damage patterns comparison again showed a higher damage rate in human-related rather than environmental *Actinobacteria* (t-test: (δ_s) p=0.0091; (δ_d) p=0.02987; (1/λ-1) p=0.0004, Figure 9). This finding confirmed that the larger accumulation of DNA damage observed for human-related species was not microbe type-specific. Therefore, different DNA damage levels in environmental and human-related bacteria did not result from differences in the stability of bacterial genomes but from their age.

DISCUSSION

This study represents one of the most comprehensive analyses of microbiomes that accompany ancient human skeletal remains. Accordingly, the analyzed DNA could come from (1) microorganisms that formed human microbiome and existed in human organism before death or (2) environmental

species that contaminated human remains.

In this study, we analyzed 161 datasets (total sequencing >63 Bbp) collected from seven different archaeological sites. We employed a novel approach based on a clade-specific genes analysis (MetaPhlAn2, [63]). This method relies on the database of marker sequences derived from whole genomes that unequivocally allows for the identification of microbial taxa down to the species level. Moreover, this method works not only for prokaryotes but also for all unicellular organisms and viruses. In contrast, a traditional approach based on the analysis of a singular 16S rRNA marker gene [64] is limited to the identification of bacteria/archaea at the genus level at most, thus being less accurate [65]. The applied methodology allowed us to determine the amount and type of viruses and fungi as well as bacteria and archaea in the analyzed samples. Notably, we showed that shallow sequencing (the average number of reads per analyzed sample was ~5 million) permitted retrieval of reliable microorganism profiles. The result validated using deeper sequencing (to 50–100 million reads) confirmed that our findings from shallow sequencing were trustworthy, although it has to be noted, that the accuracy slightly decreased with the taxonomic levels (Figure 6).

The thorough analysis of all microorganisms as well as only prokaryotes revealed that there are substantial differences between individual samples, but the differences were not characteristic for particular sample types. We showed that there was no correlation between the composition of microbial population and geographical place, sample age or storage history. This may suggest that pores in teeth constitute independent micro-environments, some easily accessible to an exogenous DNA, while others not (or temporarily not), which promotes the stochastic and unique microbial composition. Moreover, the comparison between museum specimens (more than 20 years from excavation) and relatively freshly sampled materials suggested that the treatment applied before storage (e.g., washing) and storage itself do not influence the microorganism composition in the teeth niches. Most likely, the migration of bacteria or a diffusion of microbial DNA and other microorganisms must be most intense when the remains are in direct contact with soil or water and negligible when placed in a relatively sterile environment, such as a museum deposit. These findings are of certain importance, as they indicate that to study ancient microbiome museum specimens may be as good as freshly discovered specimens.

Overall, we identified 25 microbial classes, genetic material of 6 of them consisted more than 1% of all bacterial and archaeal DNA (Figure 3 A). Most of identified genera were ubiquitous bacteria

belonging to the *Actinobacteria* class, such as *Brevibacterium, Kribbella, Actinoplanes* and *Streptosporangium,* which are typically found in a wide range of soils and waters (Figure 4). The obtained results are in line with previous findings ([15, 16, 49]) as well as with the common notion that DNA contamination of fossil remains comes from the soil and water. In addition, in some samples, we identified a substantial portion of microbes associated with the human body, mainly with the oral cavity, belonging predominantly to *Clostridia* (*Eubacterium*, *Pseudoramibacter*), *Actinobacteria* (*Propionibacterium, Corynebacterium, Actinomyces*) and *Bacteroidia* (*Tannerella*) classes. Moreover, we identified two bacterial one archaeal genera typical to the human digestive system, *Neisseria*, *Escherichia* (*Proteobacteria* class) and *Methanobrevibacter* (*Methanobacteria* class) as well as four potential human pathogens, *Bordetella*, *Stenotrophomonas*, *Bartonella* (*Proteobacteria* class) and *Clostridium* (*Clostridia* class).

DNA damage patterns analysis of the identified environmental and human-related microbes showed that the DNA of human-related species had significantly higher numbers of C→ T and G → A substitutions, which are typical for aDNA. Moreover, their damage levels were comparable with those observed for endogenous human aDNA in the corresponding samples (See Supplementary Figure 8, Supplementary Figure 9). According to the assumption that environmental microbes colonized archaeological bones relatively recently, DNA of environmental microbes displays a minimal amount of aDNA characteristic signatures. There is a possible bias caused by different dynamics of *post-mortem* DNA modifications in various bacteria types [66]. It has been shown that non-spore-forming *Actinobacteria* are more durable than endospore-formers such as *Bacillaceae* and *Clostridiaceae* [62]. The DNA damage analysis within the *Actinobacteria* class only revealed that human-related *Actinobacteria* species manifested aDNA damage patterns, and the environmental species showed the opposite pattern. This additional analysis supported our results and showed that the different levels of aDNA damage in environmental and human-related groups were not caused by the differences in bacterial genome stability. This also suggested that the identified human-related species may truly accompany the individual even before death. For environmental components, it seems that their DNA is relatively young and must have had been acquired recently. One possible explanation is that some niches in the teeth are open and DNA exchange occurs continuously with the environment, whereas other niches are hardly accessible, so only endogenous species may reach and be preserved in these niches.

Many human pathogens belong to the same genera as environmental species [67]. For example, *Bordetella bronchiseptica* can survive in the environment, and is present in a wide range of animals [68, 69] or *Clostridium tetani* that is known to be the causative agent of tetanus and is often found in soils. Our analyses revealed that some of them showed the DNA damage patterns closer to the environmental microbes than to the human associated ones. This observation may indicate that DNA of some potential pathogens can be acquired from the environment after the organisms death.

We showed that identification of bacteria/archaea species accompanying the organism before death is possible using standard aDNA extraction protocols and shallow shotgun sequencing. The use of microbial markers derived from whole genomes is crucial, as aDNA typically lacks huge blocks of information, and using only the 16S rRNA gene as a marker may be not sufficient.

Our results indicated that not only fresh samples but also museum specimens seem to be good sources of ancient microbial DNA. Moreover, this methodology may be employed for screening remains without visible signs of disease, which provides the huge possibility of finding ancient pathogens for further analysis. In particular, this may provide additional knowledge to the fields of epidemiology and bacterial population genomics, allowing for the investigation of the rate of bacterial evolution, and may even bring forth some information on the ancient human diet.

METHODS

Experimental procedures

DNA extraction from teeth was performed in the ancient DNA laboratory at the Faculty of Biology, Adam Mickiewicz University in Poznan. To avoid the contamination, that might be introduce through laboratory manipulations, all reagents used for DNA purification (buffers, water) and small plastic materials were UV irradiated (254 nm) per one hour. The surface of the teeth was cleaned with 0.5-5% NaOCl, rinsed with sterile and UV irradiated water, and then UV exposure (254 nm) for two hours per each site. Following UV irradiation, roots of teeth were drilled using Dremel® and bone powder was collected to sterile tubes (2 ml) and digested for 48 h in 56°C in a buffer containing EDTA, UREA and proteinase K as described in [70]. After digestion DNA was purified with MinElute kit (Qiagen) according to [71] and to [72]. Genomic libraries preparation was performed as described in [73]. The protocol comprises blunt-end repair step. A ssDNA overhanging 5'- and 3 '-ends are filled in or removed by T4 DNA polymerase. Typical T4 DNA polymerase removes 3'-overhangs and fills-in 5'-

overhangs. Shallow sequencing was conducted following the Illumina single-end standard protocol on GAIIx using a 75 bp sequencing run. Deep Sequencing was conducted following the Illumina pair-end standard protocol on GAIIx using a 100 bp sequencing run.

Contamination control

DNA contamination from the laboratory environment and reagents was controlled through setting up negative controls during DNA extraction, genomic libraries preparation and amplification in parallel with the samples at all experimental steps. DNA concentrations in negative controls were undetectable with Qubit dsDNA HS Assay (Thermo Fisher Scientific) and Bioanalyzer 2100 HS DNA Assay (Agilent), implying concentrations below 0.01 ng/uL. Concentrations of the libraries built from ancient human teeth were between 1.1 and 125.5 ng/uL (on average 18.76 ng/uL). The amount of DNA in negative controls was at least 100 fold lower than for ancient samples and was not subjected to the sequencing.

Bioinformatics procedures

All reads were trimmed and adapters were removed using the AdapterRemoval tool [74]. The minimal length of reads was set to 25 and the minimal base quality was set to 30.

To investigate the composition of microbial communities in each sample, we used the MetaPhlAn2 program with default settings [46]. To avoid bias in the assessment of microorganism abundance, we mapped (using Bowtie2 [75] and the recommended sensitive global alignment strategy) all reads against the MetaPhlAn2 markers database and removed PCR duplicates with Picard MarkDuplicates tool 1.82 (https://broadinstitute.github.io/picard/). Next, we ran MetaPhlAn2 with the option “-a” to determine all taxonomic levels.

To assess the amount of endogenous DNA, reads were mapped (Bowtie2) against human nuclear (hg19, [76]) and complete mitochondrial genomes (GenBank Accession no. NC 012920.1, [77]).

To investigate aDNA damage patterns, we employed mapDamage2.0 (with the default settings) [59]. All plots were generated using R 3.3.2 (ggplot2 package).

Statistical analysis

Shannon diversity, principal component PCA and principal coordinate PCoA analysis on 4 taxonomic

levels (class, genus, family, species) were run in R (functions: diversity(), prcomp() and pcoa(), respectively) for all identified microorganisms and for bacteria/archaea only. PCoA was run on the Jaccard and Bray-Curtis distance tables calculated from the taxon abundance. To determine whether low abundance taxa (<1%) may have influenced the analysis, we also ran PCoA without them (data not shown).

To test if certain groups displayed statistically significant differences, we applied a one-way ANOVA, followed by a Tukey HSD and a t-test (R functions: aov(), TukeyHSD(), t.test()).

AVAILABILITY OF SUPPORTING DATA AND MATERIALS

The datasets supporting the conclusions of this article are available in the NCBI Sequence Read Archive (SRA) repository, SRP093814, https://www.ncbi.nlm.nih.gov/sra/?term=SRP093814.

DECLARATIONS

List of abbreviations

aDNA – ancient DNA

dsDNA – double stranded DNA

NCBI – National Center for Biotechnology Information

NGS – next generation sequencing

ssDNA – single stranded DNA

SRA – Sequence Read Archive

Ethics approval and consent to participate

Not applicable.

Consent for publication

Not applicable.

Competing interests

The authors declare that they have no competing interests.

Acknowledgment

We thank Wioletta Nowaczewska for providing samples from Masłomęcza.

Funding

This work was supported by polish National Science Center [2014/12/W/NZ2/00466]

Authors' contributions

AP conceived the study, participated in the study design, analyzed the data, discussed the results, and wrote the manuscript; IS participated in the statistical analysis, figures preparation and submitted the datasets to SRA; BK ran preliminary Metaphlan2 analysis; AJ extracted DNA and participated in NGS library preparation; LH prepared NGS libraries and run NGS; JP: participated in results discussion; PK participated in the study design, analyzed and discussed the data and participated in drafting the manuscript; MF conceived the overall idea of the study, participated in the study design, analyzed and discussed the data, coordinated studies, was responsible for the final version of the manuscript; All authors read and approved the final manuscript.

REFERENCES

1. Fu Q, Posth C, Hajdinjak M, Petr M, Mallick S, Fernandes D, Furtwangler A, Haak W, Meyer M, Mittnik A et al: The genetic history of Ice Age Europe. Nature 2016.

2. Green RE, Krause J, Briggs AW, Maricic T, Stenzel U, Kircher M, Patterson N, Li H, Zhai W, Fritz MH et al: A draft sequence of the Neandertal genome. Science 2010, 328(5979):710-722.

3. Rasmussen M, Li Y, Lindgreen S, Pedersen JS, Albrechtsen A, Moltke I, Metspalu M, Metspalu E, Kivisild T, Gupta R et al: Ancient human genome sequence of an extinct Palaeo-Eskimo. Nature 2010, 463(7282):757-762.

4. Meyer M, Kircher M, Gansauge MT, Li H, Racimo F, Mallick S, Schraiber JG, Jay F, Prufer K, de Filippo C et al: A high-coverage genome sequence from an archaic Denisovan individual. Science 2012, 338(6104):222-226.

5. Librado P, Fages A, Gaunitz C, Leonardi M, Wagner S, Khan N, Hanghoj K, Alquraishi SA, Alfarhan AH, Al-Rasheid KA et al: The Evolutionary Origin and Genetic Makeup of Domestic Horses.

Genetics 2016, 204(2):423-434.

6. Malmstrom H, Stora J, Dalen L, Holmlund G, Gotherstrom A: Extensive human DNA contamination in extracts from ancient dog bones and teeth. Mol Biol Evol 2005, 22(10):2040-2047.

7. Salamon M, Tuross N, Arensburg B, Weiner S: Relatively well preserved DNA is present in the crystal aggregates of fossil bones. Proc Natl Acad Sci U S A 2005, 102(39):13783-13788.

8. Raghavan M, Skoglund P, Graf KE, Metspalu M, Albrechtsen A, Moltke I, Rasmussen S, Stafford TW, Jr., Orlando L, Metspalu E et al: Upper Palaeolithic Siberian genome reveals dual ancestry of Native Americans. Nature 2014, 505(7481):87-91.

9. Der Sarkissian C, Ermini L, Jonsson H, Alekseev AN, Crubezy E, Shapiro B, Orlando L: Shotgun microbial profiling of fossil remains. Mol Ecol 2014, 23(7):1780-1798.

10. Reich D, Green RE, Kircher M, Krause J, Patterson N, Durand EY, Viola B, Briggs AW, Stenzel U, Johnson PL et al: Genetic history of an archaic hominin group from Denisova Cave in Siberia. Nature 2010, 468(7327):1053-1060.

11. Ovchinnikov IV, Gotherstrom A, Romanova GP, Kharitonov VM, Liden K, Goodwin W: Molecular analysis of Neanderthal DNA from the northern Caucasus. Nature 2000, 404(6777):490-493.

12. Lawlor DA, Dickel CD, Hauswirth WW, Parham P: Ancient HLA genes from 7,500-year-old archaeological remains. Nature 1991, 349(6312):785-788.

13. Smith CI, Chamberlain AT, Riley MS, Cooper A, Stringer CB, Collins MJ: Neanderthal DNA. Not just old but old and cold? Nature 2001, 410(6830):771-772.

14. Schwarz C, Debruyne R, Kuch M, McNally E, Schwarcz H, Aubrey AD, Bada J, Poinar H: New insights from old bones: DNA preservation and degradation in permafrost preserved mammoth remains. Nucleic Acids Res 2009, 37(10):3215-3229.

15. Poinar HN, Schwarz C, Qi J, Shapiro B, Macphee RD, Buigues B, Tikhonov A, Huson DH, Tomsho LP, Auch A et al: Metagenomics to paleogenomics: large-scale sequencing of mammoth DNA. Science 2006, 311(5759):392-394.

16. Noonan JP, Hofreiter M, Smith D, Priest JR, Rohland N, Rabeder G, Krause J, Detter JC, Paabo S, Rubin EM: Genomic sequencing of Pleistocene cave bears. Science 2005, 309(5734):597-599.

17. Sampietro ML, Gilbert MT, Lao O, Caramelli D, Lari M, Bertranpetit J, Lalueza-Fox C: Tracking down human contamination in ancient human teeth. Mol Biol Evol 2006, 23(9):1801-1807.

18. Jans MME, Nielsen-Marsh CM, Smith CI, Collins MJ, Kars H: Characterisation of microbial attack on archaeological bone. J Archaeol Sci 2004, 31(1):87-95.

19. Haile J, Holdaway R, Oliver K, Bunce M, Gilbert MT, Nielsen R, Munch K, Ho SY, Shapiro B, Willerslev E: Ancient DNA chronology within sediment deposits: are paleobiological reconstructions possible and is DNA leaching a factor? Mol Biol Evol 2007, 24(4):982-989.

20. Carpenter ML, Buenrostro JD, Valdiosera C, Schroeder H, Allentoft ME, Sikora M, Rasmussen M, Gravel S, Guillen S, Nekhrizov G et al: Pulling out the 1%: whole-genome capture for the targeted enrichment of ancient DNA sequencing libraries. Am J Hum Genet 2013, 93(5):852-864.

21. Schuenemann VJ, Singh P, Mendum TA, Krause-Kyora B, Jager G, Bos KI, Herbig A, Economou

C, Benjak A, Busso P et al: Genome-wide comparison of medieval and modern Mycobacterium leprae. Science 2013, 341(6142):179-183.

22. Gansauge MT, Meyer M: Selective enrichment of damaged DNA molecules for ancient genome sequencing. Genome Res 2014, 24(9):1543-1549.

23. Avila-Arcos MC, Cappellini E, Romero-Navarro JA, Wales N, Moreno-Mayar JV, Rasmussen M, Fordyce SL, Montiel R, Vielle-Calzada JP, Willerslev E et al: Application and comparison of large-scale solution-based DNA capture-enrichment methods on ancient DNA. Sci Rep 2011, 1:74.

24. Cruz-Davalos DI, Llamas B, Gaunitz C, Fages A, Gamba C, Soubrier J, Librado P, Seguin-Orlando A, Pruvost M, Alfarhan AH et al: Experimental conditions improving in-solution target enrichment for ancient DNA. Molecular ecology resources 2016.

25. Orlando L, Ginolhac A, Raghavan M, Vilstrup J, Rasmussen M, Magnussen K, Steinmann KE, Kapranov P, Thompson JF, Zazula G et al: True single-molecule DNA sequencing of a pleistocene horse bone. Genome Res 2011, 21(10):1705-1719.

26. Ginolhac A, Vilstrup J, Stenderup J, Rasmussen M, Stiller M, Shapiro B, Zazula G, Froese D, Steinmann KE, Thompson JF et al: Improving the performance of true single molecule sequencing for ancient DNA. BMC Genomics 2012, 13:177.

27. Fierer N, Leff JW, Adams BJ, Nielsen UN, Bates ST, Lauber CL, Owens S, Gilbert JA, Wall DH, Caporaso JG: Cross-biome metagenomic analyses of soil microbial communities and their functional attributes. Proc Natl Acad Sci U S A 2012, 109(52):21390-21395.

28. Ding T, Schloss PD: Dynamics and associations of microbial community types across the human body. Nature 2014, 509(7500):357-360.

29. Wade WG: The oral microbiome in health and disease. Pharmacol Res 2013, 69(1):137-143.

30. Xu X, He J, Xue J, Wang Y, Li K, Zhang K, Guo Q, Liu X, Zhou Y, Cheng L et al: Oral cavity contains distinct niches with dynamic microbial communities. Environ Microbiol 2015, 17(3):699-710.

31. Ferretti P, Farina S, Cristofolini M, Girolomoni G, Tett A, Segata N: Experimental metagenomics and ribosomal profiling of the human skin microbiome. Experimental dermatology 2016.

32. O'Toole PW, Jeffery IB: Gut microbiota and aging. Science 2015, 350(6265):1214-1215.

33. Zhernakova A, Kurilshikov A, Bonder MJ, Tigchelaar EF, Schirmer M, Vatanen T, Mujagic Z, Vila AV, Falony G, Vieira-Silva S et al: Population-based metagenomics analysis reveals markers for gut microbiome composition and diversity. Science 2016, 352(6285):565-569.

34. Falony G, Joossens M, Vieira-Silva S, Wang J, Darzi Y, Faust K, Kurilshikov A, Bonder MJ, Valles-Colomer M, Vandeputte D et al: Population-level analysis of gut microbiome variation. Science 2016, 352(6285):560-564.

35. Donaldson GP, Lee SM, Mazmanian SK: Gut biogeography of the bacterial microbiota. Nat Rev Microbiol 2016, 14(1):20-32.

36. Rasmussen S, Allentoft ME, Nielsen K, Orlando L, Sikora M, Sjogren KG, Pedersen AG, Schubert M, Van Dam A, Kapel CM et al: Early divergent strains of Yersinia pestis in Eurasia 5,000 years ago.

Cell 2015, 163(3):571-582.

37. Maixner F, Krause-Kyora B, Turaev D, Herbig A, Hoopmann MR, Hallows JL, Kusebauch U, Vigl EE, Malfertheiner P, Megraud F et al: The 5300-year-old Helicobacter pylori genome of the Iceman. Science 2016, 351(6269):162-165.

38. Seifert L, Wiechmann I, Harbeck M, Thomas A, Grupe G, Projahn M, Scholz HC, Riehm JM: Genotyping Yersinia pestis in Historical Plague: Evidence for Long-Term Persistence of Y. pestis in Europe from the 14th to the 17th Century. PLoS One 2016, 11(1):e0145194.

39. Rollo F, Ermini L, Luciani S, Marota I, Olivieri C: Studies on the preservation of the intestinal microbiota's DNA in human mummies from cold environments. Med Secoli 2006, 18(3):725-740.

40. Ubaldi M, Luciani S, Marota I, Fornaciari G, Cano RJ, Rollo F: Sequence analysis of bacterial DNA in the colon of an Andean mummy. Am J Phys Anthropol 1998, 107(3):285-295.

41. Weyrich LS, Dobney K, Cooper A: Ancient DNA analysis of dental calculus. J Hum Evol 2015, 79:119-124.

42. Warinner C, Speller C, Collins MJ, Lewis CM, Jr.: Ancient human microbiomes. J Hum Evol 2015, 79:125-136.

43. Warinner C, Rodrigues JF, Vyas R, Trachsel C, Shved N, Grossmann J, Radini A, Hancock Y, Tito RY, Fiddyment S et al: Pathogens and host immunity in the ancient human oral cavity. Nat Genet 2014, 46(4):336-344.

44. Adler CJ, Dobney K, Weyrich LS, Kaidonis J, Walker AW, Haak W, Bradshaw CJ, Townsend G, Soltysiak A, Alt KW et al: Sequencing ancient calcified dental plaque shows changes in oral microbiota with dietary shifts of the Neolithic and Industrial revolutions. Nat Genet 2013, 45(4):450-455, 455e451.

45. Segata N, Izard J, Waldron L, Gevers D, Miropolsky L, Garrett WS, Huttenhower C: Metagenomic biomarker discovery and explanation. Genome Biol 2011, 12(6):R60.

46. Truong DT, Franzosa EA, Tickle TL, Scholz M, Weingart G, Pasolli E, Tett A, Huttenhower C, Segata N: MetaPhlAn2 for enhanced metagenomic taxonomic profiling. Nat Methods 2015, 12(10):902-903.

47. Pride DT, Salzman J, Haynes M, Rohwer F, Davis-Long C, White RA, 3rd, Loomer P, Armitage GC, Relman DA: Evidence of a robust resident bacteriophage population revealed through analysis of the human salivary virome. The ISME journal 2012, 6(5):915-926.

48. Willner D, Furlan M, Schmieder R, Grasis JA, Pride DT, Relman DA, Angly FE, McDole T, Mariella RP, Jr., Rohwer F et al: Metagenomic detection of phage-encoded platelet-binding factors in the human oral cavity. Proc Natl Acad Sci U S A 2011, 108 Suppl 1:4547-4553.

49. Metcalf JL, Xu ZZ, Weiss S, Lax S, Van Treuren W, Hyde ER, Song SJ, Amir A, Larsen P, Sangwan N et al: Microbial community assembly and metabolic function during mammalian corpse decomposition. Science 2016, 351(6269):158-162.

50. Tsuzukibashi O, Uchibori S, Shinozaki-Kuwahara N, Kobayashi T, Takada K, Hirasawa M: A selective medium for the isolation of Corynebacterium species in oral cavities. J Microbiol Methods 2014, 104:67-71.

51. Colombo AP, Boches SK, Cotton SL, Goodson JM, Kent R, Haffajee AD, Socransky SS, Hasturk

H, Van Dyke TE, Dewhirst F et al: Comparisons of subgingival microbial profiles of refractory periodontitis, severe periodontitis, and periodontal health using the human oral microbe identification microarray. J Periodontol 2009, 80(9):1421-1432.

52. Shaddox LM, Huang H, Lin T, Hou W, Harrison PL, Aukhil I, Walker CB, Klepac-Ceraj V, Paster BJ: Microbiological characterization in children with aggressive periodontitis. J Dent Res 2012, 91(10):927-933.

53. Antunes HS, Rocas IN, Alves FR, Siqueira JF, Jr.: Total and Specific Bacterial Levels in the Apical Root Canal System of Teeth with Post-treatment Apical Periodontitis. J Endod 2015, 41(7):1037-1042.

54. Javed S, Said F, Eqani SA, Bokhari H: Bordetella parapertussis outbreak in Bisham, Pakistan in 2009-2010: fallout of the 9/11 syndrome. Epidemiol Infect 2015, 143(12):2619-2623.

55. Vidor C, Awad M, Lyras D: Antibiotic resistance, virulence factors and genetics of Clostridium sordellii. Res Microbiol 2015, 166(4):368-374.

56. Hanif H, Anjum A, Ali N, Jamal A, Imran M, Ahmad B, Ali MI: Isolation and Antibiogram of Clostridium tetani from Clinically Diagnosed Tetanus Patients. Am J Trop Med Hyg 2015, 93(4):752-756.

57. Endersen L, Coffey A, Ross RP, McAuliffe O, Hill C, O'Mahony J: Characterisation of the antibacterial properties of a bacterial derived peptidoglycan hydrolase (LysCs4), active against C. sakazakii and other Gram-negative food-related pathogens. International journal of food microbiology 2015, 215:79-85.

58. Human Microbiome Project C: Structure, function and diversity of the healthy human microbiome. Nature 2012, 486(7402):207-214.

59. Jonsson H, Ginolhac A, Schubert M, Johnson PL, Orlando L: mapDamage2.0: fast approximate Bayesian estimates of ancient DNA damage parameters. Bioinformatics 2013, 29(13):1682-1684.

60. Briggs AW, Stenzel U, Johnson PL, Green RE, Kelso J, Prufer K, Meyer M, Krause J, Ronan MT, Lachmann M et al: Patterns of damage in genomic DNA sequences from a Neandertal. Proc Natl Acad Sci U S A 2007, 104(37):14616-14621.

61. Sawyer S, Krause J, Guschanski K, Savolainen V, Paabo S: Temporal patterns of nucleotide misincorporations and DNA fragmentation in ancient DNA. PLoS One 2012, 7(3):e34131.

62. Willerslev E, Hansen AJ, Ronn R, Brand TB, Barnes I, Wiuf C, Gilichinsky D, Mitchell D, Cooper A: Long-term persistence of bacterial DNA. Curr Biol 2004, 14(1):R9-10.

63. Schubert M, Ermini L, Der Sarkissian C, Jonsson H, Ginolhac A, Schaefer R, Martin MD, Fernandez R, Kircher M, McCue M et al: Characterization of ancient and modern genomes by SNP detection and phylogenomic and metagenomic analysis using PALEOMIX. Nat Protoc 2014, 9(5):1056-1082.

64. DeSantis TZ, Hugenholtz P, Larsen N, Rojas M, Brodie EL, Keller K, Huber T, Dalevi D, Hu P, Andersen GL: Greengenes, a chimera-checked 16S rRNA gene database and workbench compatible with ARB. Appl Environ Microbiol 2006, 72(7):5069-5072.

65. Wang Q, Garrity GM, Tiedje JM, Cole JR: Naive Bayesian classifier for rapid assignment of rRNA

sequences into the new bacterial taxonomy. Appl Environ Microbiol 2007, 73(16):5261-5267.

66. Setlow P: Mechanisms for the prevention of damage to DNA in spores of Bacillus species. Annu Rev Microbiol 1995, 49:29-54.

67. Bouwman AS, Kennedy SL, Muller R, Stephens RH, Holst M, Caffell AC, Roberts CA, Brown TA: Genotype of a historic strain of Mycobacterium tuberculosis. Proc Natl Acad Sci U S A 2012, 109(45):18511-18516.

68. Weyrich LS, Rolin OY, Muse SJ, Park J, Spidale N, Kennett MJ, Hester SE, Chen C, Dudley EG, Harvill ET: A Type VI secretion system encoding locus is required for Bordetella bronchiseptica immunomodulation and persistence in vivo. PLoS One 2012, 7(10):e45892.

69. Bendor L, Weyrich LS, Linz B, Rolin OY, Taylor DL, Goodfield LL, Smallridge WE, Kennett MJ, Harvill ET: Type Six Secretion System of Bordetella bronchiseptica and Adaptive Immune Components Limit Intracellular Survival During Infection. PLoS One 2015, 10(10):e0140743.

70. Juras A, Chylenski M, Krenz-Niedbala M, Malmstrom H, Ehler E, Pospieszny L, Lukasik S, Bednarczyk J, Piontek J, Jakobsson M et al: Investigating kinship of Neolithic post-LBK human remains from Krusza Zamkowa, Poland using ancient DNA. Forensic science international Genetics 2017, 26:30-39.

71. Yang DY, Eng B, Waye JS, Dudar JC, Saunders SR: Technical note: improved DNA extraction from ancient bones using silica-based spin columns. Am J Phys Anthropol 1998, 105(4):539-543.

72. Malmstrom H, Svensson EM, Gilbert MT, Willerslev E, Gotherstrom A, Holmlund G: More on contamination: the use of asymmetric molecular behavior to identify authentic ancient human DNA. Mol Biol Evol 2007, 24(4):998-1004.

73. Meyer M, Kircher M: Illumina sequencing library preparation for highly multiplexed target capture and sequencing. Cold Spring Harbor protocols 2010, 2010(6):pdb prot5448.

74. Schubert M, Lindgreen S, Orlando L: AdapterRemoval v2: rapid adapter trimming, identification, and read merging. BMC Res Notes 2016, 9:88.

75. Langmead B, Salzberg SL: Fast gapped-read alignment with Bowtie 2. Nat Methods 2012, 9(4):357-359.

76. Meyer LR, Zweig AS, Hinrichs AS, Karolchik D, Kuhn RM, Wong M, Sloan CA, Rosenbloom KR, Roe G, Rhead B et al: The UCSC Genome Browser database: extensions and updates 2013. Nucleic Acids Res 2013, 41(Database issue):D64-69.

77. Andrews RM, Kubacka I, Chinnery PF, Lightowlers RN, Turnbull DM, Howell N: Reanalysis and revision of the Cambridge reference sequence for human mitochondrial DNA. Nat Genet 1999, 23(2):147.

TABLE AND FIGURE LEGENDS

Table 1. Characteristics of the samples extracted from ancient human remains.

Archaeological site	ID	Sample no.	Sample no. that passed selection	Dating	Date of excavation	Storage conditions	Sample type
**Roman Age Group**							
Kowalewko	KO	58	48	100AD-300AD	1990s	Long deposit	Tooth
Masłomęcz	MZ	27	24	200AD-400AD	1970-1990	Long deposit	Tooth
**Medieval Group**							
Sowinki	SI	21	19	1000AD-1100AD	1980s	Long deposit	Tooth
Niemcza	NA	36	31	900AD-1000AD	1960s	Long deposit	Tooth
Markowice	ME	8	8	1000AD-1200AD	2014	Arch. site	Tooth
Gniezno	GO	2	2	1000AD-1200AD		Long deposit	Tooth
Łęgowo	LO	9	8	1000AD-1200AD	2013-2015	Short deposit	Tooth

Table 2. The percentage of bacteria/archaea of a given respiratory type [(facultative) aerobic/anaerobic] and gram stain type [positive/negative] within environmental and human-related groups [oral, pathogenic, or other].

Group	(Facultative) anaerobic	(Facultative) aerobic	Gram-positive	Gram-negative
Environmental	4%	96%	66%	34%
Oral	76%	24%	45%	55%
Pathogenic	51%	49%	32%	68%
Other human-related	46%	54%	70%	30%

Figure 1. A) The geographical positions of archaeological sites. KO and MZ are from the Roman Age group, and SI, NA, ME, GO, LO are from the Medieval Group. Samples from ME were collected directly at the archaeological site. B) Number of filtered reads (y-axis) per archaeological site (x-axis). C) Percentage of reads mapped to the human genome (y-axis) per archaeological site (x-axis).

Figure 2. Microorganism kingdoms detected in analyzed archaeological samples. A) Pie-plot representing overall frequency of microorganism kingdoms in archaeological samples; B) box and whiskers plot representing the distribution of frequencies of particular microorganism kingdoms in archaeological sites (GO not shown as includes only 2 samples); and C) stacked barplot indicating the frequency of microorganism kingdoms in a particular sample. Each bar represents an individual sample. Samples are ordered by the archeological sites. The color legend for all plots is shown at the bottom.

Figure 3. Bacterial and archaeal classes detected in analyzed archaeological samples. A) Pie-plot representing overall frequency of bacterial and archaeal classes in archaeological samples; B) box and whiskers plot representing distribution of frequencies of 6 most abundant bacterial classes (present in at least 1%) in archaeological sites (GO not shown as includes only 2 samples); and C) stacked barplot indicating the frequency of bacterial and archaeal classes in a particular sample. Each stacked bar represents an individual sample. Samples are ordered by the archeological sites. The color legend for all plots is shown at the bottom.

Figure 4. Bacterial and archaeal types [environmental (light green), oral (blue), other (yellow) and pathogenic (red)] detected in analyzed archaeological samples. A) Pie-plot representing overall frequency of bacterial and archaeal types in archaeological samples; B) box and whiskers plot representing distribution of frequencies of bacterial and archaeal types in archaeological sites (GO not shown as includes only 2 samples); and C) stacked barplot indicating the frequency of bacterial and archaeal types in a particular sample. Each stacked bar represents an individual sample. Samples are ordered by the archeological sites. The color legend for all plots is shown at the bottom.

Figure 5. Principal coordinate analysis of microbial compositions at four taxonomic levels: A) class, B) family, C) genus, and D) species. Samples from certain archaeological sites are marked in different colors and labeled with archaeological site ID.

Figure 6. A) Comparison of bacterial and archaeal profiles (stacked barplot) on class level based on shallow and deep sequencing of the selected 11 samples (Sample ID is indicated on the x-axis, the first

bar in a pair is shallow and the second is deep sequencing). The correlation coefficient R is placed above each shallow/deep stacked bar pair. The color legend is the same as in Figure 3. B) Correlation R values (y-axies) for shallow and deep sequencing pairs on different taxonomic levels (C – class, F – family, G –genus, S – species).

Figure 7. The DNA damage in samples with different fractions of environmental bacteria/archaea. Barplots indicating deamination rate in single stranded DNA overhangs (δ_s) and double stranded DNA fragments (δ_d) in microbial (left-hand site) and human DNA (right-hand site), grouped based on the fraction of environmental bacteria/archaea in sample and the length of single-stranded DNA overhangs (λ , expressed as:1/λ-1) calculated for 77 representative bacteria/archaea and endogenous human aDNA. Samples were grouped based on the fraction of environmental bacteria/archaea in a sample (0–25%, 25–50%, 50–75% and 75–100%).

Figure 8. The differences of DNA damage levels [$\Delta\delta_s$, $\Delta\delta_d$, Δλ , expressed as: Δ(1/λ-1)] of bacteria/archaea species belonging to the 5 groups (environmental, all human-related, oral, gut and pathogen) in comparison to damage levels in human aDNA. Boxes, whiskers and dots represent the distribution of differences in DNA damage levels of particular bacterial/archaeal groups. Each dot represents the difference in an individual sample. The color legend is the same as in Figure 4 (All human-related species are in orange).

Figure 9. DNA damage level (δ_s, δ_d, 1/λ-1) in environmental and all human-related *Actinobacteria* species. Boxes, whiskers and dots represent the distribution of DNA damage levels in particular samples. The color legend is the same as in Figure 4 (All human-related species are in orange).

Figure 1

Click here to download Figure Figure_1.png

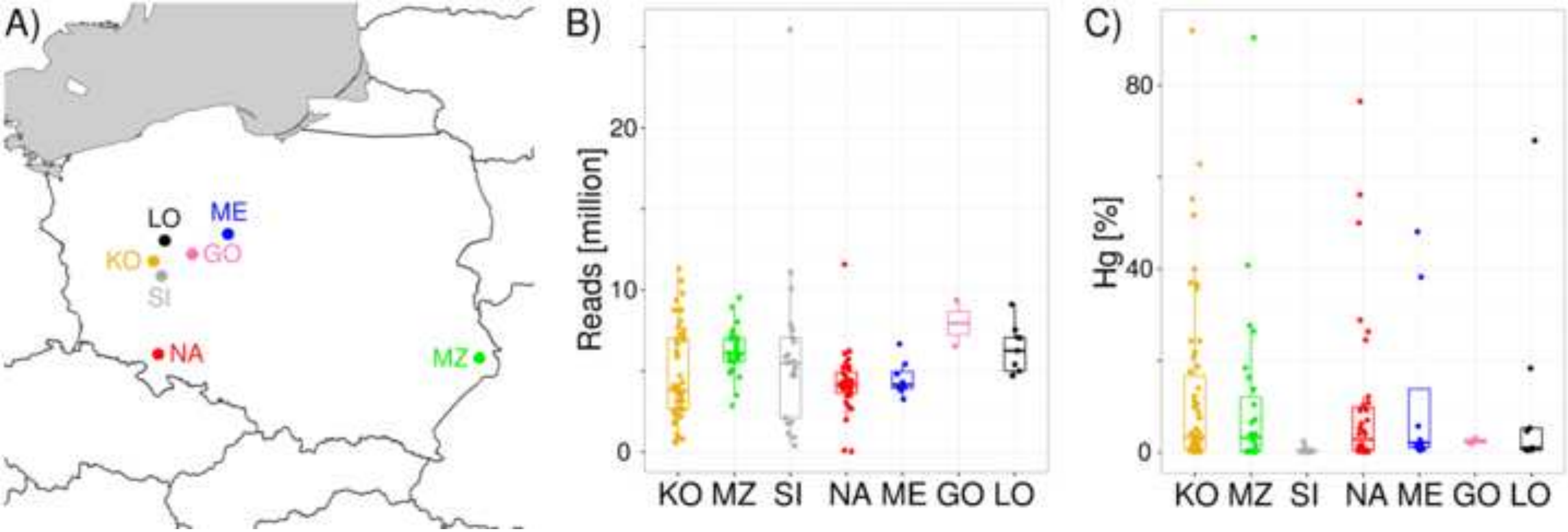

Figure 2

Click here to download Figure Figure_2.png

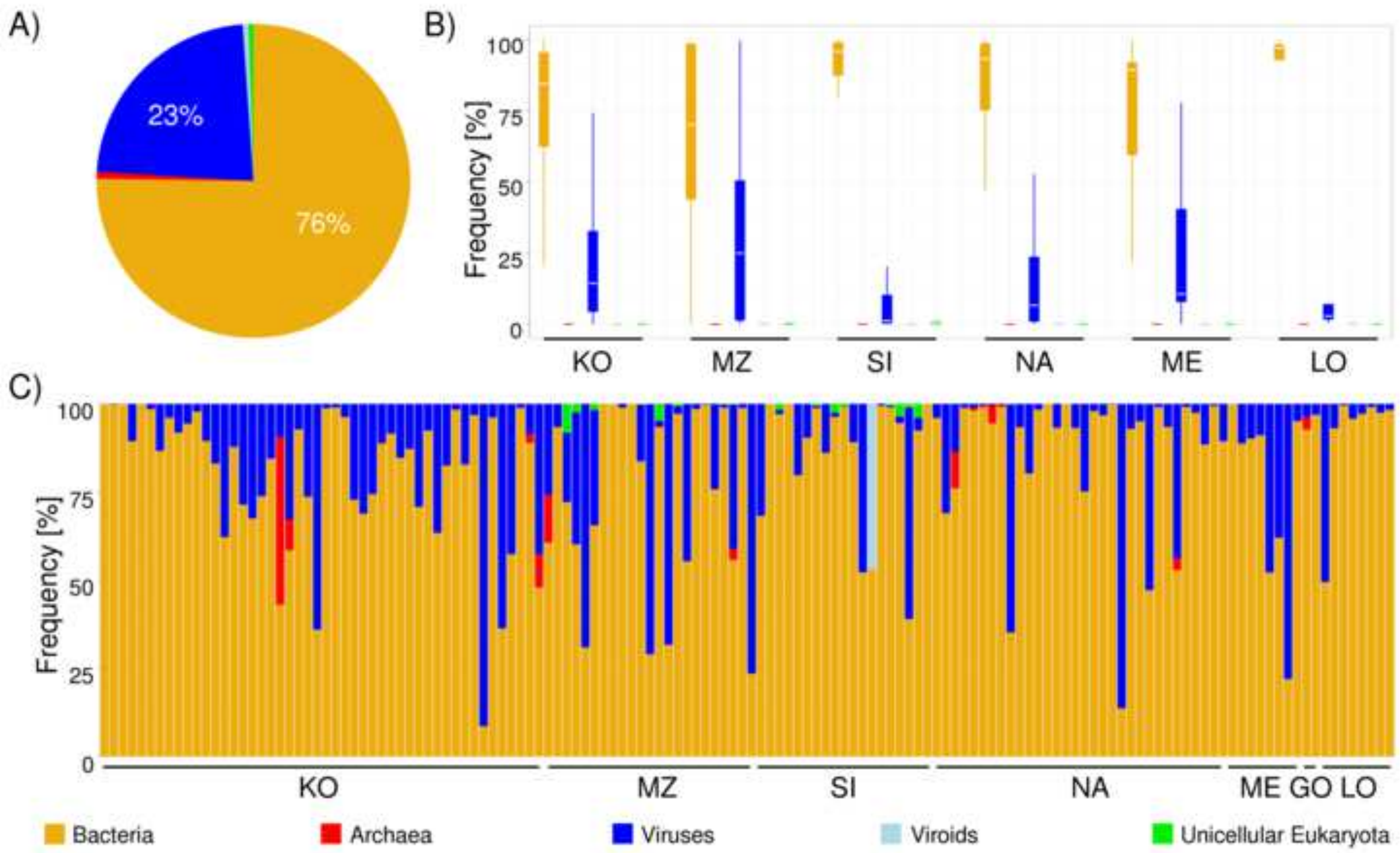

Figure 3

Click here to download Figure Figure_3.png

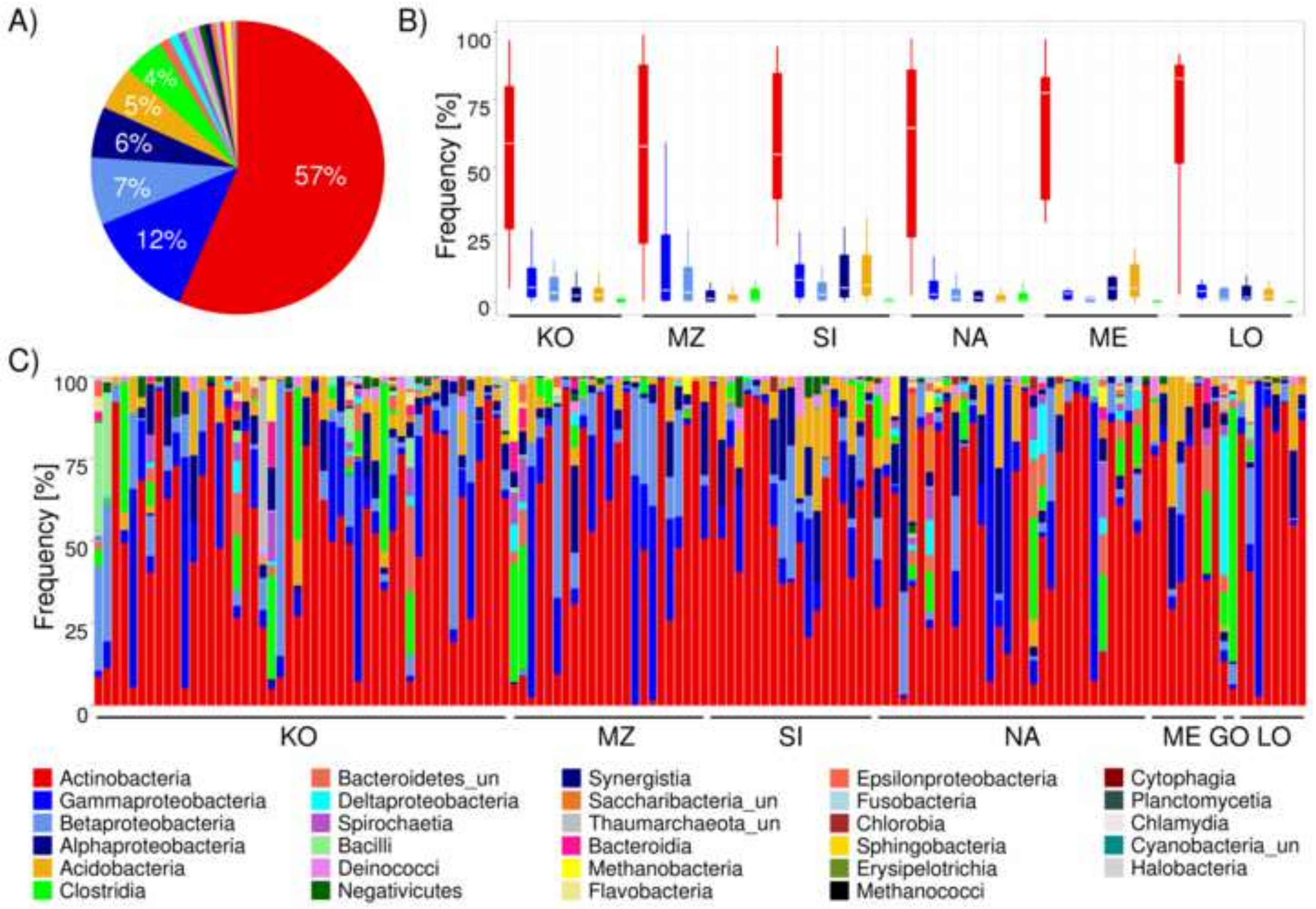

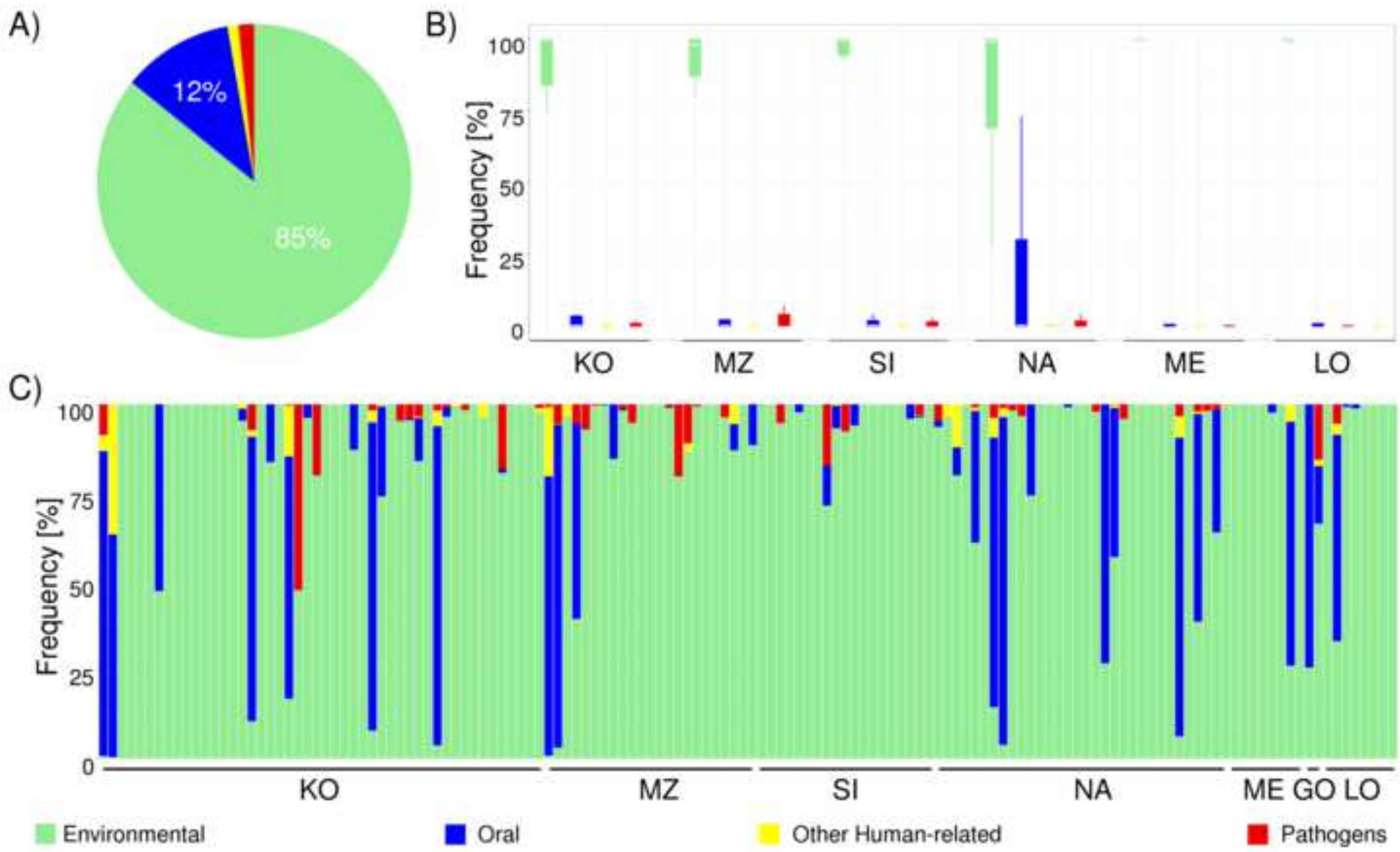
A)
12%
85%
B)
Frequency [%]
100
75
50
25
0
KO
MZ
SI
NA
ME
LO
C)
Frequency [%]
100
75
50
25
0
KO
MZ
SI
NA
ME GO LO
Environmental
Oral
Other Human-related
Pathogens

Figure 5

Click here to download Figure Figure_5.png

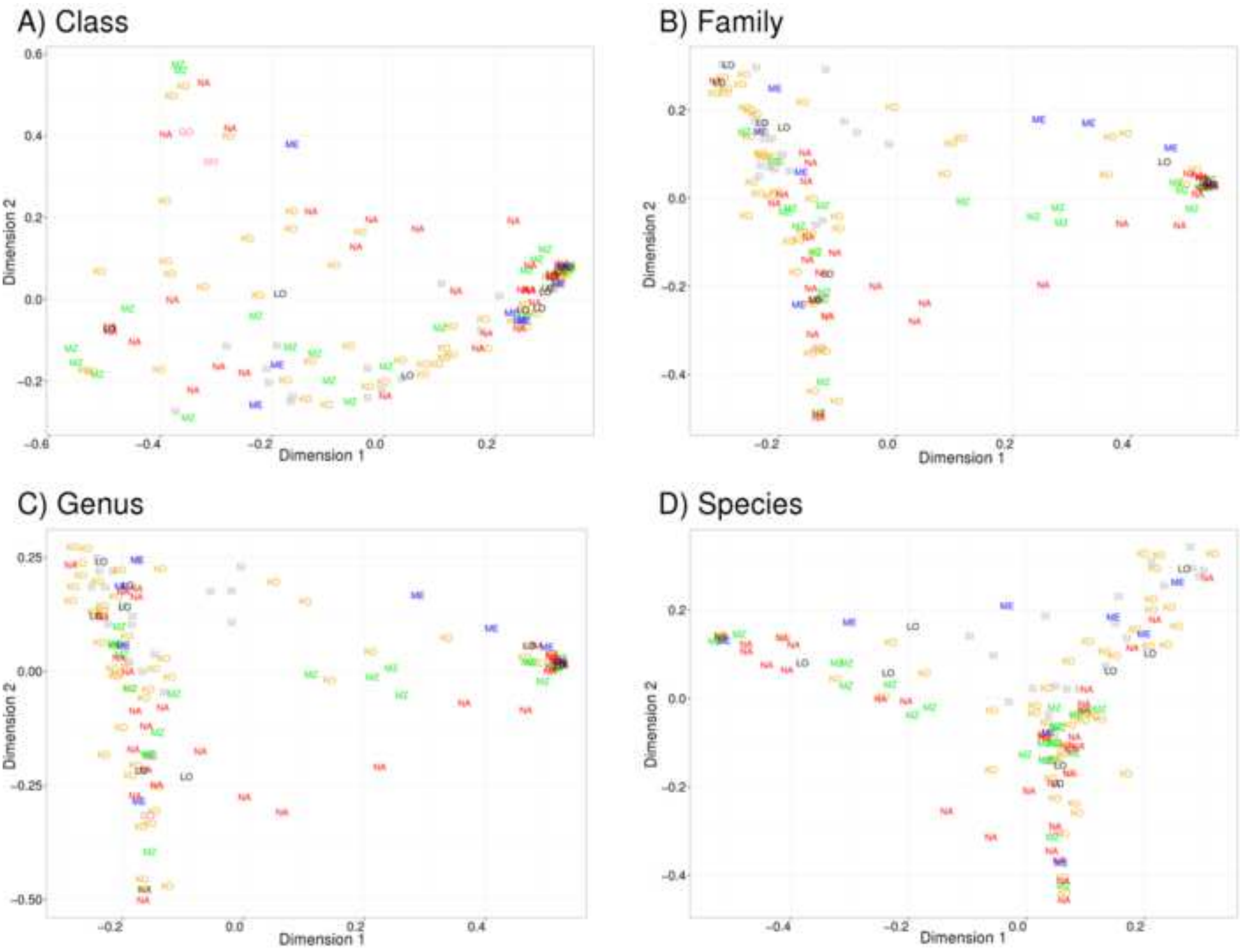

Figure 6

Click here to download Figure Figure_6.png

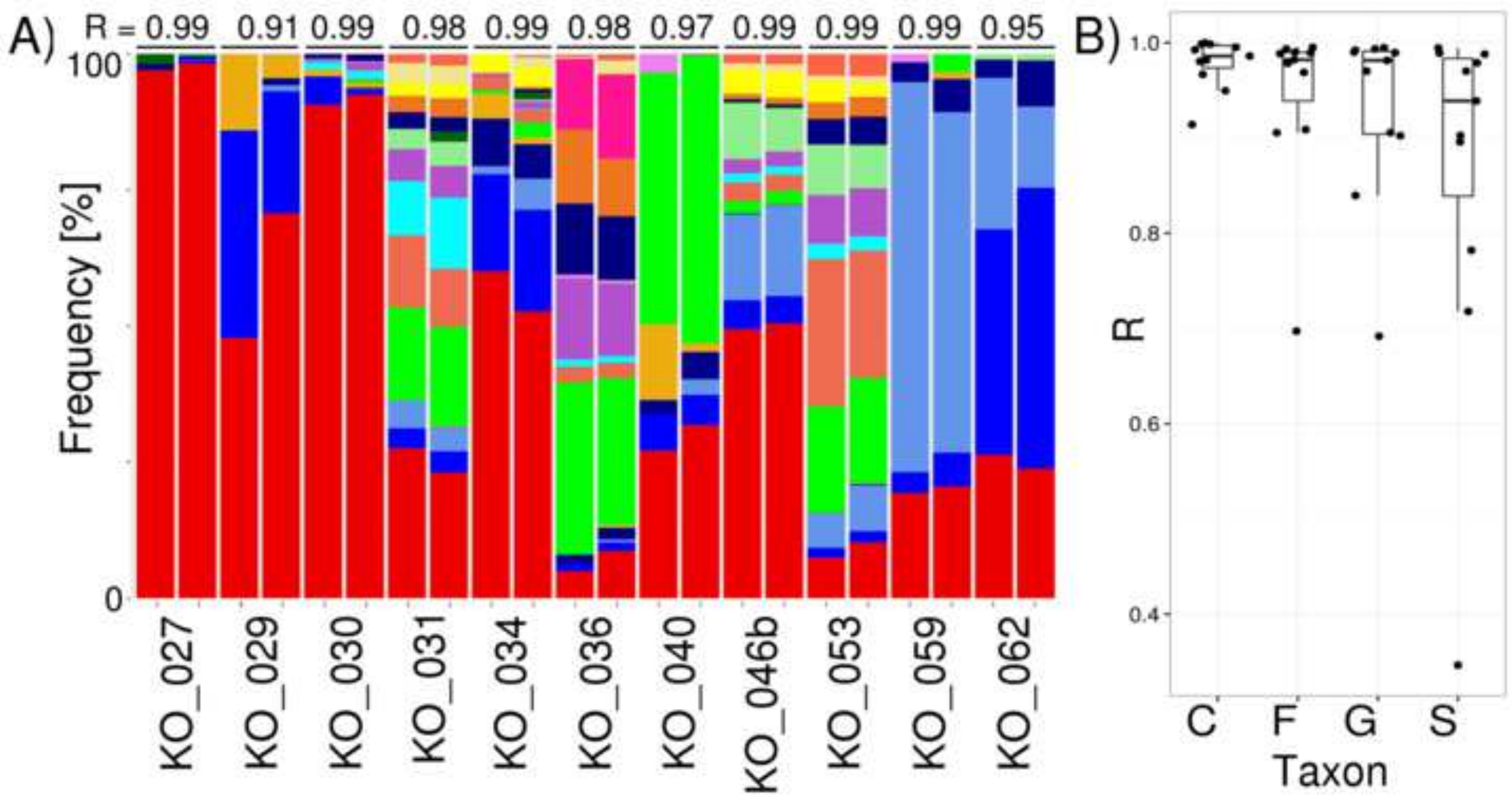

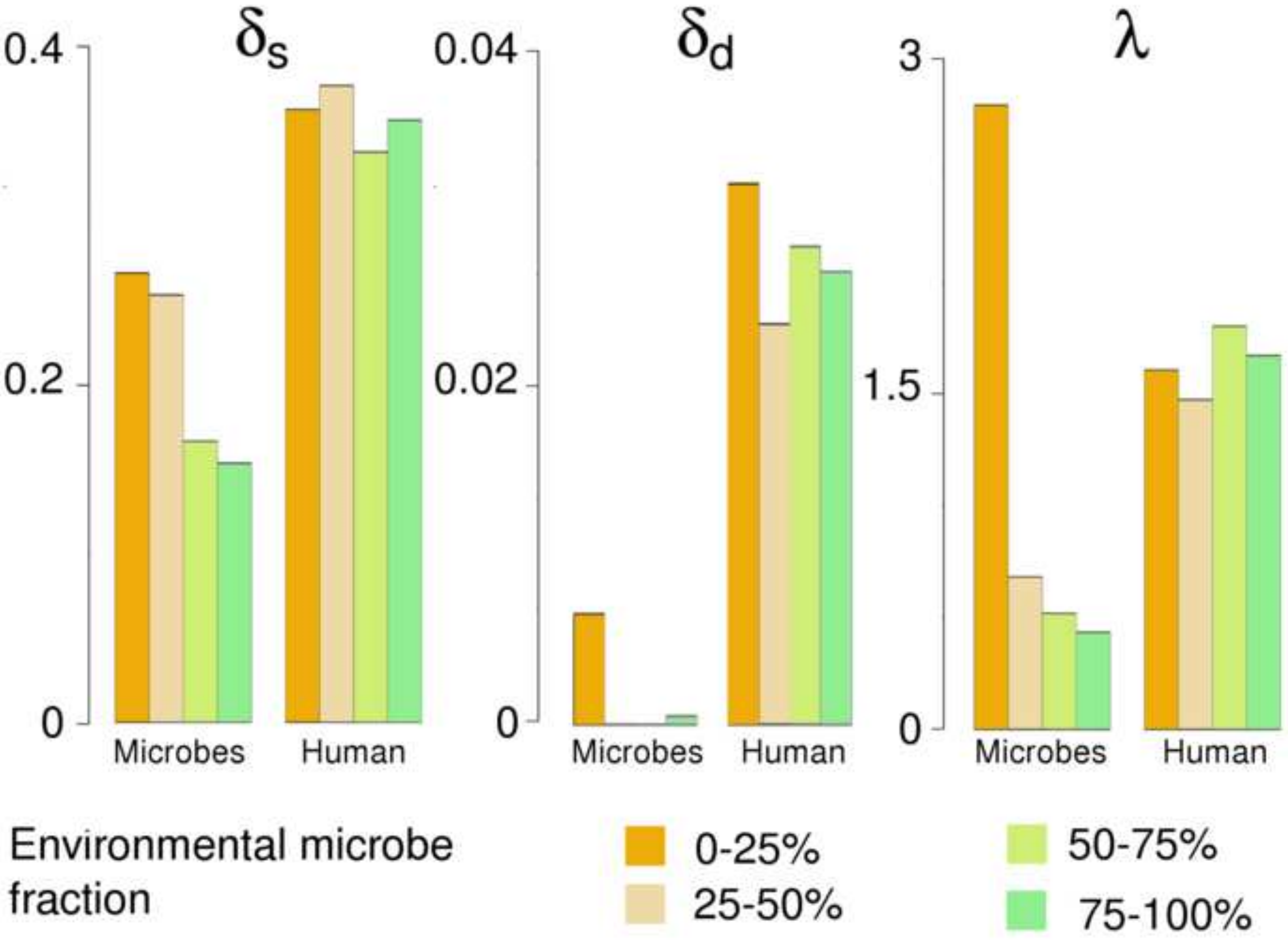

δ_s
0.4
0.2
0
Microbes
Human
δ_d
0.04
0.02
0
Microbes
Human
λ
3
1.5
0
Microbes
Human
Environmental microbe fraction
0-25%
25-50%
50-75%
75-100%

Figure 8

Click here to download Figure Figure_8.png

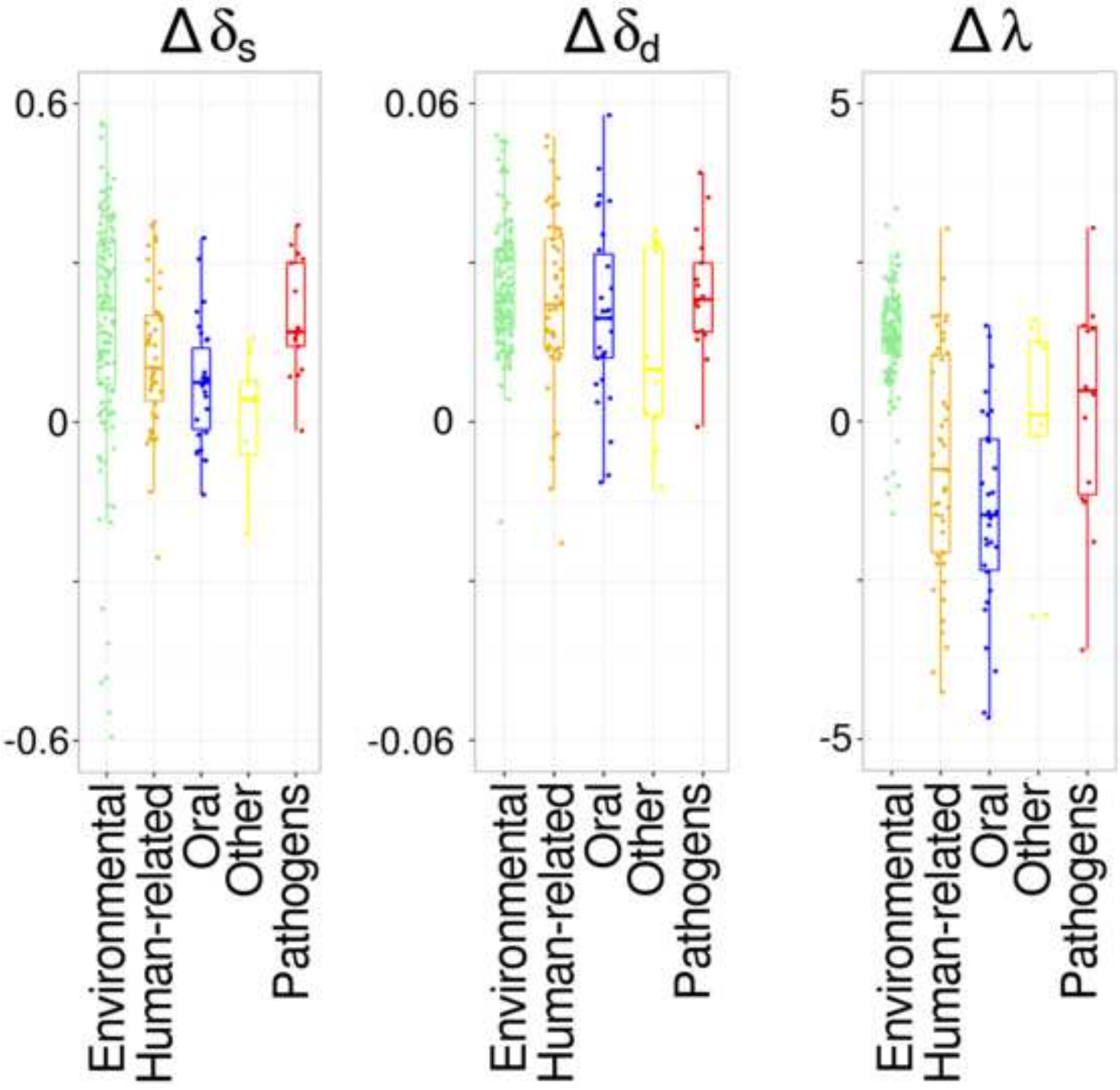

Figure 9

Click here to download Figure Figure_9.png

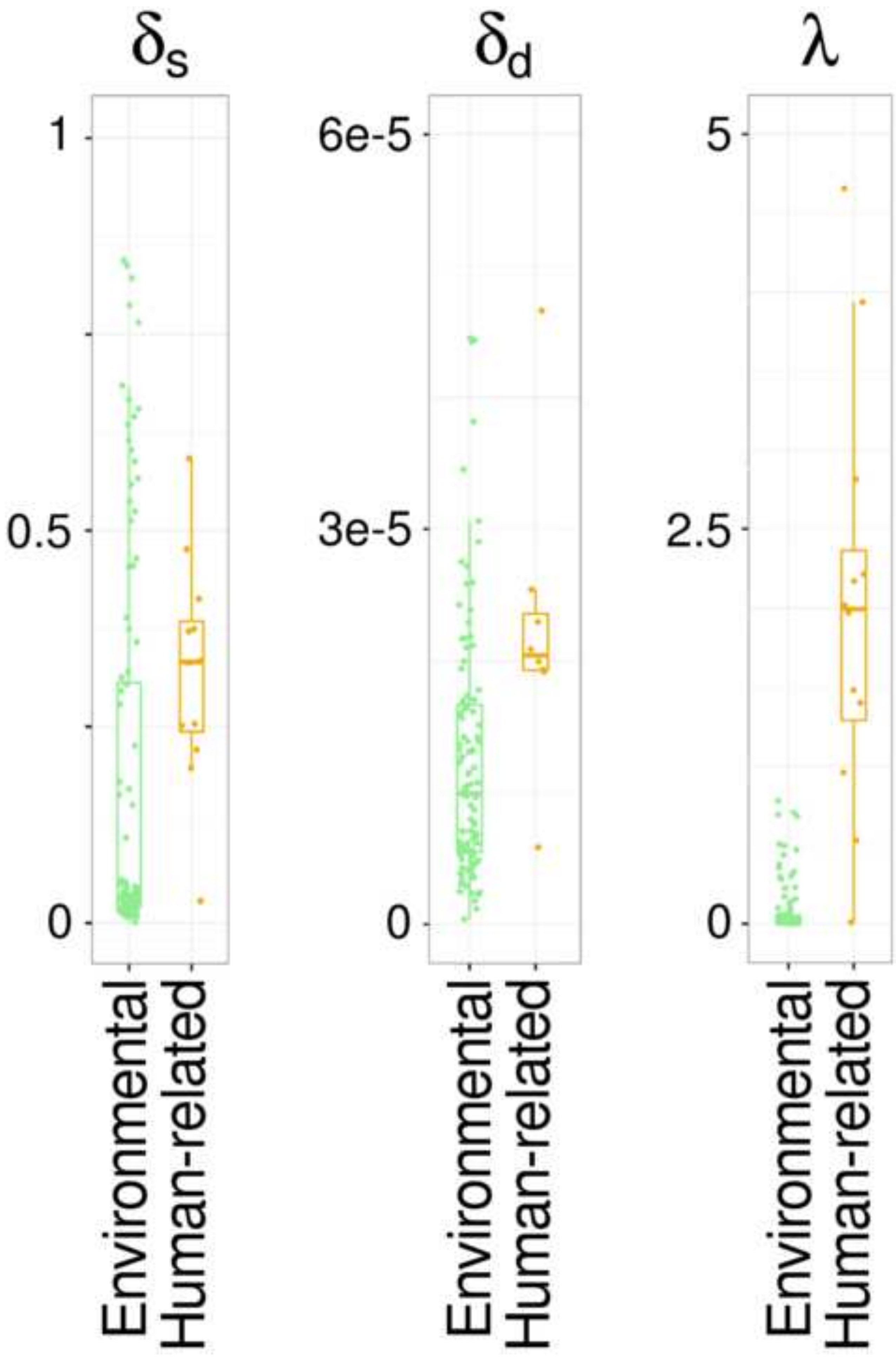

Supplementary Figures

Click here to access/download
Supplementary Material
aDNA_microorganisms_Figlerowicz_Supplementary Figures.pdf

Supplementary Tables

Click here to access/download
Supplementary Material
aDNA_microorganisms_Figlerowicz_Supplementary_Tables.xlsx

Supplementary Tables Legends

Click here to access/download
Supplementary Material
aDNA_microorganisms_Figlerowicz_Supplementary_Tables_leg.docx

 Click here to download Personal Cover aDNA_microorganisms_Figlerowicz_Reviews_Answers.docx

Dear Editor,

On behalf of all the authors, I would like to thank you for giving us an opportunity to re-submit the manuscript, entitled, *Comprehensive analysis of microorganisms accompanying human archaeological remains* for publication in *GigaScience*, possibly within *Functional Metagenomics (Meta-Func)* series.

As suggested, we responded positively to all of the Reviewers comments which we found very helpful and of great importance. The introduction of advised changes and analyses added a huge value to our manuscript. Following the Reviewers comments we also introduced some changes to the study goals, conclusions and results interpretation. We revised the manuscript and made sure to state clearly that we intended to characterize microbiome of aDNA samples – in particular, all bacteria present there (modern contaminants as well as potentially ancient species).

The biggest concern of the Reviewer 2 was the lack of negative controls. In fact, we do follow standards set by the world's leading experts in aDNA field, as prof. Orlando and prof. Cooper, so we did all necessary controls conscientiously as a part of standard aDNA extraction protocol. We added additional paragraph in the Methods section entitled "Contamination control" and are ashamed that it was not done in the first submission.

Below I am attaching all the remaining answers to the Reviewers. I hope that the answers and a revised manuscript will satisfy both of them and a publication in *GigaScience* will be possible.

Reviewer 1

Philips and colleagues have performed an extensive comparative analysis of the microbial communities in human teeth, sampled from different ages and locations across Central Europe. They show there is a huge variation in the microbial profiles of different samples, regardless of their origin or age. Additionally, they remarkably identify some ancient human-related bacteria not associated with post-mortem contamination. Overall, I found the paper interesting, although I have some comments for its improvement.

- Major concerns/questions to be clarified:

1) What part of the teeth did the authors sample? It has been shown that dentine has more endogenous DNA content, while the cementum is richer in microbial communities. Did the authors account for the different preservation of the teeth structure, and sampled always from the same part? Were the enamel or the cementum fully preserved in all cases? This needs to be clearly stated in the main.

According to the Reviewers suggestion we added missing information in the revised manuscript (see "DATA DESCRIPTION", page 5 line 113)

2) Authors claim that "The average number of reads did not differ substantially between archaeological sites or type of storage (Figure 1 B).", and "No evident differences in the amount of human aDNA content were observed for older (KO, MZ) and younger (SI, NA, ME, GO, LO) sample sets as well as for freshly recovered and stored in museum samples.". It might be true, but it visually seems that the median Hg is higher for KO and MZ. Or at least that KO and MZ have a higher fraction of samples with a high Hg. Can they provide a p-value supporting their statements? In general, also in other parts of the main, their statements are not accompanied by the name of the statistical test applied, and the corresponding p-value.

As suggested, we went through the main text and added information on the statistical tests applied, and corresponding p-values. (see: page 5 lines: 122, 128,129,130; page 6 lines: 145,146; page 7, lines: 179,180,182,183,196; page 9 lines: 230,231; page 11 lines: 289,290).

3) They speculate that viruses come from plants, and were acquired from the environment. Is this acquisition post-mortem contamination, or it reflects diet preferences? Do these reads mapping to viruses show signs of post-mortem damage typical of ancient DNA? It would be interesting to see to what extent human diet can be traced through ancient metagenome analyses.

We found the Reviewers question very interesting and must admit that we already tried to do such analyses before. Unfortunately, we found a couple of limitations which made the results too unreliable and incomplete to be published. The main concern was: very low number of reads mapping to the viral genomes (median=360). MapDamage could not asses the DNA damage level properly, what was clearly visible on a graphs generated [data not shown].
What's more, RNA viruses are not expected to preserve in the archaeological fossils in a high number (see Gilbert 2013 "Deep Sequencing of RNA from Ancient Maize Kernels") and DNA-seq library preparation procedure exclude RNA from being sequenced. For that reasons, it would be impossible to investigate the overall profile of viruses.
Lastly, viruses may have single or double stranded genomes. We applied double stranded NGS protocol, so single stranded viruses were not sequenced at all. Moreover, a ssDNA viruses damage pattern would be different from that observed in dsDNA and would require prior standardizing experiments and model generation, as performed before for dsDNA (see Briggs AW at all. "Patterns of damage in genomic DNA sequences from a Neandertal" PNAS 2007).
In general, we found viruses subject very interesting and worth further study, but to investigate them a proper experiments should be applied, possibly within another project.

4) Historically speaking, was the diet of these human populations comparable at all? Their diet likely influenced their composition in human-related oral/gut microbiota. In a PCoA analysis or similar, do the "oral" microbiota separate these human populations?

We ran recommend PCA as suggested by the Reviewer (Supplementary Figure 4). As it is shown in Supplementary Figure 4 oral microbiota did not separate archaeological sites (nor environmental ones). However it has to be admitted that, our analysis might be underpowered to detect the suggested effect. Generally, we agree with the Reviewers assumption and we believe that experiment dedicated for analysis

of ancient oral flora, although difficult to perform as of present, would confirm the hypothesis (see Adler CJ at all "Sequencing ancient calcified dental plaque shows changes in oral microbiota with dietary shifts of the Neolithic and Industrial revolutions.", Nat Gen 2013").

5) Are the samples with more human-related microbiota the ones with a higher fraction of plant-infecting viruses? For example, Clostridia appear as very frequent in some KO outlier samples (fig 3C), although those outliers are probably not plot in Fig. 3b. If these individuals are the same having a higher fraction of plant-infecting viruses, it could reflect a better preservation of the microbiota. If so, are these KO samples the ones with the higher/lower endogenous content (KO shows a bimodal distribution in terms of Hg)?

Based on the information in Supplementary Table 1 we examined the correlation between viruses % and All-human-related bacteria %, as well as between hg % and All-human-related bacteria % and found no correlation. We added this information in the main text (page 7/8 lines: 199-201).

6) It is not surprising that ancient oral microbiota is enriched in Gram-negative bacteria, since lysozyme preferentially protects us against Gram-positive bacteria. I do not think this over-representation would be evident in other human bones. This should be clearly specified to avoid misleading interpretations from the readers.

We thank the Reviewer for this remark - an appropriate comment and the reference in the main text has been added (page 8 lines:216,217).

7) Can the authors show other dimensions in the PCoA? What fraction of the variance is explained by, let´s say, the three first dimensions?

As suggested, we added third dimension and information about the fraction of variance explained by each dimension to the supplementary material PCA graphs (see Supplementary Figure 3, Supplementary Figure 4).

8) I suggest to perform a hierarchical clustering restricted to the microbial profile of "environmental" bacteria. And a hierarchical clustering based on "oral" and "human-related" bacteria. I ask so because human-related bacteria can be site-specific, depending on diet, diseases, etc, and thus blur out a temporal (age) or preservation (time in the museum) effect. By separating the clustering into these two components, some clearer structure might emerge. At least it is worth to try.

We performed hierarchical clustering restricted to "environmental" and "human-related" genera. The results confirmed corresponding PCA results (Supplementary Figure 4) – samples were not segregated by archeological sites (nor age, storage type), see Supplementary Figure 5. See also answer to Point 4.

9) The authors claim that shallow sequencing is enough to have an accurate idea of the microbial profile at the clade level. I am not sure if we could say the same at the species level, because the higher the number of covered markers, the higher the taxonomic resolution. If the composition changes more at the species

level, the authors should rephrase the conclusion for this section, as deeper sequencing would provide higher resolution. The authors can perhaps provide some guidelines for future sequencing efforts, relative to the aimed taxonomic resolution.

We agree with the Reviewer and are aware of limitations of shallow sequencing which increase with the depth of taxonomic level. Inspired by the Reviewers comment we analyzed shallow vs deep sequencing results not only on a class, but also on a family, genus and species levels and added the results to the manuscript (see Supplementary Figure 7). In agreement with the Reviewer assumption, the results confirmed expected decrease of correlation values with the depth of taxonomic level (average R: 0.96, 0.90, 0.88, 0.78 for class, family, genus and species level, respectively; see Figure 6B). Although the correlation decreased with the depth of taxonomic level it was still, in most cases, high and very high and highly significant ($p<0.0001$, Supplementary Figure 7) and therefore the results supported our general conclusion that shallow sequencing may be successfully used for preliminary microbiome characterization.

-Minor issue:
GO is not listed as Medieval in the following sentence: "As shown in Table 1, the samples differed by age (Roman Age group (KO and MZ) or Medieval group (SI, NA, ME, and LO)) and by storage conditions (specimens that were in museum deposits for at least 20 years (long deposit: KO, MZ, SI, NA, and GO), relatively freshly discovered specimens (stored in museum deposit <5 years, short deposit: LO) or samples taken directly from an archaeological site (Arch. site: ME))"

We added “GO” in the Medieval group.

Reviewer 2

Philips et al. sequence DNA obtained from 161 ancient teeth samples using NGS technology. The goal of the study was to examine the microbiome (oral and environmental) in the teeth, and try to identify pathogens that could be preserved within the teeth. While this study is timely and interesting for the ancient DNA field, several technical limitations and inappropriate analyses - the largest being the lack of negative controls and the assumption that bacterial families can be classified as human or environmental - limit this data from being published in its current form.

We thank the Reviewer for the comments which we found very valuable and inspiring and we spare no effort to meet the Reviewer expectations.
Yet, we would like to emphasize that the goal of our study was to characterize all microbes - ancient as well as modern (presumably mostly modern bacteria that colonized or contaminated bones recently) that accompanied ancient samples. We are sorry for unintended introduction of some misleading statements or over interpretation of some results that might give a false impression that we intended to study ancient oral/gut microbiome. To clarified this issue we introduced a necessary changed in the main text (page 2 lines:28-29, 36,38,40-41; page 3 lines: 58-60, 65-66; page: 8 lines:200-201; page 9 lines: 237-239; page 11 lines:314-316).

Major Points:

1.) While exploring the bacterial background of these samples is interesting, this can only be done if negative controls were collected and processed simultaneously with the samples. Extraction blank controls are standard in the field of ancient DNA research, and must be included and sequenced to identify and explain the modern bacteria that were introduced into the DNA extraction, library preparation, and sequencing process.

The Reviewer is right, negative controls are a part of standard aDNA extraction protocol which we routinely use in all experiments. We added lacking information about blank controls made in a new paragraph “Contamination control”, as well as we extend “Experimental procedures” where we described all of the decontamination procedure we applied (page 14/15 lines: 393-416).

Minimally, environmental samples from each of these sites would have needed to be included, i.e. soil or swabs from non-oral bones from each of the sites. Without these controls, the readers are unable to determine if any of the identified bacterial species are indeed ancient, or are simply the result of bleach damage in the ancient lab during processing, even if damage profiles are assessed for each species. This is a major area of concern, and without these controls, the data simply cannot be published as is. Several publications from the Orlando group in Copenhagen have demonstrated that all ancient bones contain a varied microbial signal (regardless if they were teeth or not), and a publication by Salter et al. 2015 describes the large amounts of microbial contamination that are introduced into even modern samples from 'clean' lab reagents and environments.

First of all we modified the manuscript to remove misleading statements and clarify that we do not claim that environmental bacteria are ancient. Our results show the opposite - that most or all, environmental bacteria are relatively fresh and most likely are contaminant of surrounding environment.
In fact, in our studies we followed contamination control standards set by Orlando group (e.g. see the most relevant work: Der Sarkissian C, Ermini L, Jonsson H, Alekseev AN, Crubezy E, Shapiro B, Orlando L: Shotgun microbial profiling of fossil remains. Mol Ecol 2014, 23(7):1780-1798)**.** Laboratory contamination is mostly excluded by our laboratory procedures and controls analyses (see Point 1).

We do not have access to the soil samples collected from exact archaeological site. Some of them were examined years ago. Moreover, soil metagenome might be affected by many factors such as season, weather, vegetation in the area which we are not able to control *post factum*.
However, to address the Reviewer suggestion to do additional controls (form soil/bone) to distinguish environmental contaminants we compared our sample microbial profiles with those previously published form soils (Fierer N, et al. (2012) Cross-biome metagenomic analyses of soil microbial communities and their functional attributes. Proc Natl Acad Sci U A 109(52):21390–21395.) and different human organs/fluids (The Human Microbiome Project Consortium (2012) Structure, function and diversity of the healthy human microbiome. Nature 486(7402):207–214.). PCoA results support the conclusion that the deposition soil is the main source of the microbial diversity present in the archeological samples (see Supplementary Figure 6).

Another technical issue is the removal of samples because they contain non-informative reads. I'd argue that all reads are informative, even if it's simply to tell you what the contaminant microbial species are within your samples.

For example, it remains unclear why the authors removed 8 samples with fewer than 1 million 'non-informative' reads. Several shotgun sequencing studies have shown that microbial profiles of samples can be obtained from even 100,000 sequences, so this filtering seems arbitrary. This type of filtering step is also done again on P5L14 and P5L57, and again the authors do not explain why these are non-informative or why entire samples are eliminated from the analysis? This especially makes no sense when the authors re-sample later to examine the validity of the data (P8L5).

In our study non-informative read is a read that did not map to the Metaphlan2 marker sequences. As shown in Supplementary Table 1, on average only 198 per 100,000 raw reads were informative in such sense (mapped to the markers). Such a low number of informative reads would bias the results as it would lead to the bacteria taxon identification based on a singular reads, and some taxa, present in a sample would be not identified at all. This phenomenon is clearly visible in our analysis of the sequencing depth influence on Metaphlan2 results (see Figure 6, Supplementary Figure 7 and Supplementary Table 3). Sample KO_30, with the second lowest number of raw reads, displays most significant differences in miocrobiome profile between shallow and deep datasets (correlation on a species level R=0.35). We clarified the selection criteria in the main text (page: 5, lines 120, 138; page 6 line 162).

2.) The high level of plant viruses is concerning, if the authors claim that they have retrieved an oral microbiome signal. Oral bacteria are often accompanied by a wide range of **oral bacteriophages.** This has been shown in a wide range modern studies (see Relman's group of publications on the oral virome, and large amounts on the gut virome) and ancient oral microbiota (see Warinner et al and Weyrich et al studies and reviews). If oral bacteriophages are absent, the odds of examining bacteria only from the oral microbiota seem low, and the odds of examining environmental microbiota with laboratory contamination are much greater.

Again, sorry for misleading statements (see the answer to Point 1). We do not claim that we analyzed oral microbiome, we analyzed microbiome of archaeological samples.
Inspired by the Reviewer, we examined phages that were identified within our study and indeed only very small fraction of this might represent ancient oral metagenome. We found: *Propionibacterium* - phage typical for oral bacteria. We added an appropriate comment in the main text (page 6 lines:154-155). We thank for this comment.

We also believe that the identification of oral bacteriophages in studies of modern oral microbiome are of great importance and authenticate the results. However, here we are limited by the depth of sequencing, dsDNA approach (viruses may by single stranded DNA or RNA) and the speed of viral DNA degradation (RNA viruses degrade faster, DNA viruses degradation may be different than bacterial or human). Thus, due to technical issues, we could not study viruses to such a degree as we studied bacteria. See also answer to the Reviewer 1, Point 3.

3.) Identifications to human-related microorganisms fall within groups of species that are also known to occur in the environment, or are so very closely related to environmental species that they cannot be easily identified (Metaphylan2 does a great job at picking out species specific markers, but these are not bullet proof, especially when only a handful of results arise from thousands of species present). For example, Bordtella pertussis was identified by the authors, yet this bacteria falls within a group of species that are so closely related that microbiologists have argued they should be classed as sub-species (B. bronchiseptica and B. pertussis). B. bronchiseptica can survive in the environment, and is present in a wide range of non-human animals, making the identification of B. parapertussis highly unlikely. This is a similar case for Clostridium tetani, as it is found in both the soil and as a human pathogen.

We are aware of the fact that every classification (MetaPhlaAn2, manual) will be a kind of approximation, possibly containing errors and simplifications. Yet, we do believe that selected groups represent fairly well environmental or human-associated species what is reflected in statistically significant differences in DNA damage observed in those two groups. Please see also answer to the next Point 4.

We are also aware that many human pathogens may be found in the environment. We thank the Reviewer for drawing our attention to this matter. We went throughout the manuscript and made sure we always use “potential human pathogens” expression. We also dedicated a separate paragraph in the Discussion to this issue (page 2 line: 47, page 7 lines: 187, 193; page 8 line: 209; page 10 line: 276; page 11 line: 293; page 13 line: 376).

4.) The manual curation of bacteria at the family level into groups of environmental or 'human-associated' simply is simply inappropriate (P6L44 onward). The authors would be very pressed to identify a bacterial family that contained ONLY human-associated bacteria, and not a wide range of environmental, human, and mammalian associated species. In addition, there are many genera of bacteria that are found in the mouth and gut (i.e. Clostridia), so a further breakdown of these categories into groups found in specific body sites is even more incomprehensible. The following downstream extrapolations from this break down (P7L10 onward) are meaningless.

We agree with the Reviewer that to a bacterial family may belong species typical for the environment as well as for human flora. However, our intention in this particular analysis was not the identification of certain species and their associations but to show a general sample characteristics. Furthermore, we also would like to explain that a family was classified as human-related only if all genera and all species identified in this family in particular samples were human-associated. We are sorry, that it was not clarified enough in the manuscript and introduced necessary comments in the main text.
Nonetheless, we agree with the Reviewer that the family level of classification might be too wide even for general microbiome characterization and re-analyzed samples on the genus level (slight differences were obtained, see Figure 4). We did not choose species level as not for all genera species were identified what might bias the results.
Lastly, we would like to kindly point out that only general habitat preferences (Figure 4) were inferred based on the family/genus level. All subsequent analyses (aDNA damage) were done on a species level – on a representative set of 77 genomes with undoubted association.

5.) It is unclear why the authors only analyze the levels of single stranded deamination, especially when mapDamage2.0 will analyze both simultaneously. The authors have referenced that they used a double stranded library preparation method, only creating sequence-able DNA fragments (i.e. those with Illumina adapters) from double stranded molecules. A specific, alternative library preparation method is needed to make libraries from single stranded molecules (Meyer, 2014; and from practice, this is not a method that would be used to create 161 ancient DNA libraries, as it is expensive and time consuming). Therefore, the DNA damage needs to be assessed on double stranded molecules. While I assume the trends would be relatively the same, the analysis that is completed by the authors technically makes no sense.

As the Reviewer suggested, we added analysis of deamination in dsDNA fragments (see Figure 7, Figure 8, Figure 9). As it is shown in Figures the dsDNA damage pattern well correspond to ssDNA damage, however due to much lesser frequency of dsDNA deamination the power and reliability of this analysis is lower. Additionally, in the Methods, we added information that standard double stranded library preparation protocol comprises single stranded overhangs reconstruction step, so analyses of deamination in ssDNA as well as in dsDNA are appropriate (page 5 line:116; page 14 lines: 403-405).

6.) This is a very interesting data set and resource, but without proper analytical approaches, the data as currently presented are meaningless. While several groups have successfully published microbial data from ancient teeth, this is done with painstaking numbers of controls and has been done on bacterial species known to cause blood born illnesses, i.e. would survive in the pulp of the teeth, such as the plague. There are undoubtedly some oral species that survive in the tooth environment, but the current lack of controls cannot confirm or deny that this is the case within this data set. In addition to the issues listed above, the analysis would be greatly improved if a clinical microbiome specialist could look over the data and aid in interpretations.

As previous studies of ancient bacteria were focused on the individual species or on a specific group of species (e.g. oral bacteria) our study is the first that describes microbiome of archaeological remains in a high number of samples of different age and type. We were not focused on human-related species, but on a global characterization of bacterial DNA present in ancient samples, no matter whether it is human or environmental or even form museum deposit environment.

Minor Points:

Another technical hurdle is the limitation of single end, 75 bp sequencing. This sequencing set up will limit in what is detected, i.e. it will preferentially examine more degraded DNA. While the goal of this study was to determine ancient microorganisms, more accurate identifications could have been made from larger endogenous fragments, i.e. 75 -150 bp fragments shown to exist in samples of this age. Accurate identification of species in metagenomes is heavily dependent upon length. While accurate identifications can be done from fragments this size, I wonder how much the species have been skewed due to the shorter sequencing lengths.

75 reads length was chosen for shallow DNA sequencing. Deep sequencing (50-150 million reads) of selected samples was performed on 100bp reads. The comparative analysis of obtained microbiomes from shallow and deep sequencing data showed very high correlation (see Figure 6, Supplementary Figure 7) what proved that reads of 75bp are sufficient for microbiome characteristics.

P2L20-28: The verb tense changes throughout this paragraph.

Corrected as suggested.

P4:26: Inclusion of minimally a carbon date from each location would strengthen the findings.

We added information on C14 dating in Supplementary Table 1.

P6L6: It should be Alpha.

Corrected.
