# Supplementary material for: Comprehensive analysis of microorganisms accompanying human archaeological remains
Source: Gigascience. 2017 Jun 13;6(7):1–13. doi: 10.1093/gigascience/gix044 (PMC5965364; doi:10.1093/gigascience/gix044)
Supplement: GIGA-D-17-00056_Revision_1.pdf [file gix044_giga-d-17-00056_revision_1.pdf]

GigaScience

Comprehensive analysis of microorganisms accompanying human archaeological remains

--Manuscript Draft--

**Manuscript Number:**	GIGA-D-17-00056R1
**Full Title:**	Comprehensive analysis of microorganisms accompanying human archaeological remains
**Article Type:**	Research
**Funding Information:**	Narodowe Centrum Nauki (2014/12/W/NZ2/00466) — Prof. Marek Figlerowicz
**Abstract:**	Background<br>Metagenome analyses has become a common source of information about microbial communities that occupy a wide range of niches, including archaeological specimens. It has been shown that vast majority of DNA extracted from ancient samples come from bacteria (presumably modern contaminants). However, characterization of microbial DNA accompanying human remains has never been done systematically for a wide range of different samples.<br>Findings<br>We used metagenomic approaches to perform comparative analyses of microorganism communities present in 161 archaeological human remains. DNA samples were isolated from the teeth of human skeletons dated from 100 AD to 1,200 AD. The skeletons were collected from seven archaeological sites in Central Europe and stored under different conditions. The majority of identified microbes were ubiquitous environmental bacteria that most likely contaminated the host remains not long ago. We observed that the composition of microbial communities was sample-specific and not correlated with its temporal or geographical origin. Additionally, traces of bacteria and archaea typical for human oral/gut flora as well as potential pathogens were identified in two-thirds of the samples. The genetic material of human-related species, in contrast to the environmental species that accounted for the majority of identified bacteria, displayed DNA damage patterns comparable with endogenous human aDNA, which suggested that these microbes might have accompanied the individual before death.<br>Conclusions<br>Our study showed that the microbiome observed in an individual sample is not reliant on the method or duration of sample storage. Moreover, shallow sequencing of DNA extracted from ancient specimens and subsequent bioinformatics analysis allowed both the identification of ancient microbial species, including potential pathogens, and their differentiation from contemporary species that colonized human remains more recently.
**Corresponding Author:**	Marek Figlerowicz<br>Institute of Bioorganic Chemistry PAS<br>Poznań, WLKP POLAND
**Corresponding Author Secondary Information:**	
**Corresponding Author's Institution:**	Institute of Bioorganic Chemistry PAS
**Corresponding Author's Secondary Institution:**	
**First Author:**	Anna Philips
**First Author Secondary Information:**	
**Order of Authors:**	Anna Philips
	Ireneusz Stolarek
	Bogna Kuczkowska
	Anna Juras

Powered by Editorial Manager® and ProduXion Manager® from Aries Systems Corporation

	Luiza Handschuh
	Janusz Piontek
	Piotr Kozłowski
	Marek Figlerowicz
**Order of Authors Secondary Information:**	
**Response to Reviewers:**	Dear Editor,<br>Once again, thank you for giving us the opportunity to revise our manuscript. We found all of your and the Reviewers' comments very useful in guiding us through the revision. Consequently, we considered all the points that were raised and modified our manuscript accordingly.<br><br>All introduced changes are marked in the revised manuscript in red. A detailed discussion of the Reviewers' comments is appended below in the point-by-point response. I hope the revised version of our manuscript will meet the high standards of the GigaScience journal.<br><br>Yours sincerely,<br>Marek Figlerowicz<br><br><br>Editor<br><br>Your manuscript "Comprehensive analysis of microorganisms accompanying human archaeological remains" (GIGA-D-17-00056) has been assessed by our reviewers. Based on these reports, and my own assessment as Editor, I am pleased to inform you that it is potentially acceptable for publication in GigaScience, once you have carried out some essential revisions suggested by our reviewers.<br>Their reports, together with any other comments, are below. Please also take a moment to check our website at http://giga.edmgr.com/ for any additional comments that were saved as attachments.<br>In addition to the reviewer's minor suggested changes below, please ensure you make the following edits to the paper:<br><br>1) Please add a Potential Implications section before "Methods" (see: https://academic.oup.com/gigascience//pages/research)<br><br>We added Potential Implications section, see page: 14, lines:397-405.<br><br>2) Under "Availability of Supporting data" please make the SRA URL into a Reference (#22) and add the URL to the References list.<br>3) Under "Availability of Supporting data" please also add the following sentence "Other data further supporting this work can be found in the GigaScience repository, GigaDB {Ref#23}."<br>Please add a place holder Ref #23 - to be updated with the GigaDB DOI (to be issued).<br>As requested, we added the two references, but at the end of the references list (INSTRUCTIONS TO AUTHORS - "All references, including URLs, must be numbered consecutively, in square brackets, in the order in which they are cited in the text").<br><br>Once you have made the necessary corrections, please submit a revised manuscript online at: http://giga.edmgr.com/<br><br>If you have forgotten your username or password please use the "Send Login Details" link to get your login information. For security reasons, your password will be reset.<br><br>Please include a point-by-point within the 'Response to Reviewers' box in the submission system. Please ensure you describe additional experiments that were carried out and include a detailed rebuttal of any criticisms or requested revisions that you disagreed with. Please also ensure that your revised manuscript conforms to the journal style, which can be found in the Instructions for Authors on the journal homepage.<br>The due date for submitting the revised version of your article is 10 Jul 2017.

Powered by Editorial Manager® and ProduXion Manager® from Aries Systems Corporation

Reviewer 1

Philips and colleagues satisfactorily addressed most of my comments, and I feel the paper has improved a lot.
But I am afraid I still have a few suggestions and one main concern:

1) Regarding my concern number 3 in the previous paper assessment (post-mortem damage in viruses). I understand the amount of virus markers covered by their sequencing data is low, but this should not be a limitation to know if virus DNA is ancient. Once they have identified the virus genera (and they claim to have done so), the authors can always download a bunch virus genomes of this genera, and remap all reads against them. If they manage to identify the correct species (or a closely-related one), the authors can perhaps retain an enough number of reads that is acceptable to assess post-mortem damage. They can just use mapDamage supplying the -no-stats flags, to obtain the raw plots without Bayesian parameter estimation. Simpler approaches can be also informative, like transitions/transversions ratios along the reads, or read length. I am VERY surprised by the large amount of virus DNA found (and also the amount of archea for some samples). My recollection is that we have never found something similar. In my opinion, further non-speculative understanding of this point would be very convenient.

As we have mentioned in our manuscript the most abundant viruses in the analyzed samples were RNA viruses of plants, e.g. Dasheen mosaic virus (58% of all identified viruses/viroids) and Vicia cryptic virus (26.7%). It would be extremely interesting to determine the pattern of their genomes damage, unfortunately at present it is rather impossible because their genomes are composed of RNA. All methods that are used for the damage analysis have been designed for organisms which genomes are composed of DNA. These methods do not take into account several important features that are characteristic for the replication of RNA virus genomes. RNA-based viruses encode their own RNA-dependent RNA polymerases that lack proofreading activity. As established in in vitro experiments, these enzyme exhibits an error rate that ranges from 10−4 to 10−6 per site. In addition to being error prone RNA replication is extremely efficient and in some cases it enables the production of 1012 virions per day in a single individual. As a result of highly effective but low-fidelity replication, the mutation rate of RNA virus genomes is several order of magnitude higher than the mutation rate of DNA genomes. Consequently, RNA virus does not form a homogenous population but circulates in a host organism as a pool of phylogenetically related distinct variants. To describe such a complex structure of virus population the concept of quasispecies is used.

Minor issues:
1) In pag 3 line 80, "rich" should be "reach".

Corrected.

2) I agree it is rare the case that microbial profiling is the main topic of an aDNA paper, but it does not mean ancient human remains have been "poorly" profiled. Perhaps rephrase to say that a comparative survey is lacking.

The Reviewer is right. We rephrased this sentence (see page: 4, line: 83).

3) although conclusions look sound, the statistics supporting their conclusions might not be the most appropriate. For example, no error bars in Fig.7.

As suggested, we added error bars in Figure 7.

What is correlation R? Do they mean Pearson correlation coefficient (r^2)? I cannot find it in methods. ANOVA assumes same variances for the groups being compared. I do not think it is the case in Fig. 1B and Fig 1C, for example. Actually, it would be interesting to know whether the older the site, the more variance in the endogenous content.

The Reviewer is right. R is Pearson correlation – an appropriate definition has been introduced (Methods, see page: 16, line: 455). We also replaced ANOVA test with non-

parametric Kruskal-Wallis (also called One-way ANOVA on ranks) test and accordingly, we modified the main text as the Reviewer suggested (see page: 5, lines:118-120; page 5, lines: 125-130).
We also added lacking methods in the Methods paragraph (see page:16, lines: 450-451; page: 16, lines: 453-454).

4) Metaphlan2 database could provide limited power to separate samples according to a categorical variable (age, conservation conditions, etc), because they are based on a limited number of markers from a limited number of taxa. I would strongly recommend trying to cluster samples based on k-mer distances between exogenous reads. This would provide an unbiased and complementary approach to corroborate absence of clustering, as the sample-specificity is one of their major claims. See for example https://bmcbioinformatics.biomedcentral.com/articles/10.1186/s12859-015-0875-7. Hope it helps!

We thank the Reviewer for this idea of samples clustering. As suggested, we run k-mer clustering, to check if approach in which we take into account all exogenous DNA reads will separate samples. Unfortunately we did not obtained clusters corresponding to the samples age, storage or archaeological site, what further supports our previews results. (see new Supplementary Figure 6, page: 9, lines: 237-239). Lastly, we would like to point out that suggested approach allows for analyses of not only microbial DNA reads, but also plants, animals, fungal reads. We suppose that this approach might introduce additional bias to the analyses and even might lead to the misleading results.

Reviewer 2

1) In their manuscript the authors describe a metagenomic assessment of archaeological remains with a focus on bacterial communities. The study targets multiple archaeological samples of varying age and storage conditions. The authors attempt to differentiate between bacterial species that might have been present before the individuals death and environmental contamination. The methods employed by the authors are suitable for metagenomic analyses and the applied statistics are appropriate. However, considering the fact that DNA of human endogenous pathogens is usually only found in very low amounts in archaeological samples, the concept of MetaPhlAn might lack sensitivity. Only reads mapping to predefined marker regions are used and therefore most of the data is discarded. This limits the possibilities for the authentication of species identifications.

We agree with the Reviewer that the identification of pathogens in shallow libraries (using MetaPhlAn) requires further confirmations (e.g. mapping to the whole microbial genome, experimental validation) and might be a good start for further study of certain pathogenic species, what was not the aim of this report see Discussion page: 14, lines: 393-394 and Potential Implications page: 14, lines: 402-404.
Our goal was to characterize the microbiome of archaeological samples and we do believe that identified microbes represent fairly well environmental or human-associated, pathogenic species. This fact is reflected in statistically significant differences in DNA damage observed in those groups (DNA damage analysis were done with reads mapping to the whole microbial genomes, not only marker sequences).

2) The authors discuss these limitations also in the context of the fact that many human related bacteria have close relatives in the soil. I recommend to discuss this a bit more specifically for individual taxa. Some suggestions are given below.

As suggested, we added adequate comments in the Discussion (see page 14, line:381; page: 14, lines:382-384). See also answer to the Point 4.

3) Overall, this study provides a useful comparative resource for the assessment of bacterial communities that accompany human remains in an archaeological context. In the abstract the authors claim that "comprehensive characterization of microbial DNA accompanying human remains has never been done." In fact many studies do comprehensively characterize microbial DNA in the context of human remains in addition to focusing on a specific pathogenic species. I assume what the authors mean by 'comprehensive' is that this has not been done systematically for a wide range of

Powered by Editorial Manager® and ProduXion Manager® from Aries Systems Corporation

different samples. The authors should make this clearer also in the introduction.

Corrected as the Reviewer suggested (see page: 2, lines:27-28).

4) The authors indicate that they found substantial amounts of Methanobrevibacter and describe it as "typically found in the human digestive system". It is very probable that the species M. oralis was detected here, which is commonly found in the oral cavity. Furthermore, the authors indicate that Bordetella was found as a taxon potentially representing a human pathogen. The authors mention in the discussion that B. bronchiseptica can also survive in the environment. In addition, it should be mentioned that this genus also contains species that are commonly found in the environment, such as B. petrii. The same is true for other taxa that the authors call 'potential pathogens' such as Stenotrophomonas and Clostridium. In particular for Clostridia it is rather unlikely that one of the individuals was infected with a bacterium of this genus. The reason that it is detected in the context of human remains is not surprising as it colonizes the human body after death. In general the authors only distinguish between human-related and environmental taxa. Bacteria involved in body decomposition overlap with both groups in some way. Therefore, I think that these bacteria should be mentioned as a third component in addition to human endogenous bacteria and environmental taxa.

We thank the Reviewer for this comment. We agree with the Reviewer that distinguishing between environmental and pathogenic species belonging to the same genus (like Bordetella) might be problematic, despites the usage of unique markers. This is why we believe that DNA damage patterns analysis might be helpful in preliminary study of ancient pathogens. See also answer to the Point 1.
We also realize that both environmental and human-associated species take part in body decomposition. However, despites a high number of analyzed samples deeper segregation would decrease statistical power of analyses and would made results non-conclusive. Similar situation was observed for division of human-related group into three subgroups (oral, pathogen, other) which caused the results to be of low statistical power and generally inconclusive.
According to the Reviewers suggestion we added appropriate comments in the Discussion (see page 12, line:319; page 14, line:381; page: 14, lines:382-384).

In the discussion the authors state "Our analyses revealed that some of them showed the DNA damage patterns closer to the environmental microbes than to the human associated ones." This statement does not make sense to me. They might be more similar to environmental taxa on the sequence level, but I do not see what that has to do with damage patterns.

We rephrased this sentence to be clearer (see page: 14, lines: 385-387). In this sentence we do not discuss the similarity between DNA sequences but similarity between DNA damage patterns. If we analyze human remains the DNA damage pattern observed for bacterial DNA is similar to the damage pattern observed for human aDNA one can assume that both are of similar age.

Furthermore, the authors state "This observation may indicate that DNA of some potential pathogens can be acquired from the environment after the organisms death." I do not understand the intention of this statement. Environmental contamination is obvious and one of the subjects of the study. Why should environmental taxa that are similar to human pathogens be excluded from this? I suggest to omit this statement.

We removed this sentence, as suggested.

Also in the discussion it is stated: "We showed that identification of bacteria/archaea species accompanying the organism before death is possible using standard aDNA extraction protocols and shallow shotgun sequencing." This is a very general statement. Other studies have shown that before to various extent. Maybe the authors can be more specific here. In addition, any bacterial taxon identified in this study should rather be seen as a candidate as the amount of data is not sufficient to authenticate the species identifications reliably.

The Reviewer is right. We changed this sentence according to the Reviewers comment

Powered by Editorial Manager® and ProduXion Manager® from Aries Systems Corporation

	(see page: 14, lines: 389).
**Additional Information:**	
**Question**	**Response**
Are you submitting this manuscript to a special series or article collection?	Yes
Please select an option from the menu: as follow-up to "Are you submitting this manuscript to a special series or article collection?"	Functional Metagenomics
**Experimental design and statistics**<br><br>Full details of the experimental design and statistical methods used should be given in the Methods section, as detailed in our Minimum Standards Reporting Checklist. Information essential to interpreting the data presented should be made available in the figure legends.<br><br>Have you included all the information requested in your manuscript?	Yes
**Resources**<br><br>A description of all resources used, including antibodies, cell lines, animals and software tools, with enough information to allow them to be uniquely identified, should be included in the Methods section. Authors are strongly encouraged to cite Research Resource Identifiers (RRIDs) for antibodies, model organisms and tools, where possible.<br><br>Have you included the information requested as detailed in our Minimum Standards Reporting Checklist?	Yes
**Availability of data and materials**<br><br>All datasets and code on which the conclusions of the paper rely must be either included in your submission or deposited in publicly available repositories (where available and ethically appropriate), referencing such data using a unique identifier in the references and in the "Availability of Data and Materials" section of your manuscript.<br><br>Have you have met the above requirement as detailed in our Minimum	Yes

Standards Reporting Checklist?	

Powered by Editorial Manager® and ProduXion Manager® from Aries Systems Corporation

Manuscript

Click here to download Manuscript aDNA_microorganisms_Figlerowicz_Revised_final-final.docx

Comprehensive analysis of microorganisms accompanying human archaeological remains

Anna Philips[1], Ireneusz Stolarek[1], Bogna Kuczkowska[1], Anna Juras[2], Luiza Handschuh[1,3,4], Janusz Piontek[2], Piotr Kozlowski[1,4,*], Marek Figlerowicz[1,5,*]

[1] European Center for Bioinformatics and Genomics, Institute of Bioorganic Chemistry, Polish Academy of Sciences, Poznan, 61-704, Poland

[2] Department of Human Evolutionary Biology, Institute of Anthropology, Faculty of Biology, Adam Mickiewicz University in Poznan, 61-614 Poznan, Poland

[3] Department of Hematology and Bone Marrow Transplantation, University of Medical Sciences, 60-569 Poznan, Poland

[4] Institute of Technology and Chemical Engineering, Poznan University of Technology, Poznan, 60-965, Poland

[5] Institute of Computing Science, Poznan University of Technology, Poznan, 60-965, Poland

* Correspondence: MF, tel. (+48) 61 852 8919, email marekf@ibch.poznan.pl; PK, tel. (+48) 61 852 8503, email kozlowp@ibch.poznan.pl.

ABSTRACT

Background

Metagenome analyses has become a common source of information about microbial communities that occupy a wide range of niches, including archaeological specimens. It has been shown that vast majority of DNA extracted from ancient samples come from bacteria (presumably modern contaminants). However, characterization of microbial DNA accompanying human remains has never been done systematically for a wide range of different samples.

Findings

We used metagenomic approaches to perform comparative analyses of microorganism communities present in 161 archaeological human remains. DNA samples were isolated from the teeth of human skeletons dated from 100 AD to 1,200 AD. The skeletons were collected from seven archaeological sites in Central Europe and stored under different conditions. The majority of identified microbes were ubiquitous environmental bacteria that most likely contaminated the host remains not long ago. We observed that the composition of microbial communities was sample-specific and not correlated with its temporal or geographical origin. Additionally, traces of bacteria and archaea typical for human oral/gut flora as well as potential pathogens were identified in two-thirds of the samples. The genetic material of human-related species, in contrast to the environmental species that accounted for the majority of identified bacteria, displayed DNA damage patterns comparable with endogenous human aDNA, which suggested that these microbes might have accompanied the individual before death.

Conclusions

Our study showed that the microbiome observed in an individual sample is not reliant on the method or duration of sample storage. Moreover, shallow sequencing of DNA extracted from ancient specimens and subsequent bioinformatics analysis allowed both the identification of ancient microbial species, including potential pathogens, and their differentiation from contemporary species that colonized human remains more recently.

Keywords

Microbiome, ancient DNA, NGS, metagenomics

BACKGROUND

During the last two decades, a number of methods that permit isolation and sequencing of ancient DNA (aDNA) extracted from archaeological specimens have been elaborated. As a result, several complete genome sequences of long-dead organisms have been determined [1-5]. Typically, aDNA is sampled from teeth or bones as these are the densest tissues in vertebrates, which supports the preservation of aDNA in crystal aggregates [6, 7]. Ancient remains are usually deposited in soils for decades, so DNA extracted is a mix of host DNA fragments and DNA form different organisms inhabiting the environment. To avoid the contamination that is usually present on bone/teeth surfaces (e.g., modern human, bacterial, fungal or plant DNA), aDNA is sampled from interior parts, where amount of aDNA is the highest. Despite applying rigorous DNA extraction protocols, the endogenous aDNA usually constitutes much less than 5% of the total extracted DNA, e.g., 1–5% for a Neanderthal [2] and 4% for a Mal'ta boy (24,000-year-old human) [8]. Of the remaining DNA, typically >>95% is DNA of different microorganisms that have colonized the remains and have been acquired from the environment. When younger remains are considered (100–200 years old), the amount of endogenous aDNA is not much higher [9]; however, it is possible to obtain a sample containing even up to 70% of endogenous aDNA [4, 10]. This is because the preservation of DNA depends on many environmental factors [11, 12]. For example, cold temperatures [13, 14], microclimate of caves where remains have been buried [11], or swampy sediments [12] are known to enhance DNA stability. Moreover, it has been shown that the vast majority of DNA isolated from archaeological human remains belongs to bacteria that have colonized the remains [15, 16]. Bacteria amplify the porosity of bone and teeth [17, 18], making it more accessible to water, which may lead to so-called endogenous aDNA leaching [19] and it replacing by exogenous DNA.

Some target enrichment procedures have been proposed to increase the amount of endogenous aDNA [20-24] and among them is the two-step digestion method [14, 25, 26]. Interestingly, Orlando and colleagues showed that two-step digestion does not influence the composition of bacterial communities (e.g., is the same in aDNA samples obtained after the first and second digestion runs) [9]. This observation suggests that niches exist deep within the bones and teeth. The environmental bacteria may reach these niches and preserve there.

Metagenome analysis has become a common source of information about microbial communities that occupy a wide range of niches. Until today, environmental components [27] as well as flora of

different human sites [28], e.g., oral [29, 30], skin [31] or intestinal [32-35], have been well characterized. In our study, we used this approach to analyze microorganisms that accompany archaeological human remains, which until now were not comparatively surveyed. Prior findings are limited to the rough identification of environmental bacteria [16] or concern a singular species, usually pathogenic. In the latter cases, the analyses were usually undertaken after the identification of visible symptoms of past disease [36-38]. Efforts have also been undertaken to characterize human mummy intestinal [39] and colon [40] microbes, as well as ancient oral microbiome ([41-44]). They showed that aDNA of species that colonized the organism before its death may be obtained. However, comprehensive characterization of microbial DNA accompanying human remains has never been done.

The current study was performed to characterize microorganisms associated with human archaeological remains. We used shotgun sequencing of DNA isolated from 161 human teeth collected from seven archaeological sites dated from 100 AD to 1,200 AD and stored under different conditions (e.g., museum or grave). For each individual sample its microbiome was determined using MetaPhlAn2 (Metagenomic Phylogenetic Analysis) based on multiple specific marker sequences derived from the genomes of microorganisms [45, 46]. Within this study, we focused on bacteria and archaea, which are known to constitute the majority of exogenous DNA in human archaeological remains [15, 16]. We checked whether microbial communities associated with specimens from different archaeological sites or of different ages were taxonomically and functionally distinct. We also attempted to identify microbes that may accompany the organism even before death and to distinguish bacteria/archaea that stem from *post-mortem* contamination from those of original flora by studying their DNA damage patterns.

DATA DESCRIPTION

We analyzed 161 human bone samples collected from seven archaeological sites in Central Europe (Figure 1 A). As shown in Table 1, the samples differed by age [Roman Age group (KO and MZ) or Medieval group (GO, SI, NA, ME, and LO)] and by storage conditions [specimens that were in museum deposits for at least 20 years (long deposit: KO, MZ, SI, NA, and GO), relatively freshly discovered specimens (stored in museum deposit <5 years, short deposit: LO) or samples taken directly from an archaeological site (Arch. site: ME)]. Carbon isotope dating of the selected samples

correlated well with dating based on archaeological analysis (see Supplementary Table 1). Ancient DNA was always extracted from the roots of teeth. We drilled those parts of the roots which include both dentine and cementum. In all cases enamel and cementum were preserved. Subsequently, all DNA samples were subjected for shallow NGS sequencing with the usage of an Illumina single-end standard protocol (including blunt-end DNA repair) and 75 bp sequencing run. Altogether, 846.5 million reads were obtained. On average, 98.6% of reads passed trimming and quality filtration. After filtration, for 161 samples, the average number of reads per sample was 5,143,975 (median: 4,730,243, range: 34,857 to 26,055,295). In further analysis, we removed eight samples that did not meet the arbitrary criterion of minimal raw reads number (<1 million). The average number of reads differ between archaeological sites (Kruskal-Wallis: p=0.01662), but not between samples type of storage (Wilcoxon: p=0.2685) or age (Wilcoxon: p=0.5607), Figure 1 B. Detailed information on each sample is summarized in Supplementary Table 1.

All reads were mapped to the reference human genome, and the percentage of human reads was determined for each sample. As shown in Figure 1 C, the fraction of human aDNA ranges from 0.01% to 91.9%; however, in most cases (100 samples), it was less than 5%. Nine samples had more than 50% of human aDNA content. Differences in the amount of human aDNA content were observed for different archeological sites (Kruskal-Wallis: p=6.124e-05), but not for freshly recovered and stored in museum samples (Wilcoxon: p=0.3160). The minor statistically significant difference was observed between older (KO, MZ) and younger (SI, NA, ME, GO, LO) samples (Wilcoxon: p=0.04669) with higher share of endogenous human DNA in older samples (average: 11.7% and 7.8%, median: 3.2% and 0.75%, for older and younger samples respectively).

ANALYSES

Microbiomes of human archaeological remains

To characterize the microbiomes of analyzed archaeological samples, we used MetaPhlAn2. The program identifies bacteria/archaea, viruses/viroids and unicellular eukaryotes using homology-based classification of NGS reads by alignment with predefined taxa-specific marker sequences [45]. The number of reads mapped to MetaPhlAn2 markers ranged from 708 (sample KO_014) to 95,950 (sample KO_006). Two samples with <1,000 reads mapped to the marker sequences were removed from further analyses as the marker coverage is crucial for proper microorganisms detection ([46]).

For the remaining 151 samples our analyses (Figure 2 A) showed that the majority of reads mapped to bacterial or archaeal markers (76.4%) and 23.4% to virus/viroid markers. The remaining 0.2% constituted eukaryotes (present in 13 samples; 0.6–8.2%), which were subsequently identified as fungi, protists or protozoa. The contribution of the particular types of microorganisms differed substantially between individual samples (in 12 samples, we found only bacteria; in sample KO_28, only viruses were identified; Figure 2 C). However, these differences did not correlate with archaeological site (MANOVA: p=0.0532, Figure 2 B), sample age (MANOVA: p=0.2054) or storage conditions (MANOVA: p=0.7672) .

The virus fraction varied from 0.1% to 99% between samples. Analysis of virus taxa showed that most of them were associated with plants; hence, we reasoned that they may have been acquired from the environment and were possibly indigenous flora. The most abundant viruses: *Dasheen mosaic virus* (58% of all identified viruses/viroids) and *Vicia cryptic virus* (26.7%) are both known to infect plants. Five viruses and one viroid constituted less than 2.5% each of all identified viruses/viroids, and also all were found to be associated with plant genera (*Ageratum, Sauropus, Cichorium* or *Malvastrum*). The remaining viruses were of low-abundance (<1%) and were usually present in no more than a single sample. It is also noteworthy, that we identified within our samples *Propionibacterium* phage - dsDNA virus that is associated with oral microbiome [47, 48]. Detailed information on the microorganism composition in individual samples is available in Supplementary Table 2.

Characterization of bacteria and archaea in human archaeological remains

In the next step, we focused on the prokaryotic component of the analyzed microbiomes. We decided to exclude from this analysis samples with a very high fraction of viruses/viroids. As a result, 11 samples with less than 1,000 reads mapping exclusively to bacterial/archaeal MetaPhlaAn2 marker sequences were removed as did not ensured a reliable microbiome profiling.

Altogether, 25 bacterial and 4 archaeal classes were identified in exogenous DNA of the analyzed samples, and among them, 6 bacterial classes accounted for >1% of identified bacteria/archaea. The most abundant classes were *Actinobacteria* (average 57%; range 0.18–98.9%), 3 classes of *Proteobacteria* [*Alphaproteobacteria* (6%; 0–65.5%), *Betaproteobacteria* (7%; 0–83.6%), *Gammaproteobacteria* (12%; 0–95.4%)], *Acidobacteria* (5%; 0–39.7%), and *Clostridia* (4%; 0–76.8%)

(Figure 3 A). Although most of the bacteria belonging to the first five classes are typically found in the environment (wide range of soils, waters) [27, 49], some of their taxa were human flora components. For example, *Corynebacterium matruchotii* (*Actinobacteria*) [50] and *Lautropia mirabilis* (*Betaproteobacteria*) [51, 52] represented more than 5% of the DNA in 4 samples: KO_046b, NA_121, NA_123, LO_166 and KO_005, KO_006, KO_046b, LO_166, respectively (Supplementary Table 2). *Clostridia* and *Bacteroidetes* are known to include many species inhabiting the human oral cavity or intestines [29]. Additionally, we found, in individual samples, markers characteristic for human pathogens, e.g., *Pseudoramibacter alactolyticus* (*Clostridia*) in sample MZ_88 [53] and *Bordetella parapertussis (Betaproteobacteria) [54]* in sample SI_084; *Clostridium sordellii* and *Clostridium tetani* [55, 56] (*Clostridia*) were found in 2 samples and 1 sample, respectively. Prokaryotic profiles differed substantially between individual samples (Figure 3 C) but did not differ between specific archaeological sites (MANOVA: p=0.3650, Figure 3 B), sample ages (MANOVA: p=0.3550) or storage conditions (MANOVA: p=0.4729). Similar high variation between individual samples and lack of specificity to archaeological sites were observed when prokaryotes were divided into groups based on gram +/- type (MANOVA: p=0.4364) or oxygen requirements (aerobic, facultative aerobic, anaerobic, facultative anaerobic, MANOVA: p=0.5726); see Supplementary Figure 1, Supplementary Figure 2.

The identification of singular prokaryotic taxa that are human- rather than environment-related motivated us to determine the fraction of microbes potentially associated with humans. All identified bacteria and archaea were divided on a genus level, into 2 groups: environmental and human-related. The latter was further divided into 3 subgroups: oral, potential pathogens and other (mostly gut). The genus characteristics were inferred based on the features of species identified by MetaPhlAn2. A genus was classified as human-related only if all species of this genus identified in our samples were human-related. The analysis showed that the majority (85.19%) of all bacteria/archaea were environmental (coming from soil and/or water); however, a substantial fraction of the investigated taxa (14.81%) were human-related, including 12.43% of microbes typical for human oral flora, 1.33% of potentially pathogenic bacteria and 1.05% of other (see Figure 4 A, B). As shown in Figure 4 C, the fraction of human-related genera varied significantly among samples, and some of these genera constituted most of the exogenous DNA. Although the fraction of human-related genera did not differ significantly between archaeological sites (one way ANOVA: p=0.7480), it was noteworthy that this fraction was highest in NA, the archaeological site dated to the Middle Ages, from which the samples

had been stored in a deposit for more than 20 years (see Figure 4 B). Interestingly, there was no relation between prevalence of human-related microbes and the levels of viruses/viroids accumulation or the level of endogenous human aDNA (see Supplementary Table 1). The identification of human-related species in ancient remains raised a question whether some of them accompanied the individual even before death.

Among all samples, the most frequent genera were the soil bacteria *Brevibacterium* (8.5% of all; present in 53 samples >1%; max. 71%) and *Kribbella* (8.4% of all; present in 60 samples >1%; max. 70%). The most abundant oral genera were *Bacteroidetes (*1.6% of all; present in 23 samples >1%; max. 28%), *Desulfobulbus (*1.4% of all; present in 25 samples >1%; max. 44%) and *Eubacterium* (1.4% of all; present in 20 samples >1%; max. 32%). *Methanobrevibacter* (0.8% of all; in 7 samples >1%; max. 34%), typically found in the human digestive system, was the most abundant taxon in other human-related group; *Bordetella* was the most abundant taxon classified as a potential human pathogen (*B. pertussis* is known to cause pertussis; 1.2% of all; in 16 samples >1%; max. 60%).

In general, 89% of the analyzed prokaryotes were aerobic or facultative aerobic (Supplementary Figure 1), and 63% were gram-positive (Supplementary Figure 2). However, in the human-related group only (Table 2), the percentage of aerobic or facultative aerobic taxa was smaller (24%, 49% and 54% for oral, pathogen, and other groups, respectively). Additionally, we found that the gram-negative prokaryotes dominated in the oral group (55%) and gram-positive prokaryotes in the other human-related group (70%). This slight dominance of gram-negative taxa in the oral group might be caused by lysozyme presence in an oral cavity that preferentially protects against gram-positive bacteria [57]. We additionally noticed that gram-negative species dominated (68%) in the potential pathogen group. These characteristics seem very useful for preliminary assessment of bacterial populations accompanying human remains.

To further investigate whether the prokaryotic profile permits classification of individual samples into specific groups (e.g., samples of similar age or storage conditions or samples from the same archaeological site), we performed Principal Coordinates Analysis (PCoA, Jaccard distance) on four taxonomic levels (class, family, genus, and species; Figure 5). Samples grouped into one big cluster in graphs created on all taxonomic levels. In the PCoA graphs generated on the family, genus and species levels, there was one more significantly smaller cluster visible. Importantly, none of these

clusters segregated samples according to the abovementioned features (age, storage, and site). Principal Component Analysis (PCA, see Supplementary Figure 3) and the Shannon diversity index (see Supplementary Table 1) again revealed high variation between individual samples at all analyzed taxonomic levels but did not show separation by sample source (species level, one way ANOVA: p=0.5660), sample age (species level, t-test: p=0.5535) or storage type (species level, t-test: p= 0.3516). We also tested a hypothesis that the occurrence of some human-related or environmental bacteria might be associated with archeological sites. We performed PCA analyses (Supplementary Figure 4) and hierarchical clustering (Supplementary Figure 5) on selected bacterial genera and found out that neither human-related nor environmental microbes segregated samples according to the archeological site, age or storage type. Finally, we clustered samples based on 10-mer distances between exogenous reads (see METHODS) and again observed no segregation according to the archeological site, age or storage type (Supplementary Figure 6).

In order to confirm that the major source of microbes observed in human archeological samples was the environment we compared their microbiomes with the microbiomes of human [58] and soils [27] by PCoA at the genus level (See Supplementary Figure 7).

Validation of data obtained using shallow sequencing

All results presented above were obtained with the use of datasets generated by relatively shallow sequencing (on average ~5 million reads per sample). To check the reliability of our results, we determined for the selected samples to what extent the composition of microbiomes (on the class level) is affected by the depth of sequencing. For this analysis, we used 11 representative samples differentiated in terms of: (1) filtered reads number obtained in the shallow sequencing experiment (~2-8 million), (2) number of reads mapped to the MetaPhlAn2 markers (~1–60 thousand), and (3) prokaryotic fraction (~10–90%). Eight samples were sequenced to the depth of ~50 million reads and 3 to the depth of ~100 million reads. Subsequently, we ran a MetaPhlAn2 profiling analysis on deep sequencing datasets. As expected, the total number of filtered reads as well as the number of reads mapping to the MetaPhlAn2 marker sequences increased significantly (about nine fold); however, the Shannon diversity indexes and microbial compositions remained intact (correlation R: 0.91-0.99, Figure 6 A, Supplementary Table 3). We obtained similar results when we analyzed three other taxonomic levels with somehow decreasing R with the depth of taxonomic level (average R: 0.96,

0.90, 0.88, 0.78 for class, family, genus and species levels, respectively Figure 6 B, Supplementary Figure 8). It is noteworthy that sample KO_030, second lower in the number of raw reads, displayed very low correlation R=0.35 on a species level when results obtained based on shallow sequencing (~2.6 million reads) and deep sequencing (47 million reads) were compared (Supplementary Figure 8, Supplementary Table 3). Overall, the correlation coefficient R and statistical significance ($p<0.0001$ in most cases, see Supplementary Figure 8) values were still very high and confirmed that the microbial profiles obtained based on the shallow sequencing datasets are reliable and do not change significantly when datasets generated in much deeper sequencing are used to establish them.

Analysis of age-related aDNA damage patterns

Finally, to verify whether identified human-related prokaryotes are ancient species that colonized the human body before death or are modern contaminants, we analyzed the signatures of age-related DNA damage. Age-related DNA damage was evaluated with the usage of mapDamage2.0 [59], which simulates the posterior distribution of (1) deamination in single strand DNA, ssDNA (δ_s), (2) deamination in double strand DNA, dsDNA (δ_d), and (3) the level of DNA fragmentation (λ, represented as: $1/\lambda$-1). [60, 61].

For this analysis, we used sequences of 77 complete genomes of the most representative prokaryotes of 313 identified in our samples (Supplementary Table 4). The 77 selected species consisted of 93% of all identified bacteria/archaea, and each of the selected species accounted for at least 10% in at least one sample (Supplementary Table 4A). The remaining species represented only 7% of the total microbial DNA, and they typically accounted for less than 1% of an individual sample. Subsequently, for each sample, we mapped all reads against (1) all 77 selected genomes; (2) a subset of 55 environmental bacteria genomes; (3) a subset of 14 oral bacteria genomes; (4) a subset of three gut bacteria and archaea genomes; (5) a subset of five potential pathogen genomes; and (6) a subset consisting of all human-related bacterial genomes (22 genomes; oral, gut and pathogens). Additionally, we mapped reads against a reference human genome to compare in each sample the level of DNA damage in human and microbial genomes. The comparison of DNA damage signatures in human and microbial DNA in individual samples are presented in Supplementary Figure 9 and Supplementary Figure 10.

As shown in Figure 7, the average DNA damage determined for all 77 microbial genomes

decreased with the increase in environmental bacteria fractions. Microbial DNA damage values differed significantly between samples with different fractions of environmental components (one way ANOVA: (δ_s) p=0.04130; (δ_d) p=0.00012; (1/λ-1) p<0.0001). The samples with the lowest (<25%) contribution of environmental bacteria displayed the highest level of microbial DNA damage (on average, δ_s=0.2643, δ_d=0.0067, 1/λ-1=2.7933), comparable with those observed for endogenous human aDNA (on average: δ_s=0.3571, δ_d=0.0279, 1/λ-1=1.6667). Noticeably, the damage of human aDNA did not depend on the amount of environmental bacteria in a sample (one way ANOVA: (δ_s) p=0.8630; (δ_d) p=0.3530; (1/λ-1) p=0.4770, Figure 7).

In the next step, for each sample, we calculated the DNA damage values separately for the following groups of bacterial species: (1) environmental; (2) all human-related; (3) oral; (4) gut and (5) potential pathogens. We compared these values with corresponding values determined for the endogenous human aDNA in the same sample. As is shown in Figure 8, the highest differences between the levels of human and microbial DNA damage were observed for environmental bacteria that showed very little DNA damage (average: $\Delta\delta_s$=0.1767; $\Delta\delta_d$=0.0264; Δ(1/λ-1)=1.3089). It is also shown in Figure 8 that the DNA damage of human-related species is similar to that observed for human aDNA (average: $\Delta\delta_s$=0.1224; $\Delta\delta_d$=0.0278; Δ(1/λ-1)=-0.8805). The variations in the obtained values may result from different rates of microbial DNA decay as well as from misclassification of some microbial species.

Actinobacteria, as well as all classes known to be non-spore forming, are more durable than other bacteria [62]. Thus, we used *Actinobacteria* (the most abundant class in our study) to analyze whether the differences between environmental and human-related species in DNA damage levels were influenced by different rates of damage in various microbe types. Within the human-related group (oral), we identified 3 species belonging to *Actinobacteria* present in 12 samples in >5%. Within the environmental group, we identified 12 species, present in 104 samples as >5%. The DNA damage patterns comparison again showed a higher damage rate in human-related rather than environmental *Actinobacteria* (t-test: (δ_s) p=0.0091; (δ_d) p=0.02987; (1/λ-1) p=0.0004, Figure 9). This finding confirmed that the larger accumulation of DNA damage observed for human-related species was not microbe type-specific. Therefore, different DNA damage levels in environmental and human-related bacteria did not result from differences in the stability of bacterial genomes but from their age.

DISCUSSION

This study represents one of the most comprehensive analyses of microbiomes that accompany ancient human skeletal remains. Accordingly, the analyzed DNA could come from (1) microorganisms that formed human microbiome and existed in human organism before death or (2) environmental species that contaminated human remains or participated in a body decomposition process.

In this study, we analyzed 161 datasets (total sequencing >63 Bbp) collected from seven different archaeological sites. We employed a novel approach based on a clade-specific genes analysis (MetaPhlAn2, [63]). This method relies on the database of marker sequences derived from whole genomes that unequivocally allows for the identification of microbial taxa down to the species level. Moreover, this method works not only for prokaryotes but also for all unicellular organisms and viruses. In contrast, a traditional approach based on the analysis of a singular 16S rRNA marker gene [64] is limited to the identification of bacteria/archaea at the genus level at most, thus being less accurate [65]. The applied methodology allowed us to determine the amount and type of viruses and fungi as well as bacteria and archaea in the analyzed samples. Notably, we showed that shallow sequencing (the average number of reads per analyzed sample was ~5 million) permitted retrieval of reliable microorganism profiles. The result validated using deeper sequencing (to 50–100 million reads) confirmed that our findings from shallow sequencing were trustworthy, although it has to be noted, that the accuracy slightly decreased with the taxonomic levels (Figure 6).

The thorough analysis of all microorganisms as well as only prokaryotes revealed that there are substantial differences between individual samples, but the differences were not characteristic for particular sample types. We showed that there was no correlation between the composition of microbial population and geographical place, sample age or storage history. This may suggest that pores in teeth constitute independent micro-environments, some easily accessible to an exogenous DNA, while others not (or temporarily not), which promotes the stochastic and unique microbial composition. Moreover, the comparison between museum specimens (more than 20 years from excavation) and relatively freshly sampled materials suggested that the treatment applied before storage (e.g., washing) and storage itself do not influence the microorganism composition in the teeth niches. Most likely, the migration of bacteria or a diffusion of microbial DNA and other microorganisms must be most intense when the remains are in direct contact with soil or water and negligible when placed in a relatively sterile environment, such as a museum deposit. These findings are of certain

importance, as they indicate that to study ancient microbiome museum specimens may be as good as freshly discovered specimens.

Overall, we identified 25 microbial classes, genetic material of 6 of them consisted more than 1% of all bacterial and archaeal DNA (Figure 3 A). Most of identified genera were ubiquitous bacteria belonging to the *Actinobacteria* class, such as *Brevibacterium, Kribbella, Actinoplanes* and *Streptosporangium,* which are typically found in a wide range of soils and waters (Figure 4). The obtained results are in line with previous findings ([15, 16, 49]) as well as with the common notion that DNA contamination of fossil remains comes from the soil and water. In addition, in some samples, we identified a substantial portion of microbes associated with the human body, mainly with the oral cavity, belonging predominantly to *Clostridia* (*Eubacterium*, *Pseudoramibacter*), *Actinobacteria* (*Propionibacterium, Corynebacterium, Actinomyces*) and *Bacteroidia* (*Tannerella*) classes. Moreover, we identified two bacterial one archaeal genera typical to the human digestive system, *Neisseria*, *Escherichia* (*Proteobacteria* class) and *Methanobrevibacter* (*Methanobacteria* class) as well as four potential human pathogens, *Bordetella*, *Stenotrophomonas*, *Bartonella* (*Proteobacteria* class) and *Clostridium* (*Clostridia* class).

DNA damage patterns analysis of the identified environmental and human-related microbes showed that the DNA of human-related species had significantly higher numbers of C→ T and G → A substitutions, which are typical for aDNA. Moreover, their damage levels were comparable with those observed for endogenous human aDNA in the corresponding samples (See Supplementary Figure 9, Supplementary Figure 10). According to the assumption that environmental microbes colonized archaeological bones relatively recently, DNA of environmental microbes displays a minimal amount of aDNA characteristic signatures. There is a possible bias caused by different dynamics of *post-mortem* DNA modifications in various bacteria types [66]. It has been shown that non-spore-forming *Actinobacteria* are more durable than endospore-formers such as *Bacillaceae* and *Clostridiaceae* [62]. The DNA damage analysis within the *Actinobacteria* class only revealed that human-related *Actinobacteria* species manifested aDNA damage patterns, and the environmental species showed the opposite pattern. This additional analysis supported our results and showed that the different levels of aDNA damage in environmental and human-related groups were not caused by the differences in bacterial genome stability. This also suggested that the identified human-related species may truly accompany the individual even before death. For environmental components, it seems that

their DNA is relatively young and must have had been acquired recently. One possible explanation is that some niches in the teeth are open and DNA exchange occurs continuously with the environment, whereas other niches are hardly accessible, so only endogenous species may reach and be preserved in these niches.

Many human pathogens belong to the same genera as environmental species [67]. For example, *Bordetella bronchiseptica* can survive in the environment, and is present in a wide range of animals [68, 69]. *Bordetella* genus also contains species that is commonly found in the environment, *Bordetella petrii*. *Clostridium tetani* is known to be the causative agent of tetanus, but is often found in soils and participates in body decomposition process. Hence, the identification of potential pathogen in body remains may not certainly mean that the individuals were infected with the bacterium before death. In fact, our analyses revealed that DNA of some of identified potential pathogens showed the DNA damage degree closer to the damage of environmental microbes than to the damage of human associated ones.

We showed that identification of candidate bacteria/archaea species accompanying the organism before death is possible using standard aDNA extraction protocols and shallow shotgun sequencing. The use of microbial markers derived from whole genomes is crucial, as aDNA typically lacks huge blocks of information, and using only the 16S rRNA gene as a marker may be not sufficient.

Our results indicated that not only fresh samples but also museum specimens seem to be good sources of ancient microbial DNA. Moreover, this methodology may be employed for screening remains without visible signs of disease, which provides the huge possibility of finding ancient pathogens for further analysis. In particular, this may provide additional knowledge to the fields of epidemiology and bacterial population genomics, allowing for the investigation of the rate of bacterial evolution, and may even bring forth some information on the ancient human diet.

POTENTIAL IMPLICATIONS

Here, we showed that the composition of microbiome of archeological remains does not depend on the way and time of storage. That opens a possibility to study on a wide range the microbiomes present in human and also non-human remains. We also demonstrated that it is possible to obtain reliable profiles of microbiomes form single-end shallow next-generation sequencing, what allow to cut time and costs of any microbiome study. Presented procedures might be used as a first step of ancient

pathogens identification, especially when a large set of samples with no apparent infection symptoms is considered. Finally, our studies revealed that by analyzing the DNA damage pattern one can identify the putative ancient microorganisms present in microbiome of archeological remains.

METHODS

Experimental procedures

DNA extraction from teeth was performed in the ancient DNA laboratory at the Faculty of Biology, Adam Mickiewicz University in Poznan. To avoid the contamination, that might be introduce through laboratory manipulations, all reagents used for DNA purification (buffers, water) and small plastic materials were UV irradiated (254 nm) per one hour. The surface of the teeth was cleaned with 0.5-5% NaOCl, rinsed with sterile and UV irradiated water, and then UV exposure (254 nm) for two hours per each site. Following UV irradiation, roots of teeth were drilled using Dremel® and bone powder was collected to sterile tubes (2 ml) and digested for 48 h in 56°C in a buffer containing EDTA, UREA and proteinase K as described in [70]. After digestion DNA was purified with MinElute kit (Qiagen) according to [71] and to [72]. Genomic libraries preparation was performed as described in [73]. The protocol comprises blunt-end repair step. A ssDNA overhanging 5'- and 3 '-ends are filled in or removed by T4 DNA polymerase. Typical T4 DNA polymerase removes 3'-overhangs and fills-in 5'-overhangs. Shallow sequencing was conducted following the Illumina single-end standard protocol on GAIIx using a 75 bp sequencing run. Deep Sequencing was conducted following the Illumina pair-end standard protocol on GAIIx using a 100 bp sequencing run.

Contamination control

DNA contamination from the laboratory environment and reagents was controlled through setting up negative controls during DNA extraction, genomic libraries preparation and amplification in parallel with the samples at all experimental steps. DNA concentrations in negative controls were undetectable with Qubit dsDNA HS Assay (Thermo Fisher Scientific) and Bioanalyzer 2100 HS DNA Assay (Agilent), implying concentrations below 0.01 ng/uL. Concentrations of the libraries built from ancient human teeth were between 1.1 and 125.5 ng/uL (on average 18.76 ng/uL). The amount of DNA in negative controls was at least 100 fold lower than for ancient samples and was not subjected to the sequencing.

Bioinformatics procedures

All reads were trimmed and adapters were removed using the AdapterRemoval tool [74]. The minimal length of reads was set to 25 and the minimal base quality was set to 30.

To investigate the composition of microbial communities in each sample, we used the MetaPhlAn2 program with default settings [46]. To avoid bias in the assessment of microorganism abundance, we mapped (using Bowtie2 [75] and the recommended sensitive global alignment strategy) all reads against the MetaPhlAn2 markers database and removed PCR duplicates with Picard MarkDuplicates tool 1.82 (https://broadinstitute.github.io/picard/). Next, we ran MetaPhlAn2 with the option "-a" to determine all taxonomic levels.

To assess the amount of endogenous DNA, reads were mapped (Bowtie2) against human nuclear (hg19, [76]) and complete mitochondrial genomes (GenBank Accession no. NC 012920.1, [77]).

To investigate aDNA damage patterns, we employed mapDamage2.0 (with the default settings) [59]. All plots were generated using R 3.3.2 (ggplot2 package).

Statistical analysis

Shannon diversity, principal component PCA and principal coordinate PCoA analysis on 4 taxonomic levels (class, genus, family, species) were run in R (functions: diversity(), prcomp() and pcoa(), respectively) for all identified microorganisms and for bacteria/archaea only. PCoA was run on the Jaccard and Bray-Curtis distance tables calculated from the taxon abundance. To determine whether low abundance taxa (<1%) may have influenced the analysis, we also ran PCoA without them (data not shown). To determine if *k*-mers of exogenous reads might segregate samples according to their age, storage or archeological site we followed approach described in [78].

To test if certain groups displayed statistically significant differences, we applied: a one-way ANOVA, followed by a Tukey HSD and a t-test [R functions: aov(), TukeyHSD(), t.test()] as well as non-parametric tests: Kruskal-Wallis and Wilcoxon [R functions: kruskal.test(), wilcox.test()] .

Correlation R was calculated as Pearson correlation coefficient.

AVAILABILITY OF SUPPORTING DATA AND MATERIALS

Other data further supporting this work can be found in the GigaScience repository, GigaDB [79]. The

datasets supporting the conclusions of this article are available in the NCBI Sequence Read Archive (SRA) repository, SRP093814 [80].

DECLARATIONS

List of abbreviations

aDNA – ancient DNA; dsDNA – double stranded DNA; NCBI – National Center for Biotechnology Information; NGS – next generation sequencing; ssDNA – single stranded DNA; SRA – Sequence Read Archive.

Ethics approval and consent to participate

Not applicable.

Consent for publication

Not applicable.

Competing interests

The authors declare that they have no competing interests.

Acknowledgment

We thank Wioletta Nowaczewska for providing samples from Masłomęcz.

Funding

This work was supported by polish National Science Center [2014/12/W/NZ2/00466]

Authors' contributions

AP conceived the study, participated in the study design, analyzed the data, discussed the results, and wrote the manuscript; IS participated in the statistical analysis, figures preparation and submitted the datasets to SRA; BK ran preliminary Metaphlan2 analysis; AJ extracted DNA and participated in NGS library preparation; LH prepared NGS libraries and run NGS; JP: participated in results discussion; PK participated in the study design, analyzed and discussed the data and participated in

drafting the manuscript; MF conceived the overall idea of the study, participated in the study design, analyzed and discussed the data, coordinated studies, was responsible for the final version of the manuscript; All authors read and approved the final manuscript.

REFERENCES

1. Fu Q, Posth C, Hajdinjak M, Petr M, Mallick S, Fernandes D, Furtwangler A, Haak W, Meyer M, Mittnik A et al: The genetic history of Ice Age Europe. Nature 2016.

2. Green RE, Krause J, Briggs AW, Maricic T, Stenzel U, Kircher M, Patterson N, Li H, Zhai W, Fritz MH et al: A draft sequence of the Neandertal genome. Science 2010, 328(5979):710-722.

3. Rasmussen M, Li Y, Lindgreen S, Pedersen JS, Albrechtsen A, Moltke I, Metspalu M, Metspalu E, Kivisild T, Gupta R et al: Ancient human genome sequence of an extinct Palaeo-Eskimo. Nature 2010, 463(7282):757-762.

4. Meyer M, Kircher M, Gansauge MT, Li H, Racimo F, Mallick S, Schraiber JG, Jay F, Prufer K, de Filippo C et al: A high-coverage genome sequence from an archaic Denisovan individual. Science 2012, 338(6104):222-226.

5. Librado P, Fages A, Gaunitz C, Leonardi M, Wagner S, Khan N, Hanghoj K, Alquraishi SA, Alfarhan AH, Al-Rasheid KA et al: The Evolutionary Origin and Genetic Makeup of Domestic Horses. Genetics 2016, 204(2):423-434.

6. Malmstrom H, Stora J, Dalen L, Holmlund G, Gotherstrom A: Extensive human DNA contamination in extracts from ancient dog bones and teeth. Mol Biol Evol 2005, 22(10):2040-2047.

7. Salamon M, Tuross N, Arensburg B, Weiner S: Relatively well preserved DNA is present in the crystal aggregates of fossil bones. Proc Natl Acad Sci U S A 2005, 102(39):13783-13788.

8. Raghavan M, Skoglund P, Graf KE, Metspalu M, Albrechtsen A, Moltke I, Rasmussen S, Stafford TW, Jr., Orlando L, Metspalu E et al: Upper Palaeolithic Siberian genome reveals dual ancestry of Native Americans. Nature 2014, 505(7481):87-91.

9. Der Sarkissian C, Ermini L, Jonsson H, Alekseev AN, Crubezy E, Shapiro B, Orlando L: Shotgun microbial profiling of fossil remains. Mol Ecol 2014, 23(7):1780-1798.

10. Reich D, Green RE, Kircher M, Krause J, Patterson N, Durand EY, Viola B, Briggs AW, Stenzel U, Johnson PL et al: Genetic history of an archaic hominin group from Denisova Cave in Siberia. Nature 2010, 468(7327):1053-1060.

11. Ovchinnikov IV, Gotherstrom A, Romanova GP, Kharitonov VM, Liden K, Goodwin W: Molecular analysis of Neanderthal DNA from the northern Caucasus. Nature 2000, 404(6777):490-493.

12. Lawlor DA, Dickel CD, Hauswirth WW, Parham P: Ancient HLA genes from 7,500-year-old archaeological remains. Nature 1991, 349(6312):785-788.

13. Smith CI, Chamberlain AT, Riley MS, Cooper A, Stringer CB, Collins MJ: Neanderthal DNA. Not just old but old and cold? Nature 2001, 410(6830):771-772.

14. Schwarz C, Debruyne R, Kuch M, McNally E, Schwarcz H, Aubrey AD, Bada J, Poinar H: New

insights from old bones: DNA preservation and degradation in permafrost preserved mammoth remains. Nucleic Acids Res 2009, 37(10):3215-3229.

15. Poinar HN, Schwarz C, Qi J, Shapiro B, Macphee RD, Buigues B, Tikhonov A, Huson DH, Tomsho LP, Auch A et al: Metagenomics to paleogenomics: large-scale sequencing of mammoth DNA. Science 2006, 311(5759):392-394.

16. Noonan JP, Hofreiter M, Smith D, Priest JR, Rohland N, Rabeder G, Krause J, Detter JC, Paabo S, Rubin EM: Genomic sequencing of Pleistocene cave bears. Science 2005, 309(5734):597-599.

17. Sampietro ML, Gilbert MT, Lao O, Caramelli D, Lari M, Bertranpetit J, Lalueza-Fox C: Tracking down human contamination in ancient human teeth. Mol Biol Evol 2006, 23(9):1801-1807.

18. Jans MME, Nielsen-Marsh CM, Smith CI, Collins MJ, Kars H: Characterisation of microbial attack on archaeological bone. J Archaeol Sci 2004, 31(1):87-95.

19. Haile J, Holdaway R, Oliver K, Bunce M, Gilbert MT, Nielsen R, Munch K, Ho SY, Shapiro B, Willerslev E: Ancient DNA chronology within sediment deposits: are paleobiological reconstructions possible and is DNA leaching a factor? Mol Biol Evol 2007, 24(4):982-989.

20. Carpenter ML, Buenrostro JD, Valdiosera C, Schroeder H, Allentoft ME, Sikora M, Rasmussen M, Gravel S, Guillen S, Nekhrizov G et al: Pulling out the 1%: whole-genome capture for the targeted enrichment of ancient DNA sequencing libraries. Am J Hum Genet 2013, 93(5):852-864.

21. Schuenemann VJ, Singh P, Mendum TA, Krause-Kyora B, Jager G, Bos KI, Herbig A, Economou C, Benjak A, Busso P et al: Genome-wide comparison of medieval and modern Mycobacterium leprae. Science 2013, 341(6142):179-183.

22. Gansauge MT, Meyer M: Selective enrichment of damaged DNA molecules for ancient genome sequencing. Genome Res 2014, 24(9):1543-1549.

23. Avila-Arcos MC, Cappellini E, Romero-Navarro JA, Wales N, Moreno-Mayar JV, Rasmussen M, Fordyce SL, Montiel R, Vielle-Calzada JP, Willerslev E et al: Application and comparison of large-scale solution-based DNA capture-enrichment methods on ancient DNA. Sci Rep 2011, 1:74.

24. Cruz-Davalos DI, Llamas B, Gaunitz C, Fages A, Gamba C, Soubrier J, Librado P, Seguin-Orlando A, Pruvost M, Alfarhan AH et al: Experimental conditions improving in-solution target enrichment for ancient DNA. Molecular ecology resources 2016.

25. Orlando L, Ginolhac A, Raghavan M, Vilstrup J, Rasmussen M, Magnussen K, Steinmann KE, Kapranov P, Thompson JF, Zazula G et al: True single-molecule DNA sequencing of a pleistocene horse bone. Genome Res 2011, 21(10):1705-1719.

26. Ginolhac A, Vilstrup J, Stenderup J, Rasmussen M, Stiller M, Shapiro B, Zazula G, Froese D, Steinmann KE, Thompson JF et al: Improving the performance of true single molecule sequencing for ancient DNA. BMC Genomics 2012, 13:177.

27. Fierer N, Leff JW, Adams BJ, Nielsen UN, Bates ST, Lauber CL, Owens S, Gilbert JA, Wall DH, Caporaso JG: Cross-biome metagenomic analyses of soil microbial communities and their functional attributes. Proc Natl Acad Sci U S A 2012, 109(52):21390-21395.

28. Ding T, Schloss PD: Dynamics and associations of microbial community types across the human body. Nature 2014, 509(7500):357-360.

29. Wade WG: The oral microbiome in health and disease. Pharmacol Res 2013, 69(1):137-143.

30. Xu X, He J, Xue J, Wang Y, Li K, Zhang K, Guo Q, Liu X, Zhou Y, Cheng L et al: Oral cavity contains distinct niches with dynamic microbial communities. Environ Microbiol 2015, 17(3):699-710.

31. Ferretti P, Farina S, Cristofolini M, Girolomoni G, Tett A, Segata N: Experimental metagenomics and ribosomal profiling of the human skin microbiome. Experimental dermatology 2016.

32. O'Toole PW, Jeffery IB: Gut microbiota and aging. Science 2015, 350(6265):1214-1215.

33. Zhernakova A, Kurilshikov A, Bonder MJ, Tigchelaar EF, Schirmer M, Vatanen T, Mujagic Z, Vila AV, Falony G, Vieira-Silva S et al: Population-based metagenomics analysis reveals markers for gut microbiome composition and diversity. Science 2016, 352(6285):565-569.

34. Falony G, Joossens M, Vieira-Silva S, Wang J, Darzi Y, Faust K, Kurilshikov A, Bonder MJ, Valles-Colomer M, Vandeputte D et al: Population-level analysis of gut microbiome variation. Science 2016, 352(6285):560-564.

35. Donaldson GP, Lee SM, Mazmanian SK: Gut biogeography of the bacterial microbiota. Nat Rev Microbiol 2016, 14(1):20-32.

36. Rasmussen S, Allentoft ME, Nielsen K, Orlando L, Sikora M, Sjogren KG, Pedersen AG, Schubert M, Van Dam A, Kapel CM et al: Early divergent strains of Yersinia pestis in Eurasia 5,000 years ago. Cell 2015, 163(3):571-582.

37. Maixner F, Krause-Kyora B, Turaev D, Herbig A, Hoopmann MR, Hallows JL, Kusebauch U, Vigl EE, Malfertheiner P, Megraud F et al: The 5300-year-old Helicobacter pylori genome of the Iceman. Science 2016, 351(6269):162-165.

38. Seifert L, Wiechmann I, Harbeck M, Thomas A, Grupe G, Projahn M, Scholz HC, Riehm JM: Genotyping Yersinia pestis in Historical Plague: Evidence for Long-Term Persistence of Y. pestis in Europe from the 14th to the 17th Century. PLoS One 2016, 11(1):e0145194.

39. Rollo F, Ermini L, Luciani S, Marota I, Olivieri C: Studies on the preservation of the intestinal microbiota's DNA in human mummies from cold environments. Med Secoli 2006, 18(3):725-740.

40. Ubaldi M, Luciani S, Marota I, Fornaciari G, Cano RJ, Rollo F: Sequence analysis of bacterial DNA in the colon of an Andean mummy. Am J Phys Anthropol 1998, 107(3):285-295.

41. Weyrich LS, Dobney K, Cooper A: Ancient DNA analysis of dental calculus. J Hum Evol 2015, 79:119-124.

42. Warinner C, Speller C, Collins MJ, Lewis CM, Jr.: Ancient human microbiomes. J Hum Evol 2015, 79:125-136.

43. Warinner C, Rodrigues JF, Vyas R, Trachsel C, Shved N, Grossmann J, Radini A, Hancock Y, Tito RY, Fiddyment S et al: Pathogens and host immunity in the ancient human oral cavity. Nat Genet 2014, 46(4):336-344.

44. Adler CJ, Dobney K, Weyrich LS, Kaidonis J, Walker AW, Haak W, Bradshaw CJ, Townsend G, Soltysiak A, Alt KW et al: Sequencing ancient calcified dental plaque shows changes in oral microbiota with dietary shifts of the Neolithic and Industrial revolutions. Nat Genet 2013, 45(4):450-455, 455e451.

45. Segata N, Izard J, Waldron L, Gevers D, Miropolsky L, Garrett WS, Huttenhower C: Metagenomic

biomarker discovery and explanation. Genome Biol 2011, 12(6):R60.

46. Truong DT, Franzosa EA, Tickle TL, Scholz M, Weingart G, Pasolli E, Tett A, Huttenhower C, Segata N: MetaPhlAn2 for enhanced metagenomic taxonomic profiling. Nat Methods 2015, 12(10):902-903.

47. Pride DT, Salzman J, Haynes M, Rohwer F, Davis-Long C, White RA, 3rd, Loomer P, Armitage GC, Relman DA: Evidence of a robust resident bacteriophage population revealed through analysis of the human salivary virome. The ISME journal 2012, 6(5):915-926.

48. Willner D, Furlan M, Schmieder R, Grasis JA, Pride DT, Relman DA, Angly FE, McDole T, Mariella RP, Jr., Rohwer F et al: Metagenomic detection of phage-encoded platelet-binding factors in the human oral cavity. Proc Natl Acad Sci U S A 2011, 108 Suppl 1:4547-4553.

49. Metcalf JL, Xu ZZ, Weiss S, Lax S, Van Treuren W, Hyde ER, Song SJ, Amir A, Larsen P, Sangwan N et al: Microbial community assembly and metabolic function during mammalian corpse decomposition. Science 2016, 351(6269):158-162.

50. Tsuzukibashi O, Uchibori S, Shinozaki-Kuwahara N, Kobayashi T, Takada K, Hirasawa M: A selective medium for the isolation of Corynebacterium species in oral cavities. J Microbiol Methods 2014, 104:67-71.

51. Colombo AP, Boches SK, Cotton SL, Goodson JM, Kent R, Haffajee AD, Socransky SS, Hasturk H, Van Dyke TE, Dewhirst F et al: Comparisons of subgingival microbial profiles of refractory periodontitis, severe periodontitis, and periodontal health using the human oral microbe identification microarray. J Periodontol 2009, 80(9):1421-1432.

52. Shaddox LM, Huang H, Lin T, Hou W, Harrison PL, Aukhil I, Walker CB, Klepac-Ceraj V, Paster BJ: Microbiological characterization in children with aggressive periodontitis. J Dent Res 2012, 91(10):927-933.

53. Antunes HS, Rocas IN, Alves FR, Siqueira JF, Jr.: Total and Specific Bacterial Levels in the Apical Root Canal System of Teeth with Post-treatment Apical Periodontitis. J Endod 2015, 41(7):1037-1042.

54. Javed S, Said F, Eqani SA, Bokhari H: Bordetella parapertussis outbreak in Bisham, Pakistan in 2009-2010: fallout of the 9/11 syndrome. Epidemiol Infect 2015, 143(12):2619-2623.

55. Vidor C, Awad M, Lyras D: Antibiotic resistance, virulence factors and genetics of Clostridium sordellii. Res Microbiol 2015, 166(4):368-374.

56. Hanif H, Anjum A, Ali N, Jamal A, Imran M, Ahmad B, Ali MI: Isolation and Antibiogram of Clostridium tetani from Clinically Diagnosed Tetanus Patients. Am J Trop Med Hyg 2015, 93(4):752-756.

57. Endersen L, Coffey A, Ross RP, McAuliffe O, Hill C, O'Mahony J: Characterisation of the antibacterial properties of a bacterial derived peptidoglycan hydrolase (LysCs4), active against C. sakazakii and other Gram-negative food-related pathogens. International journal of food microbiology 2015, 215:79-85.

58. Human Microbiome Project C: Structure, function and diversity of the healthy human microbiome. Nature 2012, 486(7402):207-214.

59. Jonsson H, Ginolhac A, Schubert M, Johnson PL, Orlando L: mapDamage2.0: fast approximate

Bayesian estimates of ancient DNA damage parameters. Bioinformatics 2013, 29(13):1682-1684.

60. Briggs AW, Stenzel U, Johnson PL, Green RE, Kelso J, Prufer K, Meyer M, Krause J, Ronan MT, Lachmann M et al: Patterns of damage in genomic DNA sequences from a Neandertal. Proc Natl Acad Sci U S A 2007, 104(37):14616-14621.

61. Sawyer S, Krause J, Guschanski K, Savolainen V, Paabo S: Temporal patterns of nucleotide misincorporations and DNA fragmentation in ancient DNA. PLoS One 2012, 7(3):e34131.

62. Willerslev E, Hansen AJ, Ronn R, Brand TB, Barnes I, Wiuf C, Gilichinsky D, Mitchell D, Cooper A: Long-term persistence of bacterial DNA. Curr Biol 2004, 14(1):R9-10.

63. Schubert M, Ermini L, Der Sarkissian C, Jonsson H, Ginolhac A, Schaefer R, Martin MD, Fernandez R, Kircher M, McCue M et al: Characterization of ancient and modern genomes by SNP detection and phylogenomic and metagenomic analysis using PALEOMIX. Nat Protoc 2014, 9(5):1056-1082.

64. DeSantis TZ, Hugenholtz P, Larsen N, Rojas M, Brodie EL, Keller K, Huber T, Dalevi D, Hu P, Andersen GL: Greengenes, a chimera-checked 16S rRNA gene database and workbench compatible with ARB. Appl Environ Microbiol 2006, 72(7):5069-5072.

65. Wang Q, Garrity GM, Tiedje JM, Cole JR: Naive Bayesian classifier for rapid assignment of rRNA sequences into the new bacterial taxonomy. Appl Environ Microbiol 2007, 73(16):5261-5267.

66. Setlow P: Mechanisms for the prevention of damage to DNA in spores of Bacillus species. Annu Rev Microbiol 1995, 49:29-54.

67. Bouwman AS, Kennedy SL, Muller R, Stephens RH, Holst M, Caffell AC, Roberts CA, Brown TA: Genotype of a historic strain of Mycobacterium tuberculosis. Proc Natl Acad Sci U S A 2012, 109(45):18511-18516.

68. Weyrich LS, Rolin OY, Muse SJ, Park J, Spidale N, Kennett MJ, Hester SE, Chen C, Dudley EG, Harvill ET: A Type VI secretion system encoding locus is required for Bordetella bronchiseptica immunomodulation and persistence in vivo. PLoS One 2012, 7(10):e45892.

69. Bendor L, Weyrich LS, Linz B, Rolin OY, Taylor DL, Goodfield LL, Smallridge WE, Kennett MJ, Harvill ET: Type Six Secretion System of Bordetella bronchiseptica and Adaptive Immune Components Limit Intracellular Survival During Infection. PLoS One 2015, 10(10):e0140743.

70. Juras A, Chylenski M, Krenz-Niedbala M, Malmstrom H, Ehler E, Pospieszny L, Lukasik S, Bednarczyk J, Piontek J, Jakobsson M et al: Investigating kinship of Neolithic post-LBK human remains from Krusza Zamkowa, Poland using ancient DNA. Forensic science international Genetics 2017, 26:30-39.

71. Yang DY, Eng B, Waye JS, Dudar JC, Saunders SR: Technical note: improved DNA extraction from ancient bones using silica-based spin columns. Am J Phys Anthropol 1998, 105(4):539-543.

72. Malmstrom H, Svensson EM, Gilbert MT, Willerslev E, Gotherstrom A, Holmlund G: More on contamination: the use of asymmetric molecular behavior to identify authentic ancient human DNA. Mol Biol Evol 2007, 24(4):998-1004.

73. Meyer M, Kircher M: Illumina sequencing library preparation for highly multiplexed target capture and sequencing. Cold Spring Harbor protocols 2010, 2010(6):pdb prot5448.

74. Schubert M, Lindgreen S, Orlando L: AdapterRemoval v2: rapid adapter trimming, identification, and read merging. BMC Res Notes 2016, 9:88.

75. Langmead B, Salzberg SL: Fast gapped-read alignment with Bowtie 2. Nat Methods 2012, 9(4):357-359.

76. Meyer LR, Zweig AS, Hinrichs AS, Karolchik D, Kuhn RM, Wong M, Sloan CA, Rosenbloom KR, Roe G, Rhead B et al: The UCSC Genome Browser database: extensions and updates 2013. Nucleic Acids Res 2013, 41(Database issue):D64-69.

77. Andrews RM, Kubacka I, Chinnery PF, Lightowlers RN, Turnbull DM, Howell N: Reanalysis and revision of the Cambridge reference sequence for human mitochondrial DNA. Nat Genet 1999, 23(2):147.

78. Dubinkina VB, Ischenko DS, Ulyantsev VI, Tyakht AV, Alexeev DG: Assessment of k-mer spectrum applicability for metagenomic dissimilarity analysis. BMC bioinformatics 2016, 17:38.

79. Philips A, Stolarek I, Kuczkowska B, Juras A, Handschuh L, Piontek J, Kozlowski P, Figlerowicz M: Supporting data for "Comprehensive analysis of microorganisms accompanying human archaeological remains" GigaScience Database. 2017 (GigaDB DOI to be issued).

80. Sequence Read Archive. https://www.ncbi.nlm.nih.gov/sra/?term=SRP093814. Accessed 24 Nov. 2016.

TABLE AND FIGURE LEGENDS

Table 1. Characteristics of the samples extracted from ancient human remains.

Archaeological site	ID	Sample no.	Sample no. that passed selection	Dating	Date of excavation	Storage conditions	Sample type
**Roman Age Group**							
Kowalewko	KO	58	48	100AD-300AD	1990s	Long deposit	Tooth
Masłomęcz	MZ	27	24	200AD-400AD	1970-1990	Long deposit	Tooth
**Medieval Group**							
Sowinki	SI	21	19	1000AD-1100AD	1980s	Long deposit	Tooth
Niemcza	NA	36	31	900AD-1000AD	1960s	Long deposit	Tooth
Markowice	ME	8	8	1000AD-1200AD	2014	Arch. site	Tooth
Gniezno	GO	2	2	1000AD-1200AD		Long deposit	Tooth
Łęgowo	LO	9	8	1000AD-1200AD	2013-2015	Short deposit	Tooth

Table 2. The percentage of bacteria/archaea of a given respiratory type [(facultative) aerobic/anaerobic] and gram stain type [positive/negative] within environmental and human-related groups [oral, pathogenic, or other].

Group	(Facultative) anaerobic	(Facultative) aerobic	Gram-positive	Gram-negative
Environmental	4%	96%	66%	34%
Oral	76%	24%	45%	55%
Pathogenic	51%	49%	32%	68%
Other human-related	46%	54%	70%	30%

Figure 1. A) The geographical positions of archaeological sites. KO and MZ are from the Roman Age group, and SI, NA, ME, GO, LO are from the Medieval Group. Samples from ME were collected directly at the archaeological site. B) Number of filtered reads (y-axis) per archaeological site (x-axis). C) Percentage of reads mapped to the human genome (y-axis) per archaeological site (x-axis).

Figure 2. Microorganism kingdoms detected in analyzed archaeological samples. A) Pie-plot representing overall frequency of microorganism kingdoms in archaeological samples; B) box and whiskers plot representing the distribution of frequencies of particular microorganism kingdoms in archaeological sites (GO not shown as includes only 2 samples); and C) stacked barplot indicating the frequency of microorganism kingdoms in a particular sample. Each bar represents an individual sample. Samples are ordered by the archeological sites. The color legend for all plots is shown at the bottom.

Figure 3. Bacterial and archaeal classes detected in analyzed archaeological samples. A) Pie-plot representing overall frequency of bacterial and archaeal classes in archaeological samples; B) box and whiskers plot representing distribution of frequencies of 6 most abundant bacterial classes (present in at least 1%) in archaeological sites (GO not shown as includes only 2 samples); and C) stacked barplot indicating the frequency of bacterial and archaeal classes in a particular sample. Each stacked bar represents an individual sample. Samples are ordered by the archeological sites. The color legend for all plots is shown at the bottom.

Figure 4. Bacterial and archaeal types [environmental (light green), oral (blue), other (yellow) and pathogenic (red)] detected in analyzed archaeological samples. A) Pie-plot representing overall frequency of bacterial and archaeal types in archaeological samples; B) box and whiskers plot representing distribution of frequencies of bacterial and archaeal types in archaeological sites (GO not shown as includes only 2 samples); and C) stacked barplot indicating the frequency of bacterial and archaeal types in a particular sample. Each stacked bar represents an individual sample. Samples are ordered by the archeological sites. The color legend for all plots is shown at the bottom.

Figure 5. Principal coordinate analysis of microbial compositions at four taxonomic levels: A) class, B) family, C) genus, and D) species. Samples from certain archaeological sites are marked in different colors and labeled with archaeological site ID.

Figure 6. A) Comparison of bacterial and archaeal profiles (stacked barplot) on class level based on

shallow and deep sequencing of the selected 11 samples (Sample ID is indicated on the x-axis, the first bar in a pair is shallow and the second is deep sequencing). The correlation coefficient R is placed above each shallow/deep stacked bar pair. The color legend is the same as in Figure 3. B) Correlation R values (y-axies) for shallow and deep sequencing pairs on different taxonomic levels (C – class, F – family, G –genus, S – species).

Figure 7. The DNA damage in samples with different fractions of environmental bacteria/archaea. Barplots indicating deamination rate in single stranded DNA overhangs (δ_s) and double stranded DNA fragments (δ_d) in microbial (left-hand site) and human DNA (right-hand site), grouped based on the fraction of environmental bacteria/archaea in sample and the length of single-stranded DNA overhangs (λ , expressed as:1/λ-1) calculated for 77 representative bacteria/archaea and endogenous human aDNA. Samples were grouped based on the fraction of environmental bacteria/archaea in a sample (0–25%, 25–50%, 50–75% and 75–100%).

Figure 8. The differences of DNA damage levels [$\Delta\delta_s$, $\Delta\delta_d$, Δλ , expressed as: Δ(1/λ-1)] of bacteria/archaea species belonging to the 5 groups (environmental, all human-related, oral, gut and pathogen) in comparison to damage levels in human aDNA. Boxes, whiskers and dots represent the distribution of differences in DNA damage levels of particular bacterial/archaeal groups. Each dot represents the difference in an individual sample. The color legend is the same as in Figure 4 (All human-related species are in orange).

Figure 9. DNA damage level (δ_s, δ_d, 1/λ-1) in environmental and all human-related *Actinobacteria* species. Boxes, whiskers and dots represent the distribution of DNA damage levels in particular samples. The color legend is the same as in Figure 4 (All human-related species are in orange).

Figure 1

Click here to download Figure Figure_1.pdf

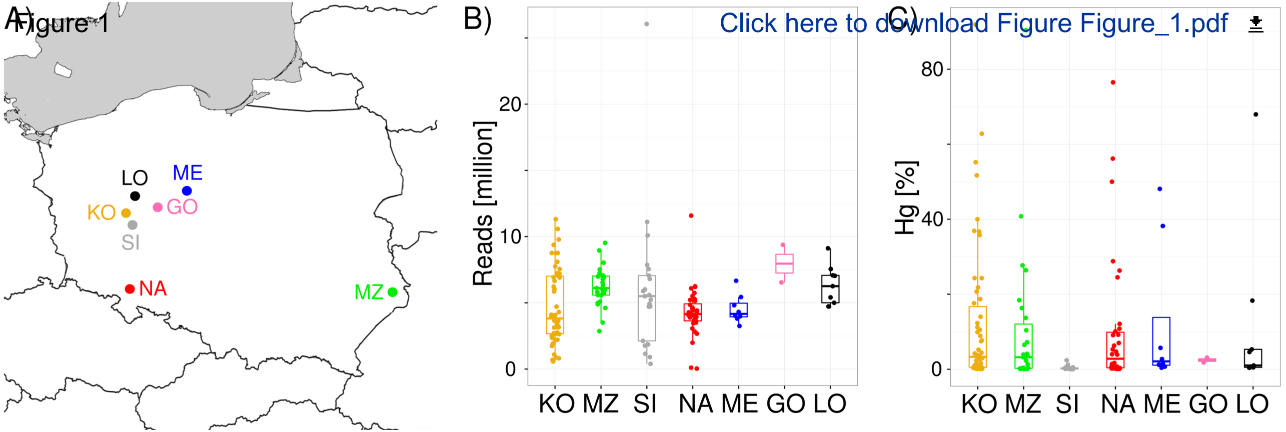

Figure 2

Click here to download Figure Figure_2.pdf

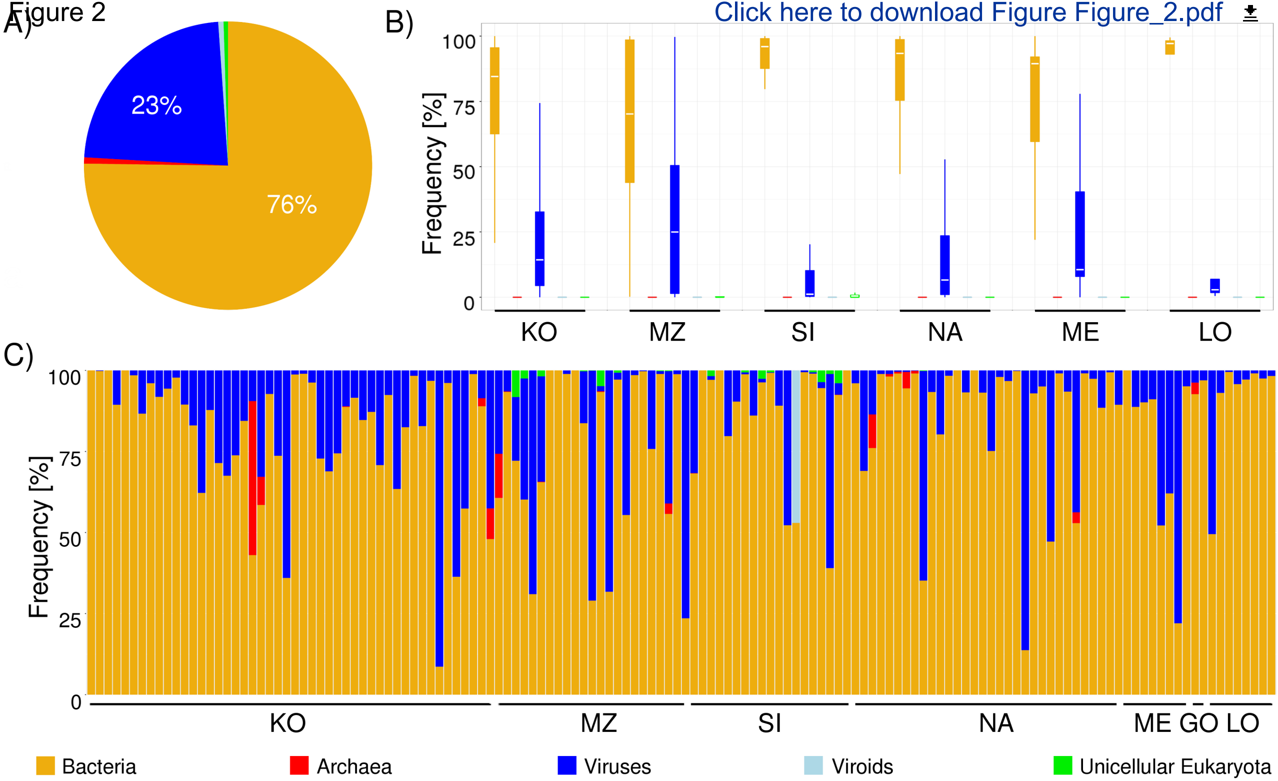

Figure 3

Click here to download Figure Figure_3.pdf

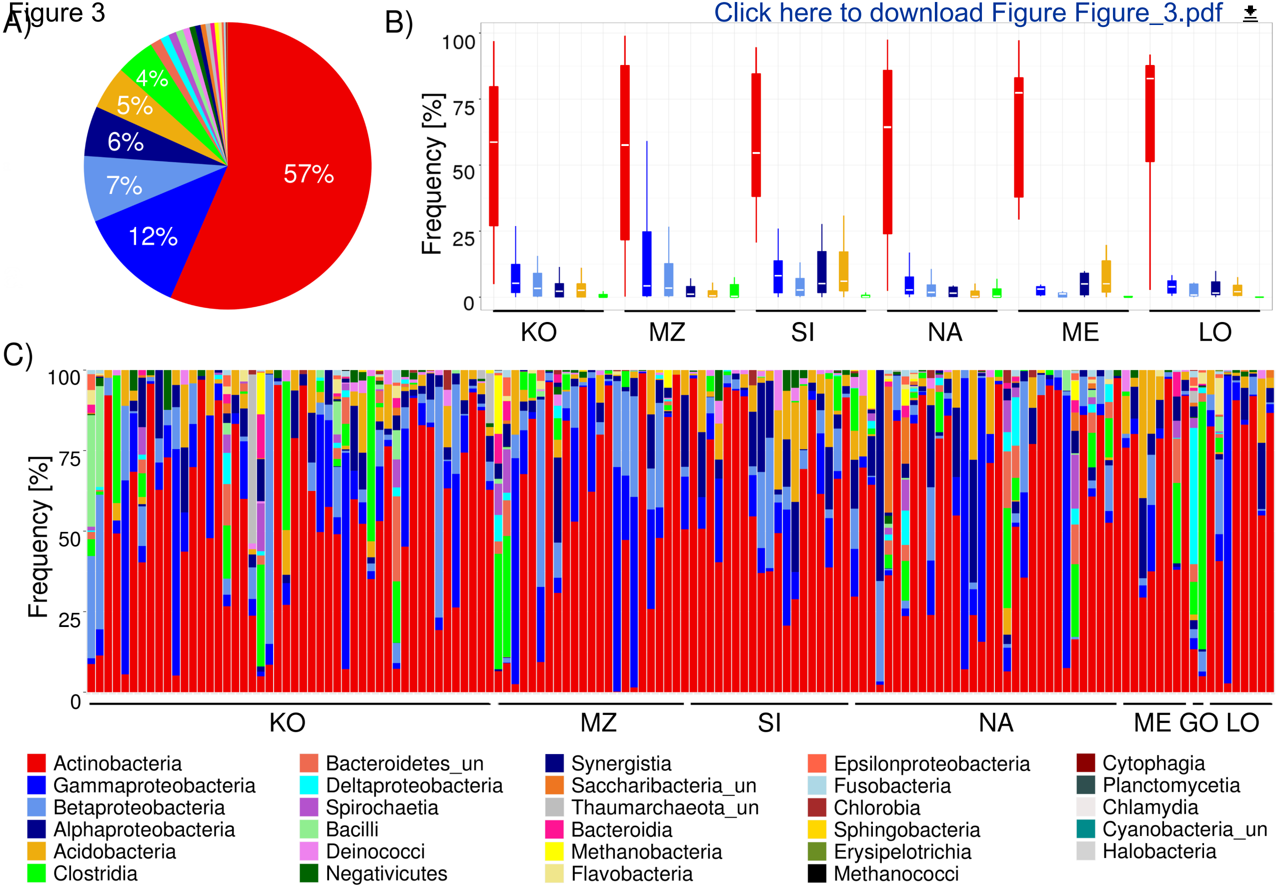

Figure 4

Click here to download Figure Figure_4.pdf

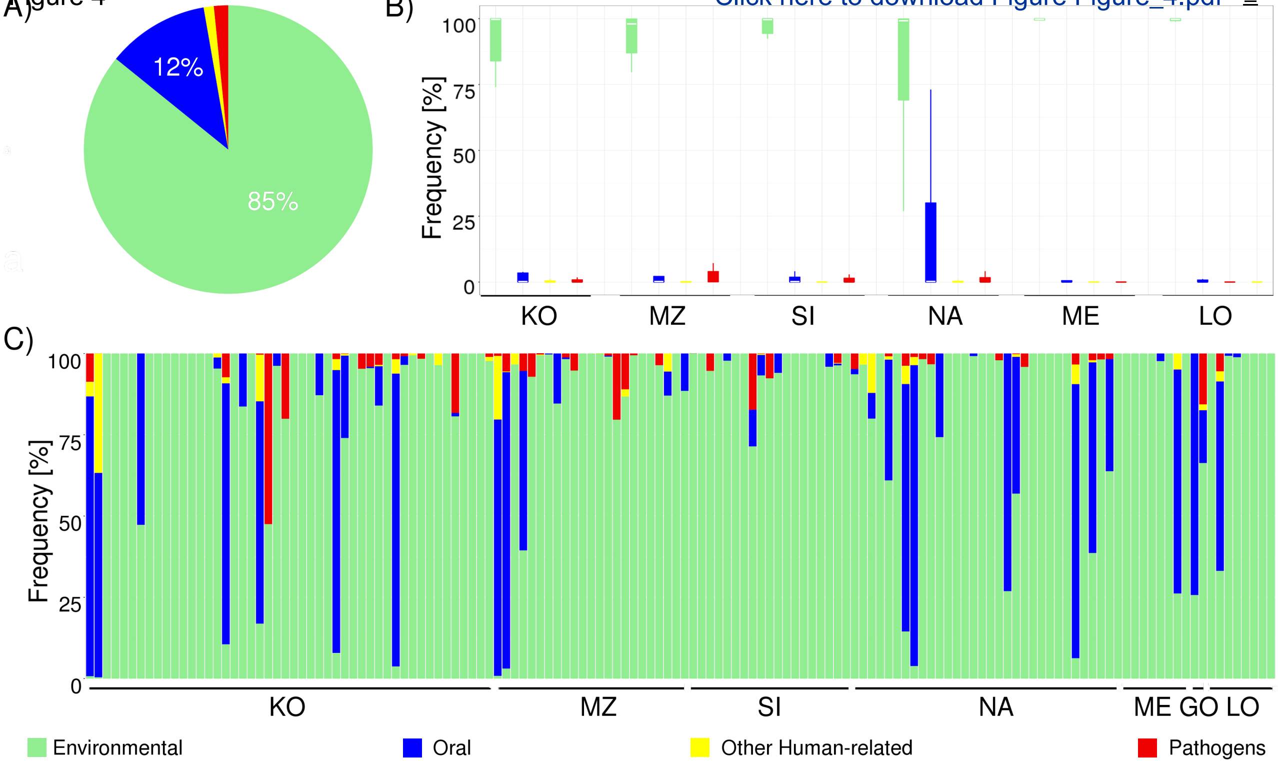

Figure 5

Click here to download Figure Figure_5.pdf

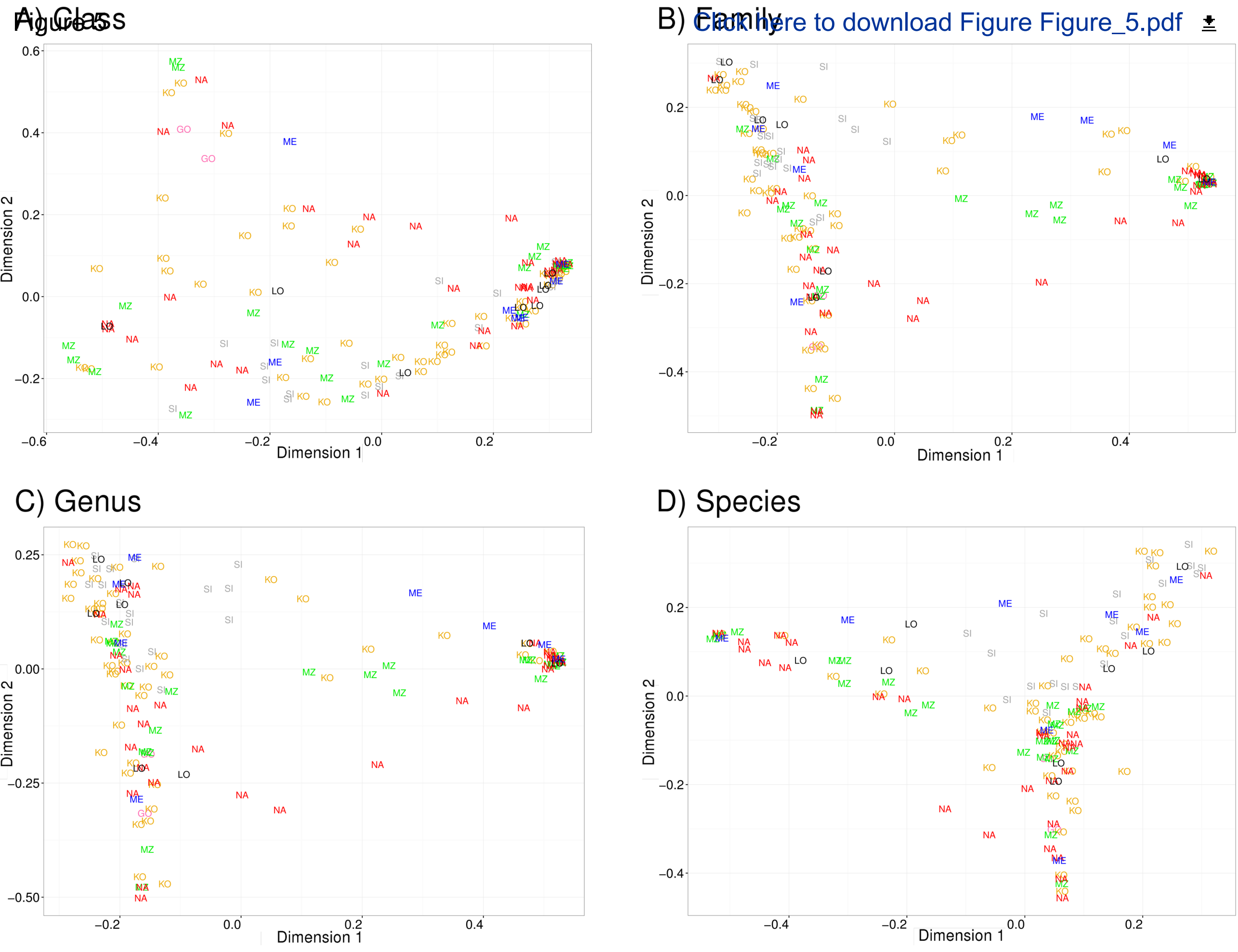

Figure 6 Click here to download Figure Figure_6.pdf

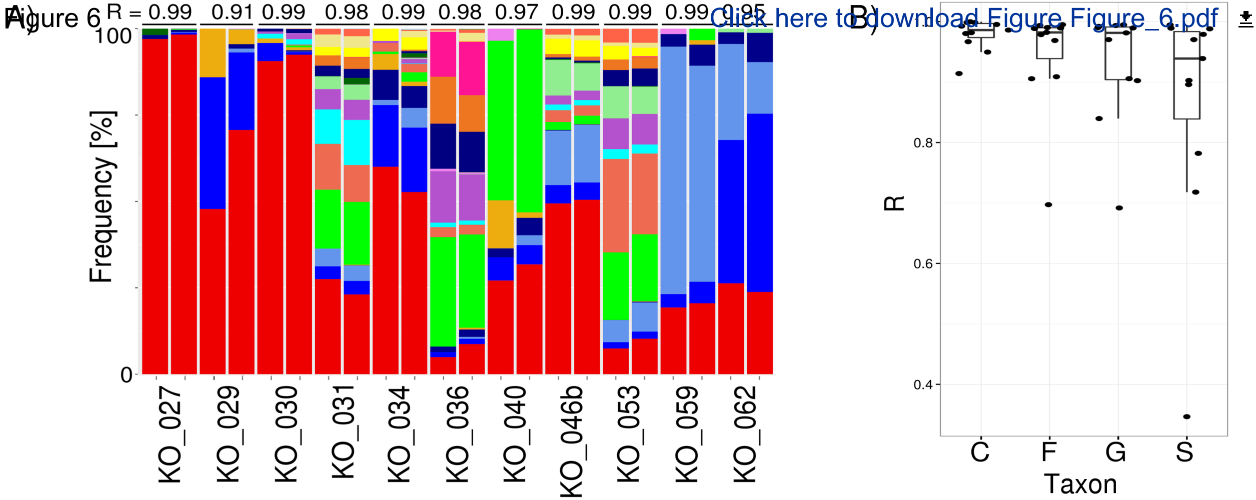

Figure 7

Click here to download Figure Figure_7.pdf

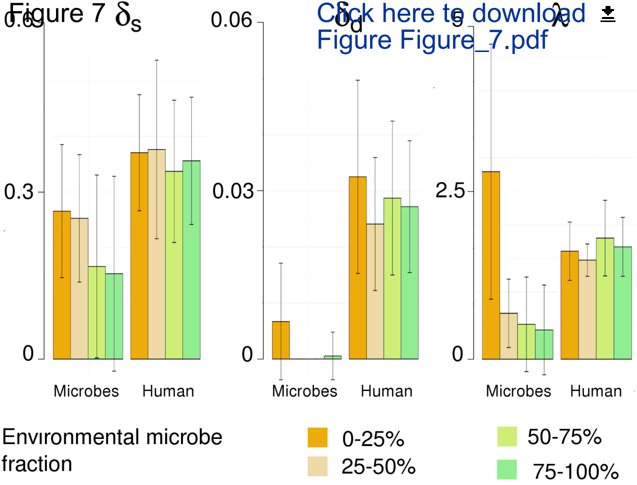

Figure 8

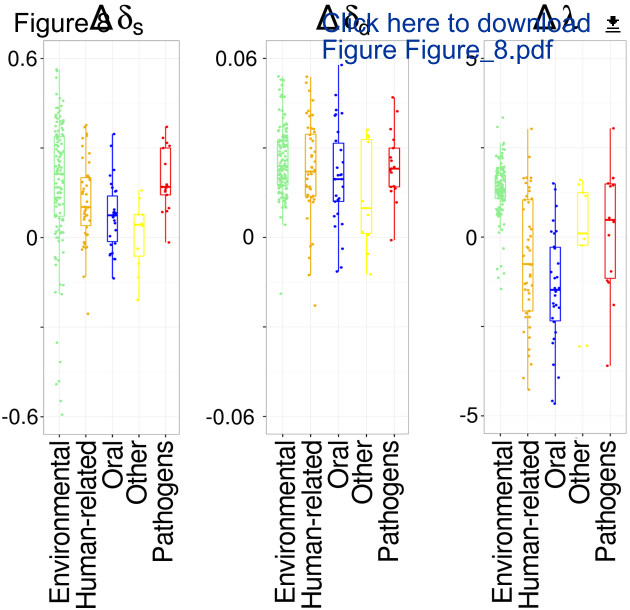

Click here to download Figure Figure_8.pdf

Figure 9

Click here to download Figure Figure_9.pdf

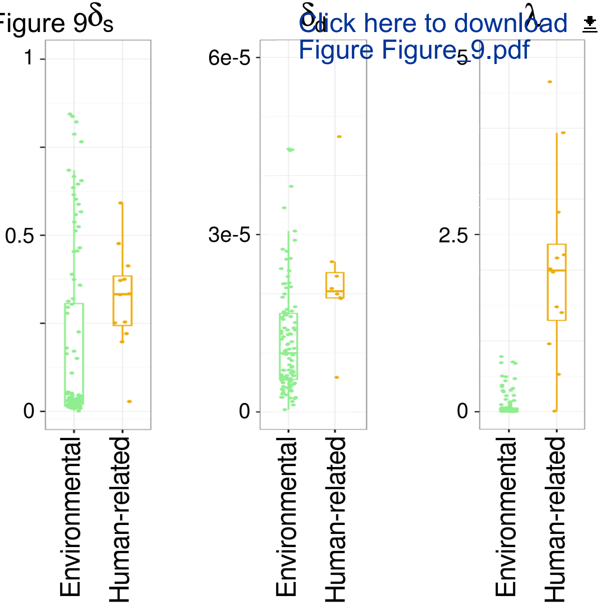

Supplementary Tables

Click here to access/download
Supplementary Material
aDNA_microorganisms_Figlerowicz_Supplementary_Tables.xlsx

Supplementary Tables Legends

Click here to access/download
Supplementary Material
aDNA_microorganisms_Figlerowicz_Supplementary_Tables_leg.docx

Supplementary Figures

Click here to access/download
Supplementary Material
aDNA_microorganisms_Figlerowicz_Supplementary_Figures.pdf
