# Supplementary material for: Comprehensive analysis of microorganisms accompanying human archaeological remains
Source: Gigascience. 2017 Jun 13;6(7):1–13. doi: 10.1093/gigascience/gix044 (PMC5965364; doi:10.1093/gigascience/gix044)
Supplement: aDNA_microorganisms_Figlerowicz_Supplementary_Figures.pdf [file gix044_adna_microorganisms_figlerowicz_supplementary_figures.pdf]

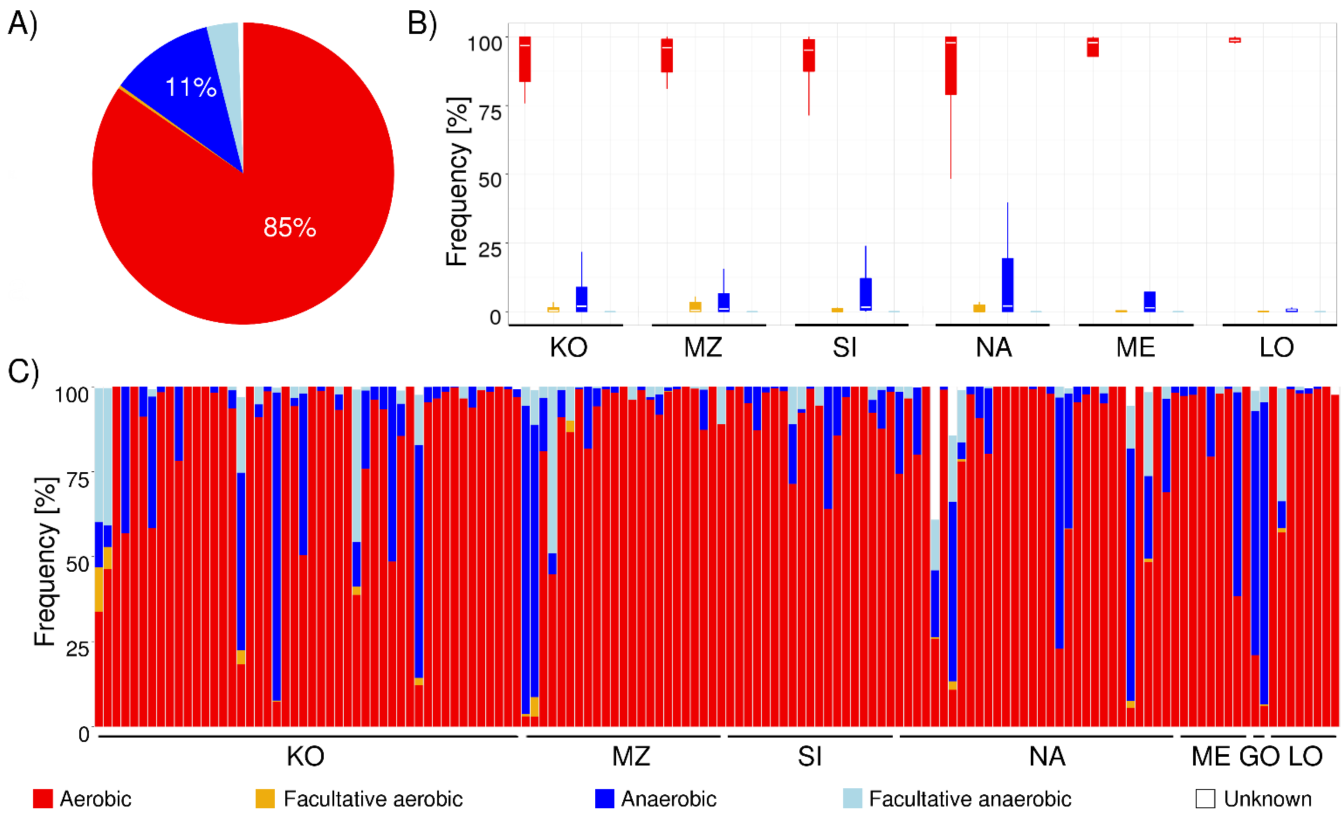

Supplementary Figure 1. Bacterial/archaeal respiratory types detected in analyzed archaeological samples. A) Pie-plot representing overall frequency bacterial/archaeal respiratory types in archaeological samples; B) box and whiskers plot representing the distribution of frequencies of particular bacterial/archaeal respiratory type in archaeological sites (GO not shown as includes only 2 samples); and C) stacked barplot indicating the frequency of bacterial/archaeal respiratory types in a particular sample. Each bar represents an individual sample. Samples are ordered by the archeological sites. The color legend for all plots is shown at the bottom.

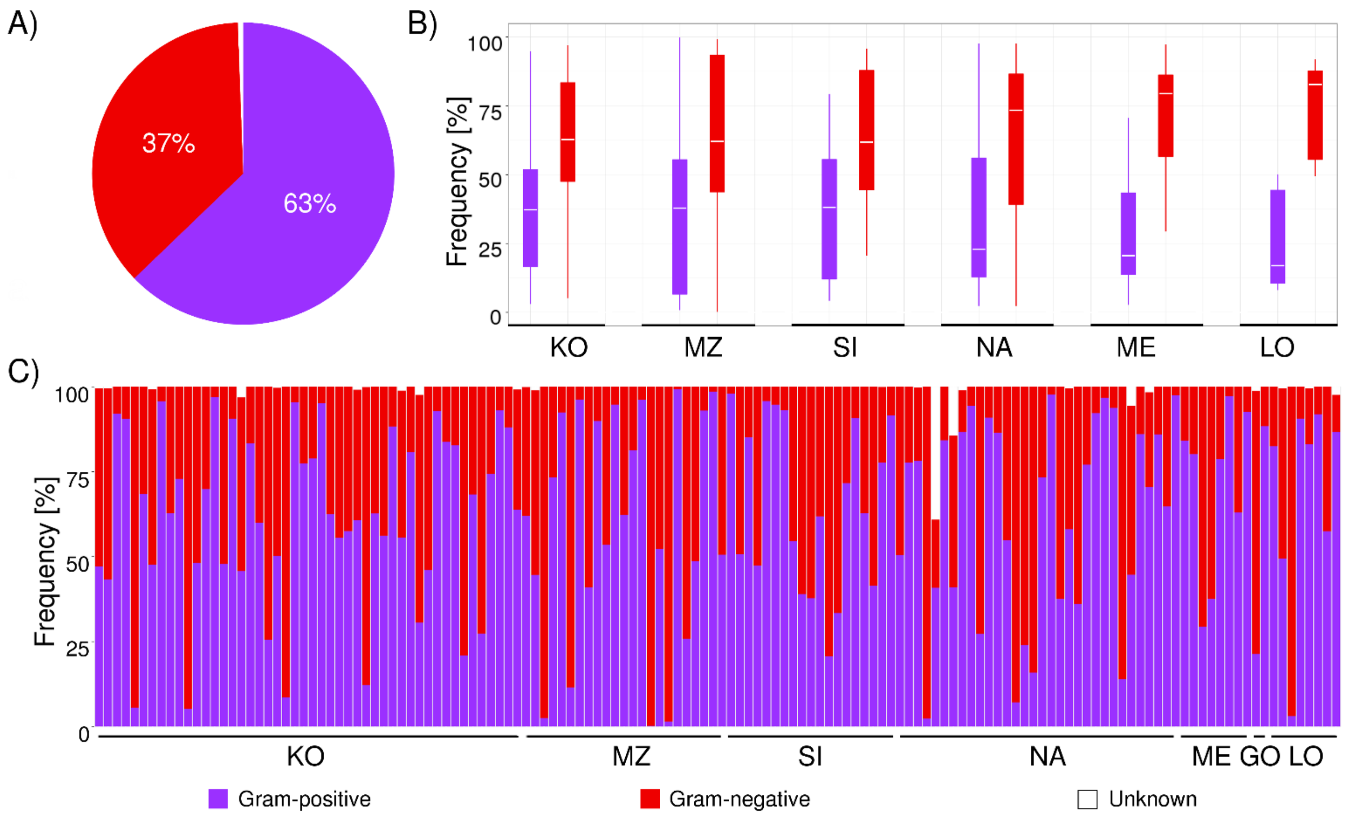

Supplementary Figure 2. Bacterial/archaeal gram stain types detected in analyzed archaeological samples. A) Pie-plot representing overall frequency bacterial/archaeal gram stain types in archaeological samples; B) box and whiskers plot representing the distribution of frequencies of particular bacterial/archaeal gram stain type in archaeological sites (GO not shown as includes only 2 samples); and C) stacked barplot indicating the frequency of bacterial/archaeal gram stain types in a particular sample. Each bar represents an individual sample. Samples are ordered by the archeological sites. The color legend for all plots is shown at the bottom.

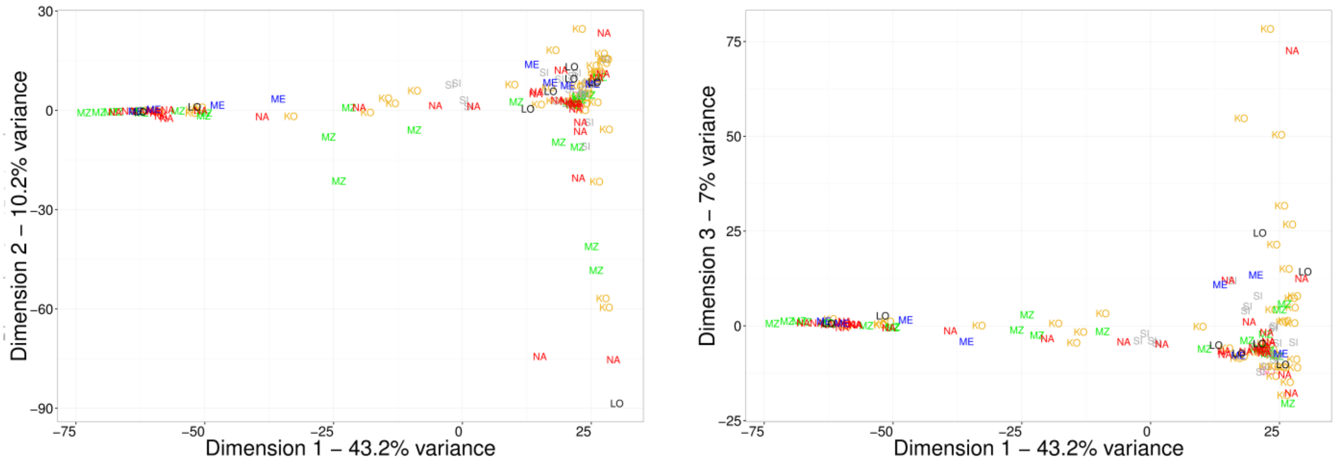

Supplementary Figure 3. Principal component analysis (PCA) of microbial compositions at genus taxonomic level. Left-hand side: Principal components 1 and 2. Right-hand side: Principal components 1 and 3. Samples from certain archaeological sites are marked in different colors and labelled with archaeological site ID.

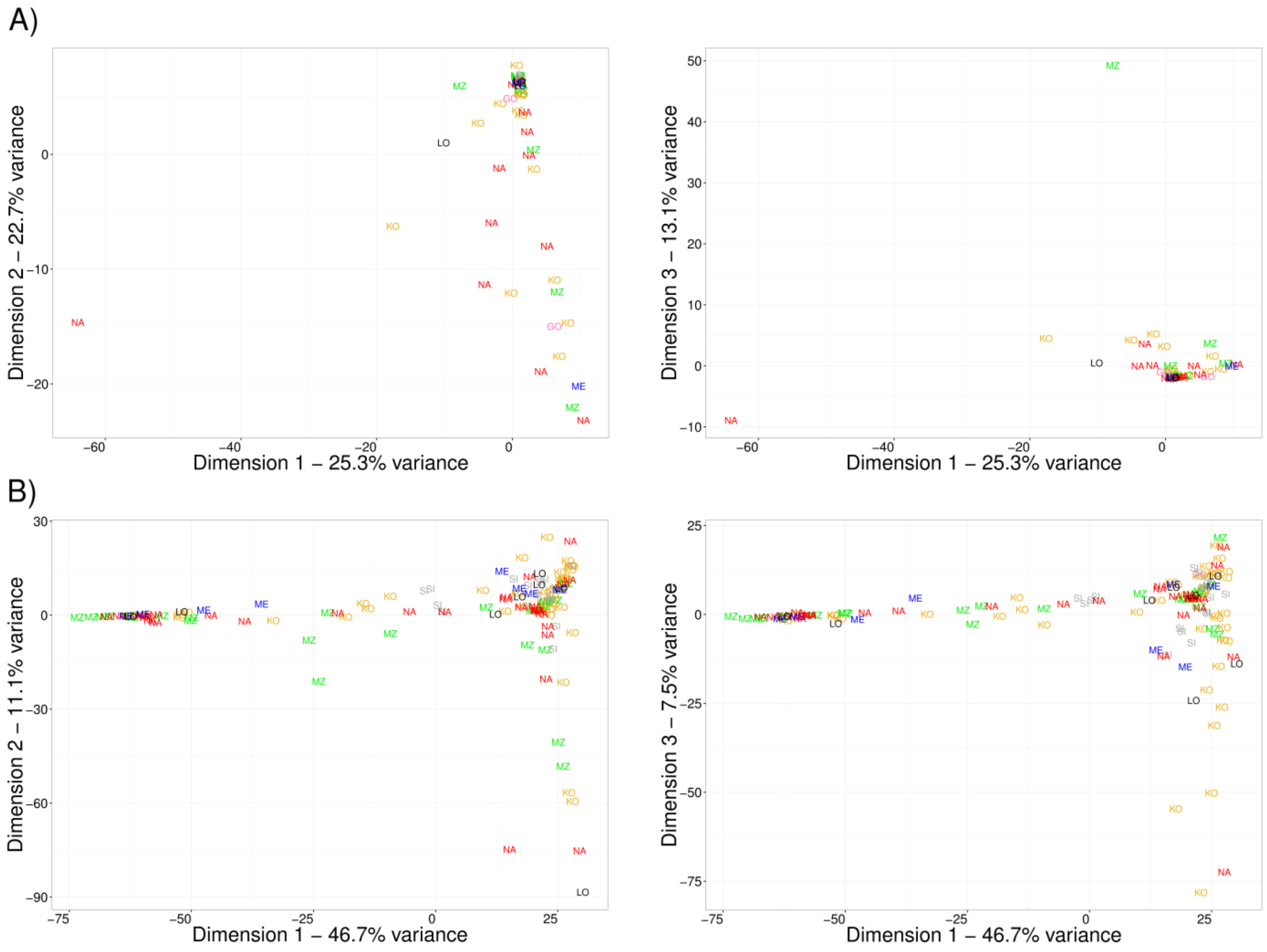

Supplementary Figure 4. Principal component analysis (PCA) of microbial compositions at genus taxonomic level: A) Human-related genera B) Environmental genera. Left-hand side: Principal components 1 and 2. Right-hand side: Principal components 1 and 3. Samples from certain archaeological sites are marked in different colors and labelled with archaeological site ID.

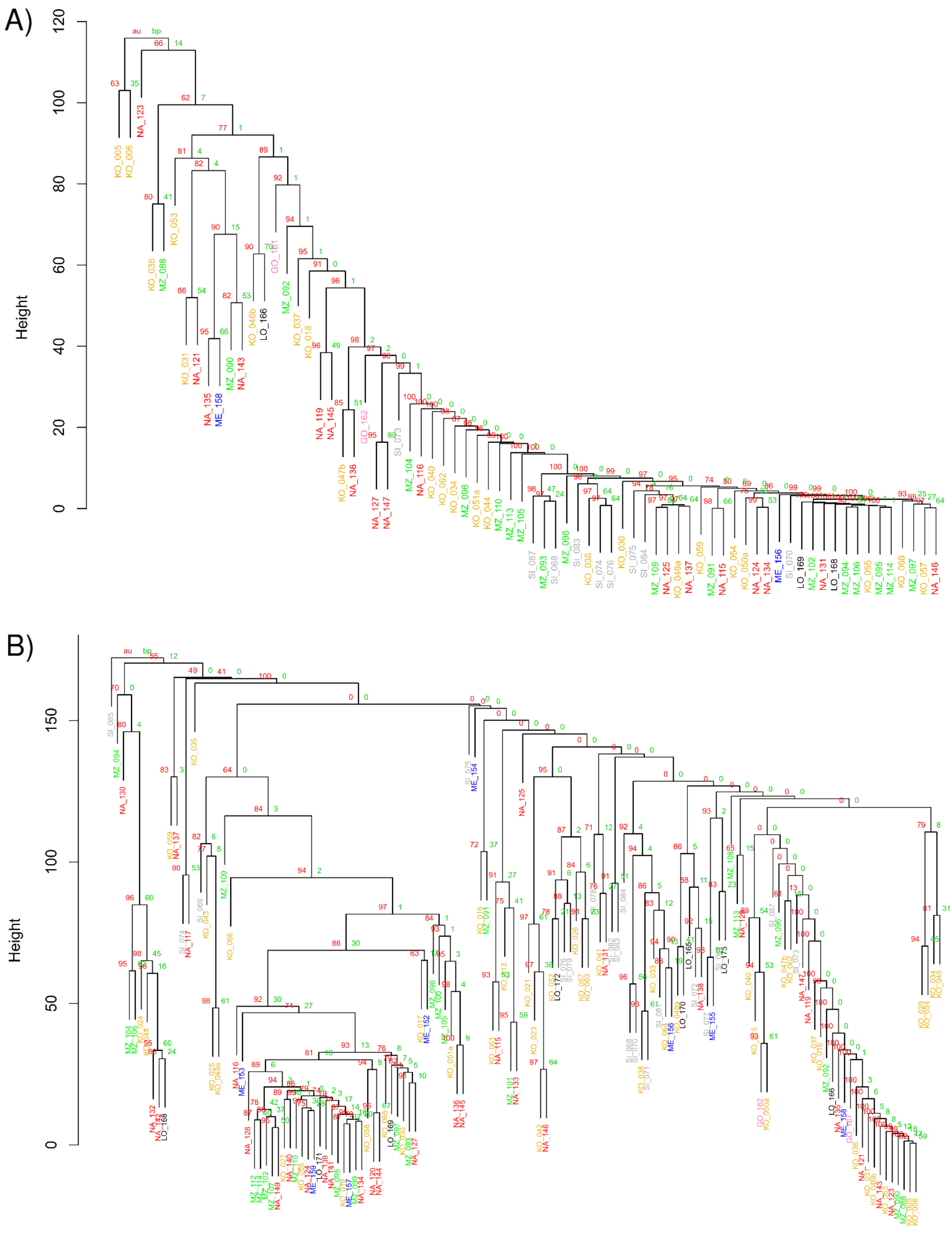

Supplementary Figure 5. Hierarchical clustering of samples (Manhattan distances, cluster method: average) at the genus taxonomic level A) Human-related genera B) Environmental genera. Samples from certain archaeological sites are marked in different colors and labelled with archaeological site ID.

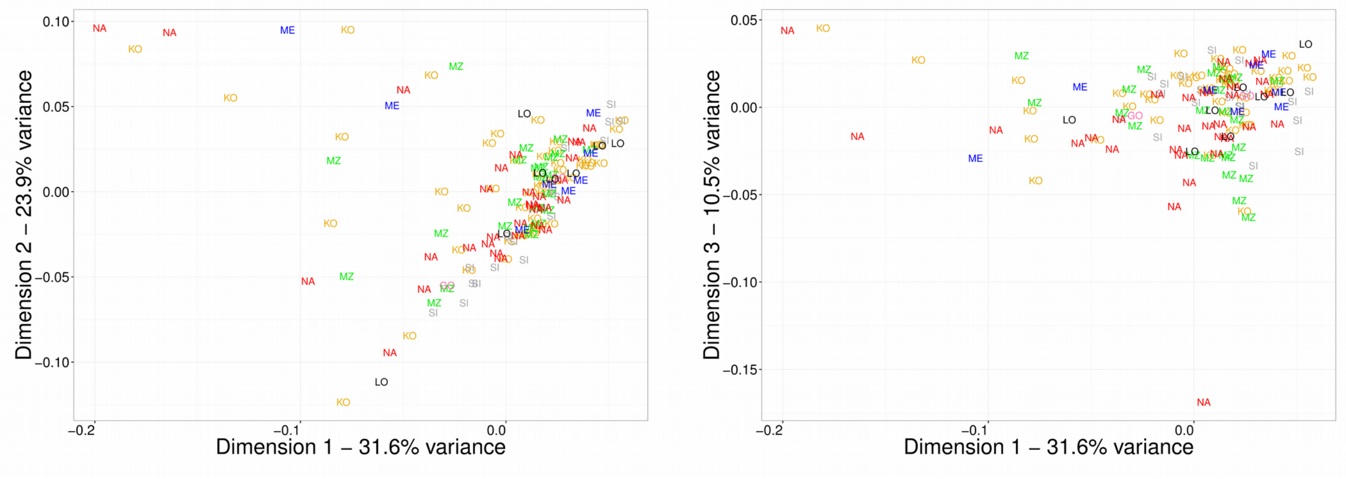

Supplementary Figure 6. Principal component analysis (PCA) of exogenous 10-mer reads. Left-hand side: Principal components 1 and 2. Right-hand side: Principal components 1 and 3.

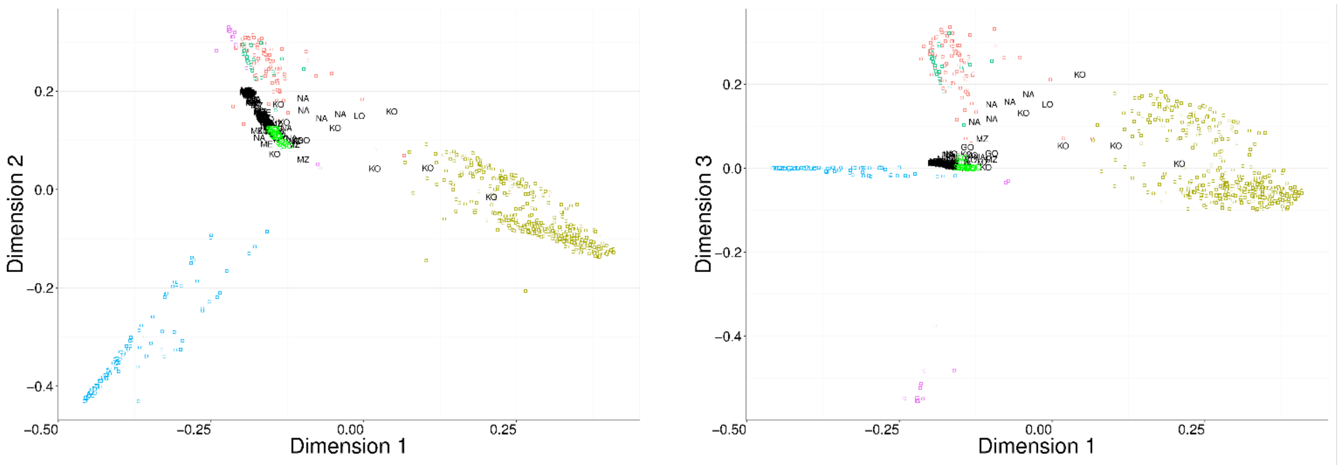

Supplementary Figure 7. Principal coordinate analysis (PCoA) of microbial compositions at genus taxonomic level in various human associated microbiomes (mouth – brown squares, nose – red squares, skin – green squares, vagina – purple squares, stool – blue squares), soils (light green circles) and ancient human remains (black labels). Left-hand side: Principal components 1 and 2. Right-hand side: Principal components 1 and 3.

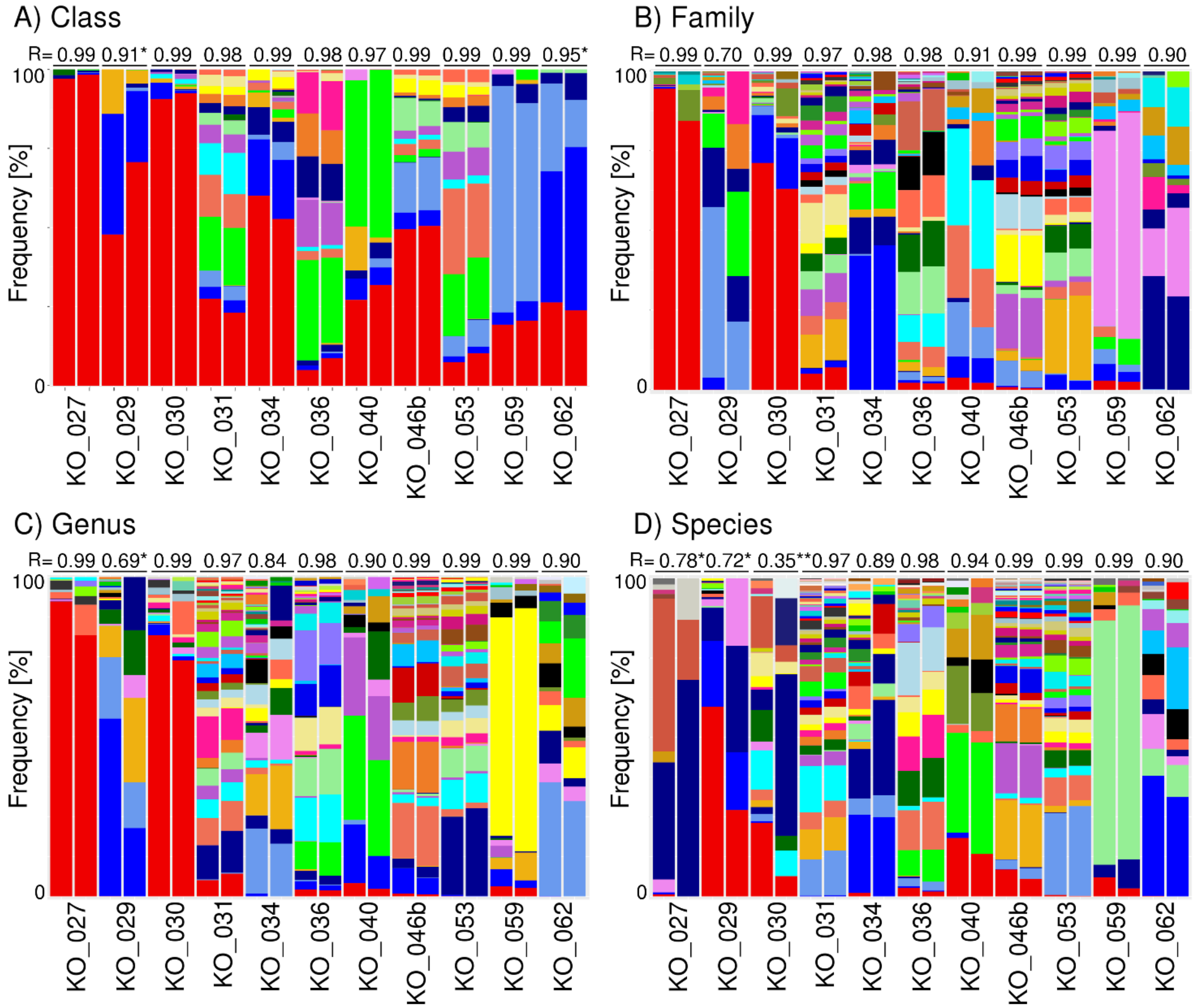

Supplementary Figure 8. Comparison of bacterial and archaeal profiles (stacked barplot) on A) class B) family C) genus D) species levels based on shallow and deep sequencing of the selected 11 samples (Sample ID is indicated on the x-axis). The correlation coefficient R is placed above each shallow/deep stacked bar pair. The bar colors represent different classes, families, genera or species, respectively. * 0.01>p>0.001; ** p=0.09360; nothing: p<0.0001.

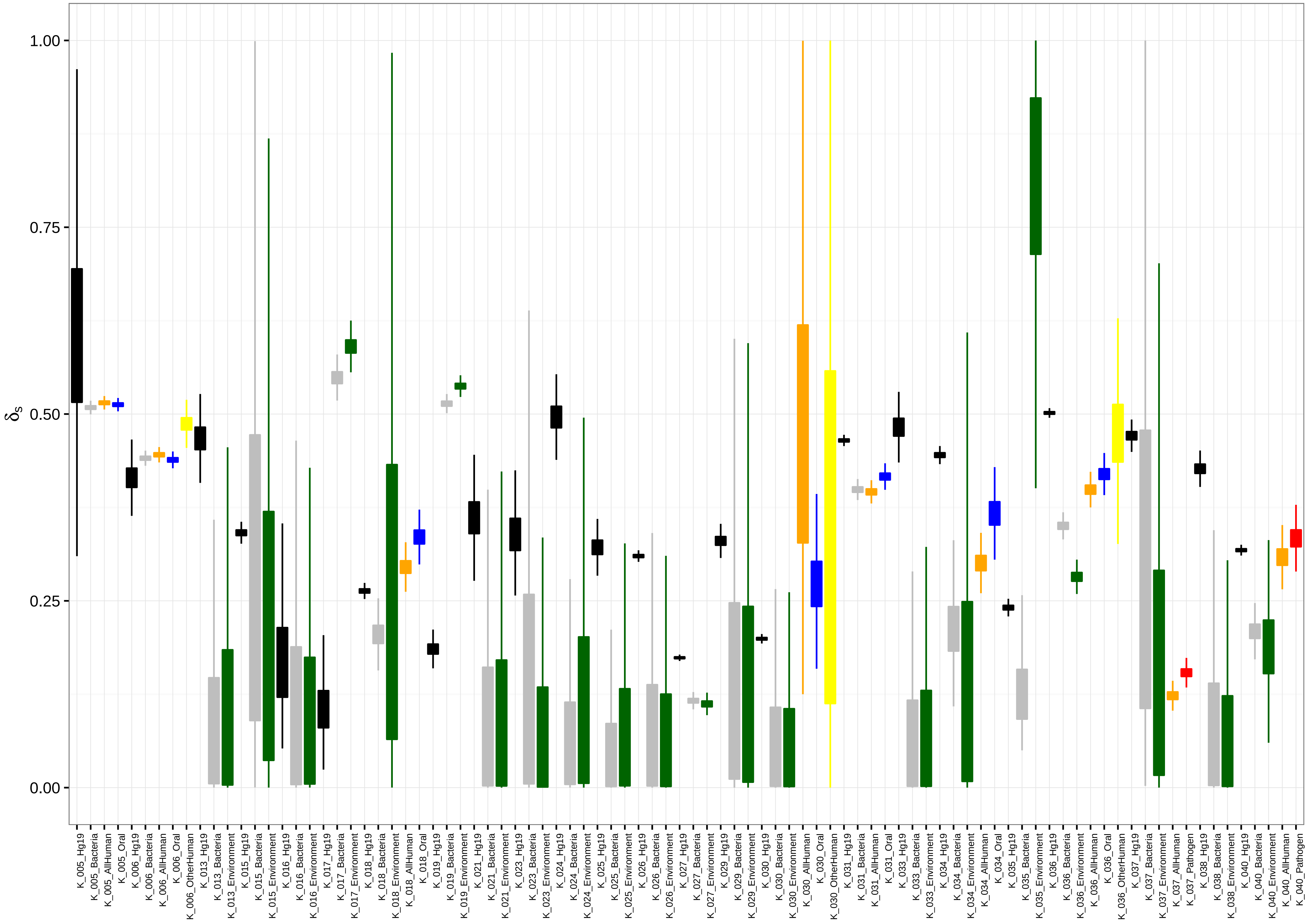

δs
1.00
0.75
0.50
0.25
0.00
K_005_Hg19
K_005_Bacteria
K_005_AllHuman
K_005_Oral
K_006_Hg19
K_006_Bacteria
K_006_AllHuman
K_006_Oral
K_006_OtherHuman
K_013_Hg19
K_013_Bacteria
K_013_Environment
K_015_Hg19
K_015_Bacteria
K_015_Environment
K_016_Hg19
K_016_Bacteria
K_016_Environment
K_017_Hg19
K_017_Bacteria
K_017_Environment
K_018_Hg19
K_018_Bacteria
K_018_Environment
K_018_AllHuman
K_018_Oral
K_019_Hg19
K_019_Bacteria
K_019_Environment
K_021_Hg19
K_021_Bacteria
K_021_Environment
K_023_Hg19
K_023_Bacteria
K_023_Environment
K_024_Hg19
K_024_Bacteria
K_024_Environment
K_025_Hg19
K_025_Bacteria
K_025_Environment
K_026_Hg19
K_026_Bacteria
K_026_Environment
K_027_Hg19
K_027_Bacteria
K_027_Environment
K_029_Hg19
K_029_Bacteria
K_029_Environment
K_030_Hg19
K_030_Bacteria
K_030_Environment
K_030_AllHuman
K_030_Oral
K_030_OtherHuman
K_031_Hg19
K_031_Bacteria
K_031_AllHuman
K_031_Oral
K_033_Hg19
K_033_Bacteria
K_033_Environment
K_034_Hg19
K_034_Bacteria
K_034_Environment
K_034_AllHuman
K_034_Oral
K_035_Hg19
K_035_Bacteria
K_035_Environment
K_036_Hg19
K_036_Bacteria
K_036_Environment
K_036_AllHuman
K_036_Oral
K_036_OtherHuman
K_037_Hg19
K_037_Bacteria
K_037_Environment
K_037_AllHuman
K_037_Pathogen
K_038_Hg19
K_038_Bacteria
K_038_Environment
K_040_Hg19
K_040_Bacteria
K_040_Environment
K_040_AllHuman
K_040_Pathogen

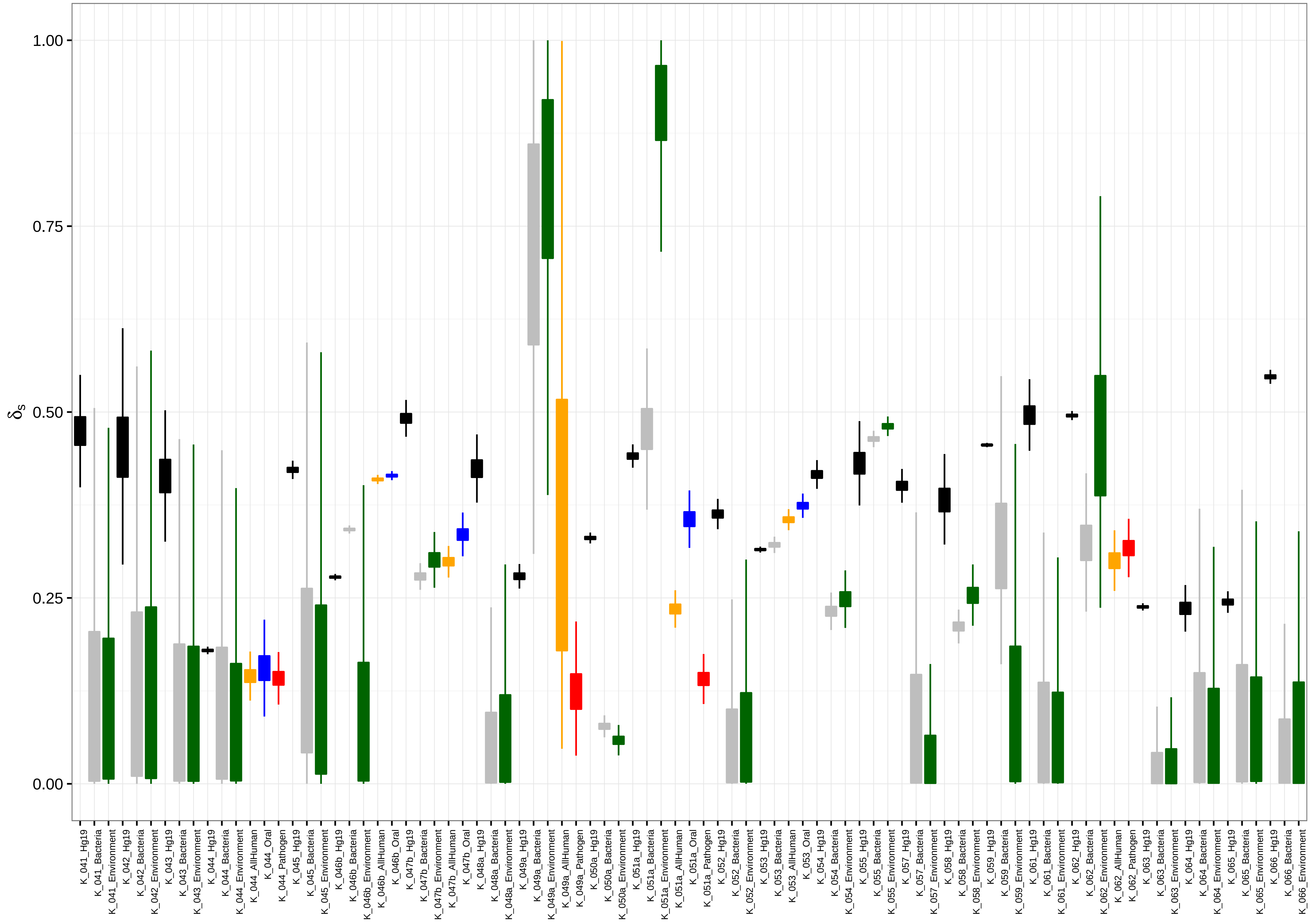

1.00
0.75
0.50
0.25
0.00
δs
K_041_Hg19
K_041_Bacteria
K_041_Environment
K_042_Hg19
K_042_Bacteria
K_042_Environment
K_043_Hg19
K_043_Bacteria
K_043_Environment
K_044_Hg19
K_044_Bacteria
K_044_Environment
K_044_AllHuman
K_044_Oral
K_044_Pathogen
K_045_Hg19
K_045_Bacteria
K_045_Environment
K_046b_Hg19
K_046b_Bacteria
K_046b_Environment
K_046b_AllHuman
K_046b_Oral
K_047b_Hg19
K_047b_Bacteria
K_047b_Environment
K_047b_AllHuman
K_047b_Oral
K_048a_Hg19
K_048a_Bacteria
K_048a_Environment
K_049a_Hg19
K_049a_Bacteria
K_049a_Environment
K_049a_AllHuman
K_049a_Pathogen
K_050a_Hg19
K_050a_Bacteria
K_050a_Environment
K_051a_Hg19
K_051a_Bacteria
K_051a_Environment
K_051a_AllHuman
K_051a_Oral
K_051a_Pathogen
K_052_Hg19
K_052_Bacteria
K_052_Environment
K_053_Hg19
K_053_Bacteria
K_053_AllHuman
K_053_Oral
K_054_Hg19
K_054_Bacteria
K_054_Environment
K_055_Hg19
K_055_Bacteria
K_055_Environment
K_057_Hg19
K_057_Bacteria
K_057_Environment
K_058_Hg19
K_058_Bacteria
K_058_Environment
K_059_Hg19
K_059_Bacteria
K_059_Environment
K_061_Hg19
K_061_Bacteria
K_061_Environment
K_062_Hg19
K_062_Bacteria
K_062_Environment
K_062_AllHuman
K_062_Pathogen
K_063_Hg19
K_063_Bacteria
K_063_Environment
K_064_Hg19
K_064_Bacteria
K_064_Environment
K_065_Hg19
K_065_Bacteria
K_065_Environment
K_066_Hg19
K_066_Bacteria
K_066_Environment

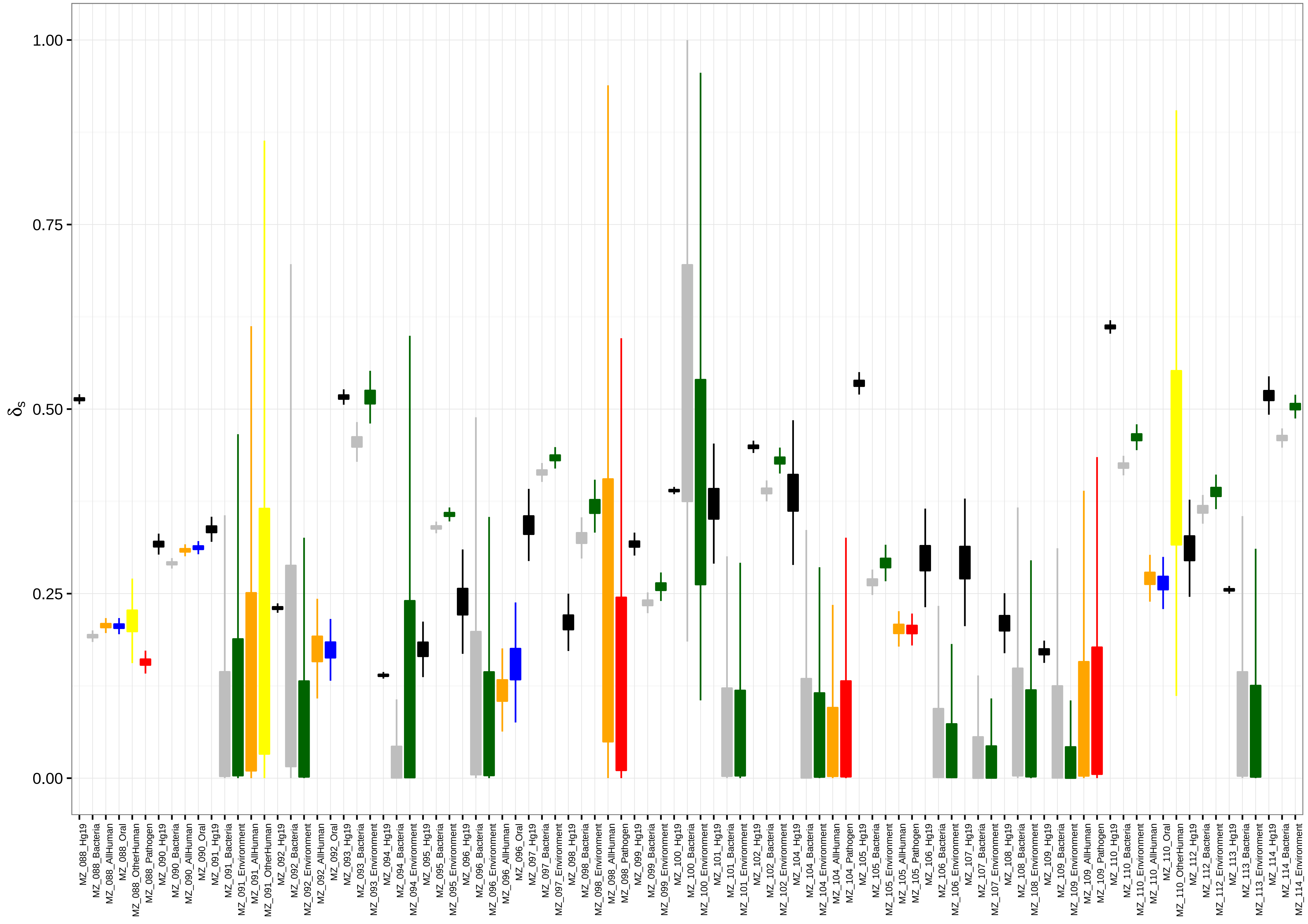
1.00
0.75
0.50
0.25
0.00
δs
MZ_088_Hg19
MZ_088_Bacteria
MZ_088_AllHuman
MZ_088_Oral
MZ_088_OtherHuman
MZ_088_Pathogen
MZ_090_Hg19
MZ_090_Bacteria
MZ_090_AllHuman
MZ_090_Oral
MZ_091_Hg19
MZ_091_Bacteria
MZ_091_Environment
MZ_091_AllHuman
MZ_091_OtherHuman
MZ_092_Hg19
MZ_092_Bacteria
MZ_092_Environment
MZ_092_AllHuman
MZ_092_Oral
MZ_093_Hg19
MZ_093_Bacteria
MZ_093_Environment
MZ_094_Hg19
MZ_094_Bacteria
MZ_094_Environment
MZ_095_Hg19
MZ_095_Bacteria
MZ_095_Environment
MZ_096_Hg19
MZ_096_Bacteria
MZ_096_Environment
MZ_096_AllHuman
MZ_096_Oral
MZ_097_Hg19
MZ_097_Bacteria
MZ_097_Environment
MZ_098_Hg19
MZ_098_Bacteria
MZ_098_Environment
MZ_098_AllHuman
MZ_098_Pathogen
MZ_099_Hg19
MZ_099_Bacteria
MZ_099_Environment
MZ_100_Hg19
MZ_100_Bacteria
MZ_100_Environment
MZ_101_Hg19
MZ_101_Bacteria
MZ_101_Environment
MZ_102_Hg19
MZ_102_Bacteria
MZ_102_Environment
MZ_104_Hg19
MZ_104_Bacteria
MZ_104_Environment
MZ_104_AllHuman
MZ_104_Pathogen
MZ_105_Hg19
MZ_105_Bacteria
MZ_105_Environment
MZ_105_AllHuman
MZ_105_Pathogen
MZ_106_Hg19
MZ_106_Bacteria
MZ_106_Environment
MZ_107_Hg19
MZ_107_Bacteria
MZ_107_Environment
MZ_108_Hg19
MZ_108_Bacteria
MZ_108_Environment
MZ_109_Hg19
MZ_109_Bacteria
MZ_109_Environment
MZ_109_AllHuman
MZ_109_Pathogen
MZ_110_Hg19
MZ_110_Bacteria
MZ_110_Environment
MZ_110_AllHuman
MZ_110_Oral
MZ_110_OtherHuman
MZ_112_Hg19
MZ_112_Bacteria
MZ_112_Environment
MZ_113_Hg19
MZ_113_Bacteria
MZ_113_Environment
MZ_114_Hg19
MZ_114_Bacteria
MZ_114_Environment

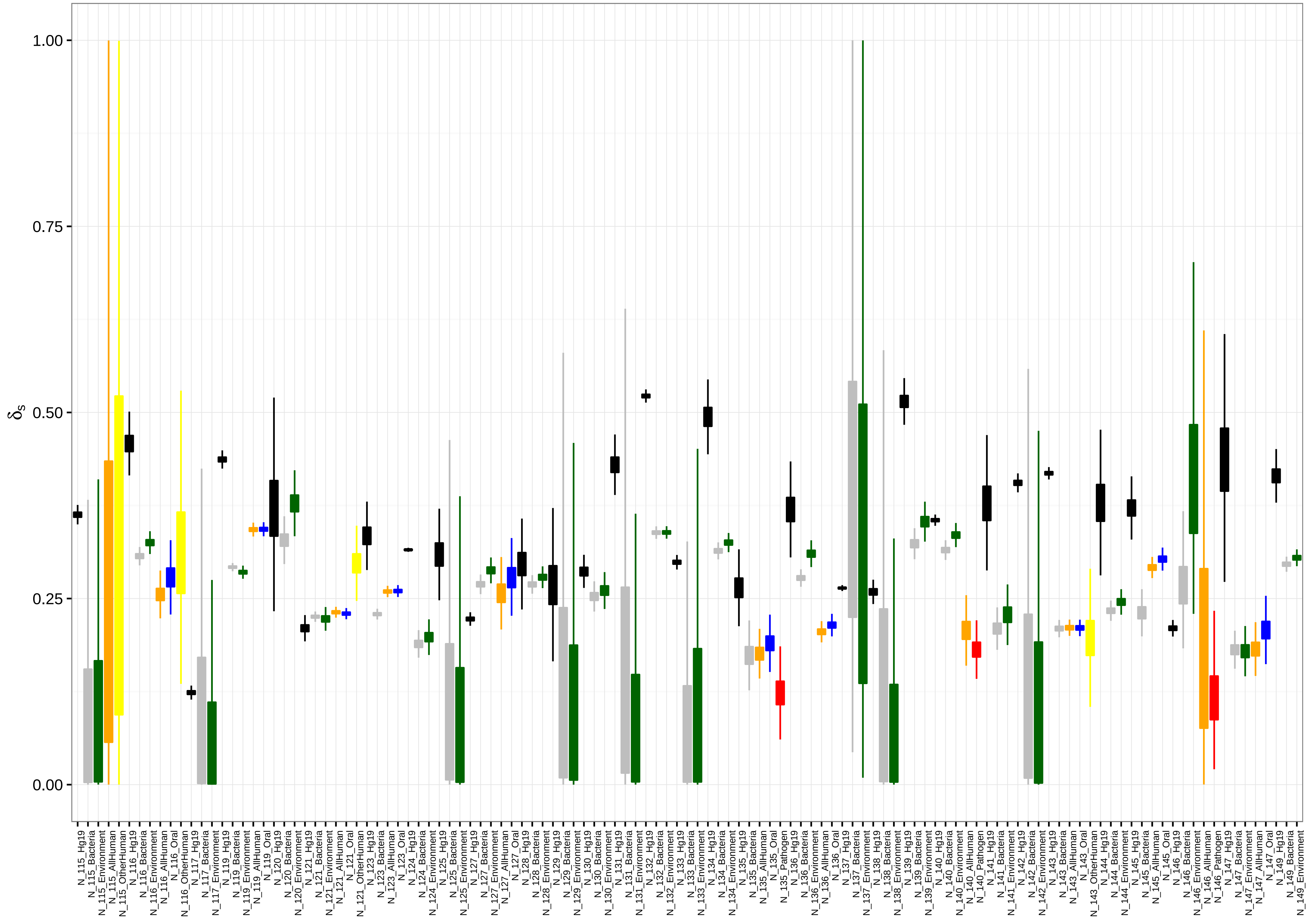

1.00
0.75
0.50
0.25
0.00
δs
N_115_Hg19
N_115_Bacteria
N_115_Environment
N_115_AllHuman
N_115_OtherHuman
N_116_Hg19
N_116_Bacteria
N_116_Environment
N_116_AllHuman
N_116_Oral
N_116_OtherHuman
N_117_Hg19
N_117_Bacteria
N_117_Environment
N_119_Hg19
N_119_Bacteria
N_119_Environment
N_119_AllHuman
N_119_Oral
N_120_Hg19
N_120_Bacteria
N_120_Environment
N_121_Hg19
N_121_Bacteria
N_121_Environment
N_121_AllHuman
N_121_Oral
N_121_OtherHuman
N_123_Hg19
N_123_Bacteria
N_123_AllHuman
N_123_Oral
N_124_Hg19
N_124_Bacteria
N_124_Environment
N_125_Hg19
N_125_Bacteria
N_125_Environment
N_127_Hg19
N_127_Bacteria
N_127_Environment
N_127_AllHuman
N_127_Oral
N_128_Hg19
N_128_Bacteria
N_128_Environment
N_129_Hg19
N_129_Bacteria
N_129_Environment
N_130_Hg19
N_130_Bacteria
N_130_Environment
N_131_Hg19
N_131_Bacteria
N_131_Environment
N_132_Hg19
N_132_Bacteria
N_132_Environment
N_133_Hg19
N_133_Bacteria
N_133_Environment
N_134_Hg19
N_134_Bacteria
N_134_Environment
N_135_Hg19
N_135_Bacteria
N_135_AllHuman
N_135_Oral
N_135_Pathogen
N_136_Hg19
N_136_Bacteria
N_136_Environment
N_136_AllHuman
N_136_Oral
N_137_Hg19
N_137_Bacteria
N_137_Environment
N_138_Hg19
N_138_Bacteria
N_138_Environment
N_139_Hg19
N_139_Bacteria
N_139_Environment
N_140_Hg19
N_140_Bacteria
N_140_Environment
N_140_AllHuman
N_140_Pathogen
N_141_Hg19
N_141_Bacteria
N_141_Environment
N_142_Hg19
N_142_Bacteria
N_142_Environment
N_143_Hg19
N_143_Bacteria
N_143_AllHuman
N_143_Oral
N_143_OtherHuman
N_144_Hg19
N_144_Bacteria
N_144_Environment
N_145_Hg19
N_145_Bacteria
N_145_AllHuman
N_145_Oral
N_146_Hg19
N_146_Bacteria
N_146_Environment
N_146_AllHuman
N_146_Pathogen
N_147_Hg19
N_147_Bacteria
N_147_Environment
N_147_AllHuman
N_147_Oral
N_149_Hg19
N_149_Bacteria
N_149_Environment

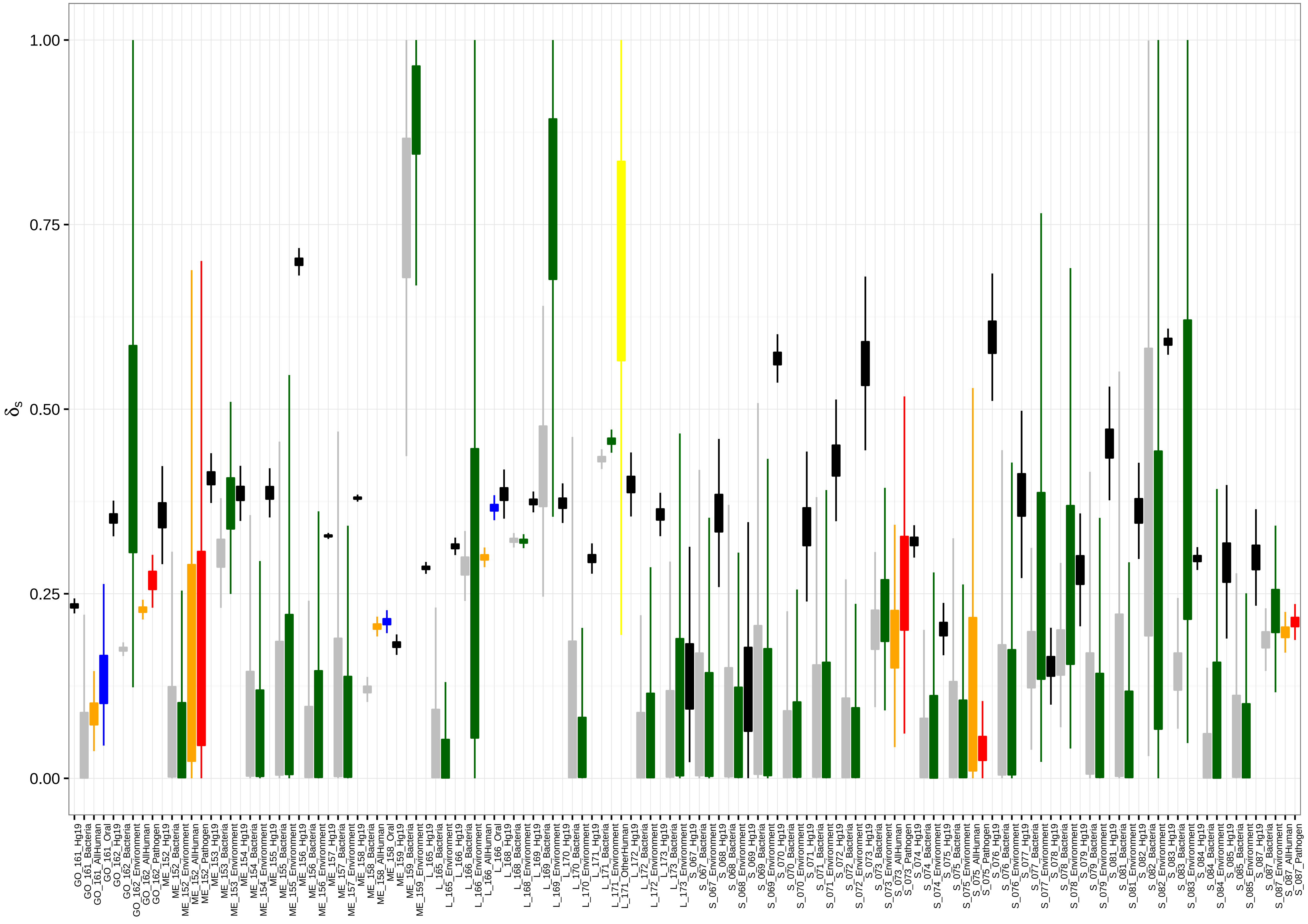
δs
1.00
0.75
0.50
0.25
0.00
GO_161_Hg19
GO_161_Bacteria
GO_161_AllHuman
GO_161_Oral
GO_162_Hg19
GO_162_Bacteria
GO_162_Environment
GO_162_AllHuman
GO_162_Pathogen
ME_152_Hg19
ME_152_Bacteria
ME_152_Environment
ME_152_AllHuman
ME_152_Pathogen
ME_153_Hg19
ME_153_Bacteria
ME_153_Environment
ME_154_Hg19
ME_154_Bacteria
ME_154_Environment
ME_155_Hg19
ME_155_Bacteria
ME_155_Environment
ME_156_Hg19
ME_156_Bacteria
ME_156_Environment
ME_157_Hg19
ME_157_Bacteria
ME_157_Environment
ME_158_Hg19
ME_158_Bacteria
ME_158_AllHuman
ME_158_Oral
ME_159_Hg19
ME_159_Bacteria
ME_159_Environment
L_165_Hg19
L_165_Bacteria
L_165_Environment
L_166_Hg19
L_166_Bacteria
L_166_Environment
L_166_AllHuman
L_166_Oral
L_168_Hg19
L_168_Bacteria
L_168_Environment
L_169_Hg19
L_169_Bacteria
L_169_Environment
L_170_Hg19
L_170_Bacteria
L_170_Environment
L_171_Hg19
L_171_Bacteria
L_171_Environment
L_171_OtherHuman
L_172_Hg19
L_172_Bacteria
L_172_Environment
L_173_Hg19
L_173_Bacteria
L_173_Environment
S_067_Hg19
S_067_Bacteria
S_067_Environment
S_068_Hg19
S_068_Bacteria
S_068_Environment
S_069_Hg19
S_069_Bacteria
S_069_Environment
S_070_Hg19
S_070_Bacteria
S_070_Environment
S_071_Hg19
S_071_Bacteria
S_071_Environment
S_072_Hg19
S_072_Bacteria
S_072_Environment
S_073_Hg19
S_073_Bacteria
S_073_Environment
S_073_AllHuman
S_073_Pathogen
S_074_Hg19
S_074_Bacteria
S_074_Environment
S_075_Hg19
S_075_Bacteria
S_075_Environment
S_075_AllHuman
S_075_Pathogen
S_076_Hg19
S_076_Bacteria
S_076_Environment
S_077_Hg19
S_077_Bacteria
S_077_Environment
S_078_Hg19
S_078_Bacteria
S_078_Environment
S_079_Hg19
S_079_Bacteria
S_079_Environment
S_081_Hg19
S_081_Bacteria
S_081_Environment
S_082_Hg19
S_082_Bacteria
S_082_Environment
S_083_Hg19
S_083_Bacteria
S_083_Environment
S_084_Hg19
S_084_Bacteria
S_084_Environment
S_085_Hg19
S_085_Bacteria
S_085_Environment
S_087_Hg19
S_087_Bacteria
S_087_Environment
S_087_AllHuman
S_087_Pathogen

Supplementary Figure 9. The differences of DNA damage levels (δs) of human and bacterial/archaeal DNA in individual samples. Human DNA is in black, All bacterial/archaeal DNA is in grey and bacterial/archaeal DNA belonging to the environmental group is in green, all human-related in orange, oral in blue, other in yellow and pathogen in red.

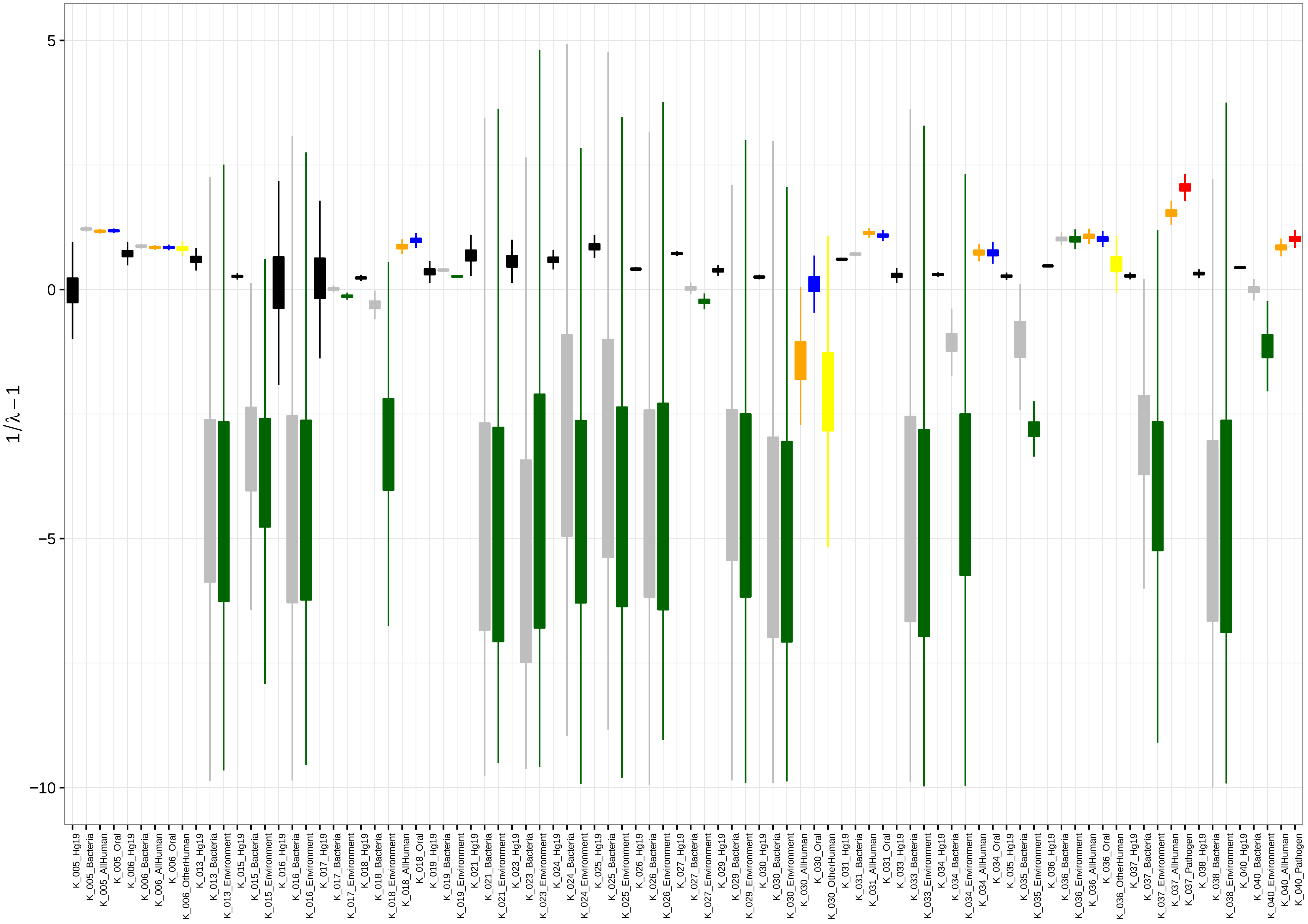

5
0
−5
−10
1/λ − 1
K_005_Hg19
K_005_Bacteria
K_005_AllHuman
K_005_Oral
K_006_Hg19
K_006_Bacteria
K_006_AllHuman
K_006_Oral
K_006_OtherHuman
K_013_Hg19
K_013_Bacteria
K_013_Environment
K_015_Hg19
K_015_Bacteria
K_015_Environment
K_016_Hg19
K_016_Bacteria
K_016_Environment
K_017_Hg19
K_017_Bacteria
K_017_Environment
K_018_Hg19
K_018_Bacteria
K_018_Environment
K_018_AllHuman
K_018_Oral
K_019_Hg19
K_019_Bacteria
K_019_Environment
K_021_Hg19
K_021_Bacteria
K_021_Environment
K_023_Hg19
K_023_Bacteria
K_023_Environment
K_024_Hg19
K_024_Bacteria
K_024_Environment
K_025_Hg19
K_025_Bacteria
K_025_Environment
K_026_Hg19
K_026_Bacteria
K_026_Environment
K_027_Hg19
K_027_Bacteria
K_027_Environment
K_029_Hg19
K_029_Bacteria
K_029_Environment
K_030_Hg19
K_030_Bacteria
K_030_Environment
K_030_AllHuman
K_030_Oral
K_030_OtherHuman
K_031_Hg19
K_031_Bacteria
K_031_AllHuman
K_031_Oral
K_033_Hg19
K_033_Bacteria
K_033_Environment
K_034_Hg19
K_034_Bacteria
K_034_Environment
K_034_AllHuman
K_034_Oral
K_035_Hg19
K_035_Bacteria
K_035_Environment
K_036_Hg19
K_036_Bacteria
K_036_Environment
K_036_AllHuman
K_036_Oral
K_036_OtherHuman
K_037_Hg19
K_037_Bacteria
K_037_Environment
K_037_AllHuman
K_037_Pathogen
K_038_Hg19
K_038_Bacteria
K_038_Environment
K_040_Hg19
K_040_Bacteria
K_040_Environment
K_040_AllHuman
K_040_Pathogen

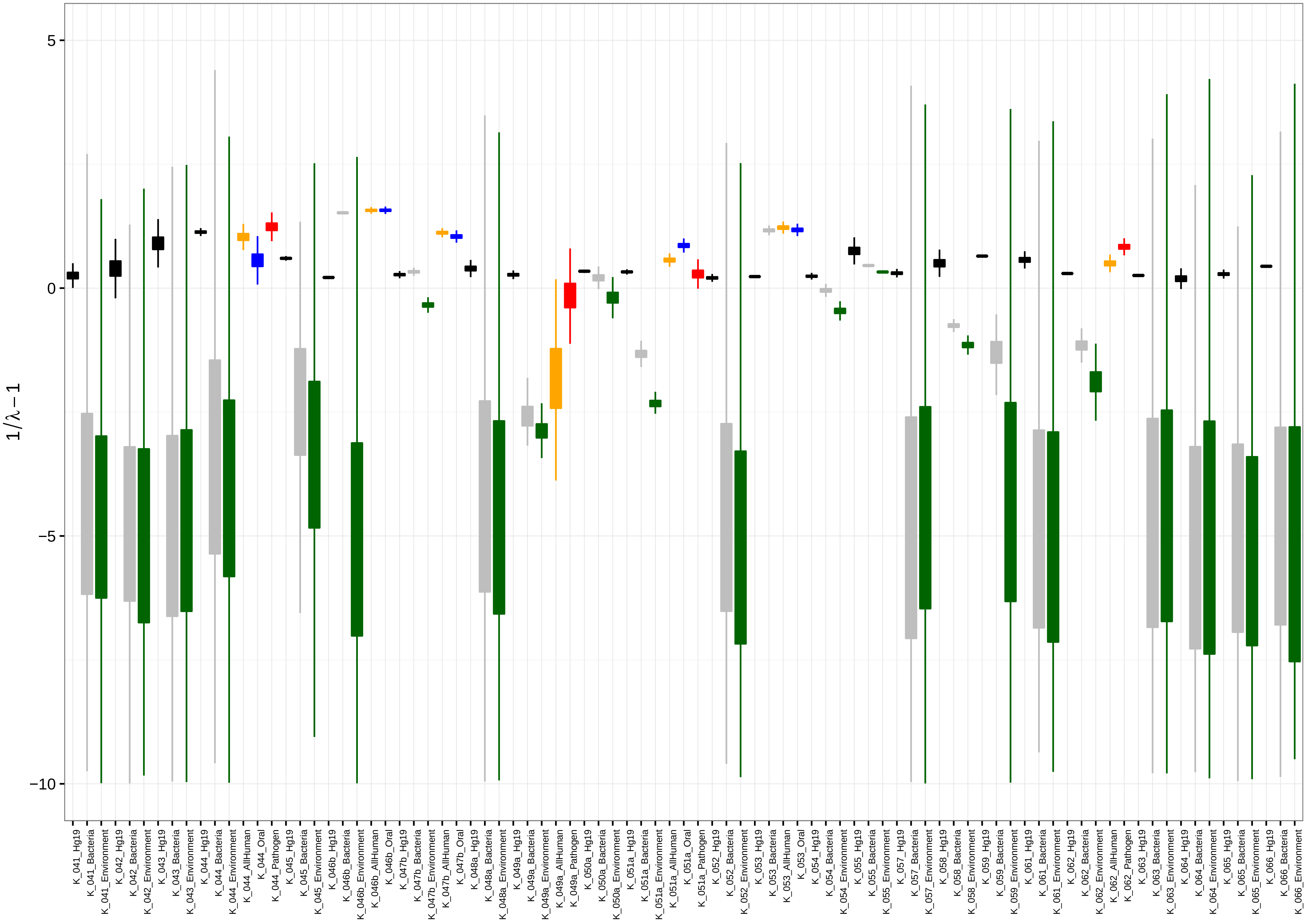
5
0
−5
−10
1/λ − 1
K_041_Hg19
K_041_Bacteria
K_041_Environment
K_042_Hg19
K_042_Bacteria
K_042_Environment
K_043_Hg19
K_043_Bacteria
K_043_Environment
K_044_Hg19
K_044_Bacteria
K_044_Environment
K_044_AllHuman
K_044_Oral
K_044_Pathogen
K_045_Hg19
K_045_Bacteria
K_045_Environment
K_046b_Hg19
K_046b_Bacteria
K_046b_Environment
K_046b_AllHuman
K_046b_Oral
K_047b_Hg19
K_047b_Bacteria
K_047b_Environment
K_047b_AllHuman
K_047b_Oral
K_048a_Hg19
K_048a_Bacteria
K_048a_Environment
K_049a_Hg19
K_049a_Bacteria
K_049a_Environment
K_049a_AllHuman
K_049a_Pathogen
K_050a_Hg19
K_050a_Bacteria
K_050a_Environment
K_051a_Hg19
K_051a_Bacteria
K_051a_Environment
K_051a_AllHuman
K_051a_Oral
K_051a_Pathogen
K_052_Hg19
K_052_Bacteria
K_052_Environment
K_053_Hg19
K_053_Bacteria
K_053_AllHuman
K_053_Oral
K_054_Hg19
K_054_Bacteria
K_054_Environment
K_055_Hg19
K_055_Bacteria
K_055_Environment
K_057_Hg19
K_057_Bacteria
K_057_Environment
K_058_Hg19
K_058_Bacteria
K_058_Environment
K_059_Hg19
K_059_Bacteria
K_059_Environment
K_061_Hg19
K_061_Bacteria
K_061_Environment
K_062_Hg19
K_062_Bacteria
K_062_Environment
K_062_AllHuman
K_062_Pathogen
K_063_Hg19
K_063_Bacteria
K_063_Environment
K_064_Hg19
K_064_Bacteria
K_064_Environment
K_065_Hg19
K_065_Bacteria
K_065_Environment
K_066_Hg19
K_066_Bacteria
K_066_Environment

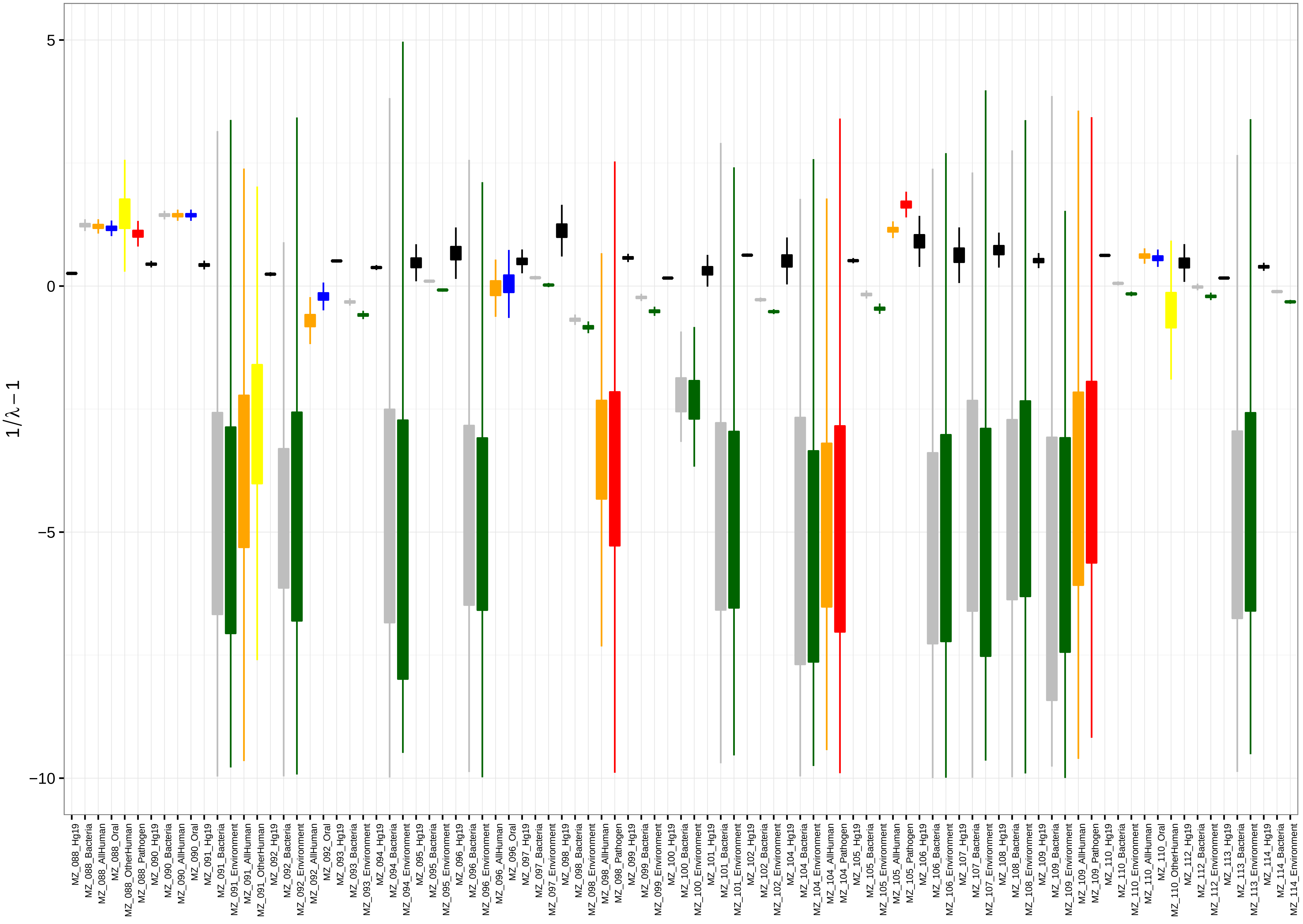

1/λ − 1
5
0
−5
−10
MZ_088_Hg19
MZ_088_Bacteria
MZ_088_AllHuman
MZ_088_Oral
MZ_088_OtherHuman
MZ_088_Pathogen
MZ_090_Hg19
MZ_090_Bacteria
MZ_090_AllHuman
MZ_090_Oral
MZ_091_Hg19
MZ_091_Bacteria
MZ_091_Environment
MZ_091_AllHuman
MZ_091_OtherHuman
MZ_092_Hg19
MZ_092_Bacteria
MZ_092_Environment
MZ_092_AllHuman
MZ_092_Oral
MZ_093_Hg19
MZ_093_Bacteria
MZ_093_Environment
MZ_094_Hg19
MZ_094_Bacteria
MZ_094_Environment
MZ_095_Hg19
MZ_095_Bacteria
MZ_095_Environment
MZ_096_Hg19
MZ_096_Bacteria
MZ_096_Environment
MZ_096_AllHuman
MZ_096_Oral
MZ_097_Hg19
MZ_097_Bacteria
MZ_097_Environment
MZ_098_Hg19
MZ_098_Bacteria
MZ_098_Environment
MZ_098_AllHuman
MZ_098_Pathogen
MZ_099_Hg19
MZ_099_Bacteria
MZ_099_Environment
MZ_100_Hg19
MZ_100_Bacteria
MZ_100_Environment
MZ_101_Hg19
MZ_101_Bacteria
MZ_101_Environment
MZ_102_Hg19
MZ_102_Bacteria
MZ_102_Environment
MZ_104_Hg19
MZ_104_Bacteria
MZ_104_Environment
MZ_104_AllHuman
MZ_104_Pathogen
MZ_105_Hg19
MZ_105_Bacteria
MZ_105_Environment
MZ_105_AllHuman
MZ_105_Pathogen
MZ_106_Hg19
MZ_106_Bacteria
MZ_106_Environment
MZ_107_Hg19
MZ_107_Bacteria
MZ_107_Environment
MZ_108_Hg19
MZ_108_Bacteria
MZ_108_Environment
MZ_109_Hg19
MZ_109_Bacteria
MZ_109_Environment
MZ_109_AllHuman
MZ_109_Pathogen
MZ_110_Hg19
MZ_110_Bacteria
MZ_110_Environment
MZ_110_AllHuman
MZ_110_Oral
MZ_110_OtherHuman
MZ_112_Hg19
MZ_112_Bacteria
MZ_112_Environment
MZ_113_Hg19
MZ_113_Bacteria
MZ_113_Environment
MZ_114_Hg19
MZ_114_Bacteria
MZ_114_Environment

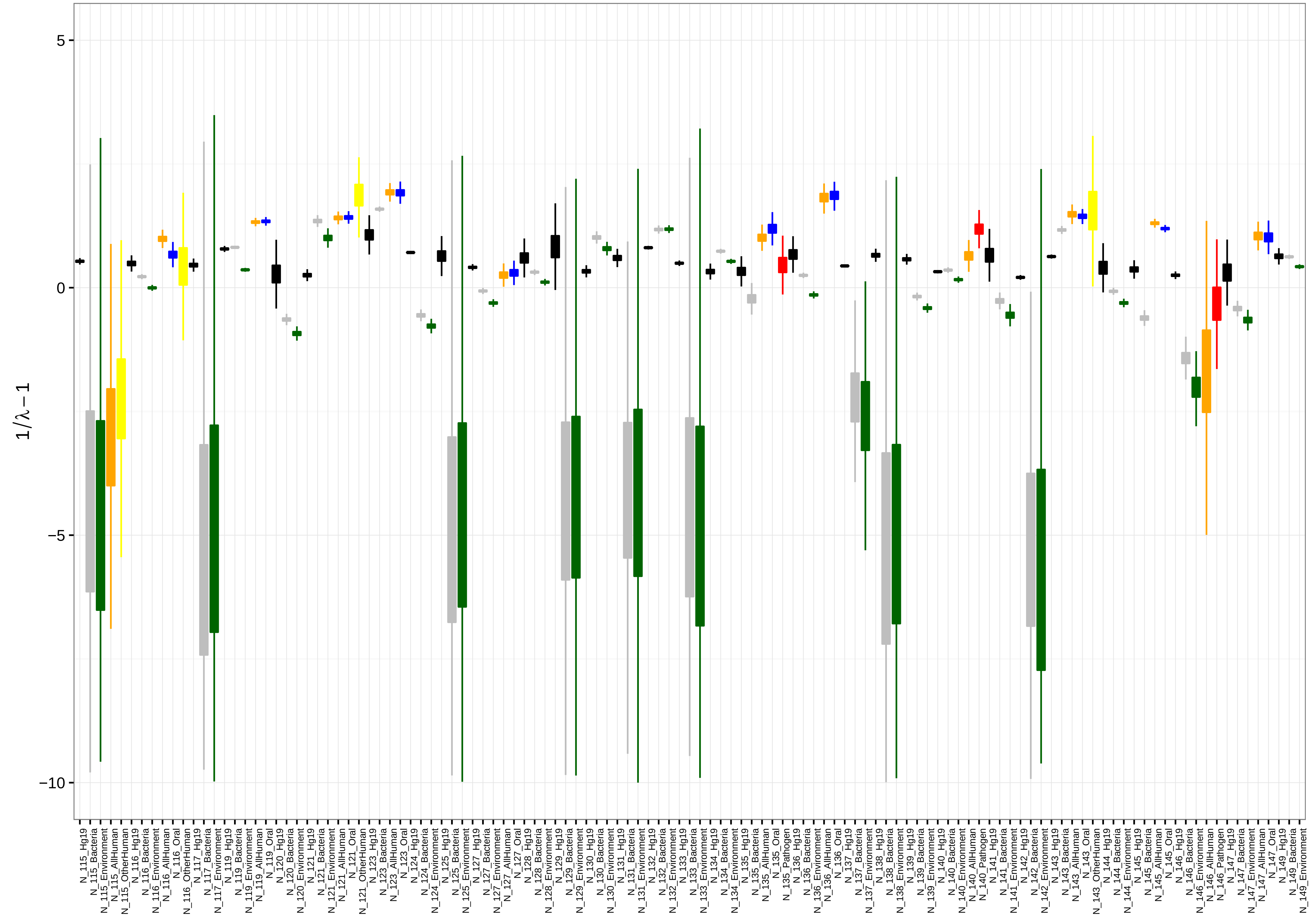

1/λ − 1
5
0
−5
−10
N_115_Hg19
N_115_Bacteria
N_115_Environment
N_115_AllHuman
N_115_OtherHuman
N_116_Hg19
N_116_Bacteria
N_116_Environment
N_116_AllHuman
N_116_Oral
N_116_OtherHuman
N_117_Hg19
N_117_Bacteria
N_117_Environment
N_119_Hg19
N_119_Bacteria
N_119_Environment
N_119_AllHuman
N_119_Oral
N_120_Hg19
N_120_Bacteria
N_120_Environment
N_121_Hg19
N_121_Bacteria
N_121_Environment
N_121_AllHuman
N_121_Oral
N_121_OtherHuman
N_123_Hg19
N_123_Bacteria
N_123_AllHuman
N_123_Oral
N_124_Hg19
N_124_Bacteria
N_124_Environment
N_125_Hg19
N_125_Bacteria
N_125_Environment
N_127_Hg19
N_127_Bacteria
N_127_Environment
N_127_AllHuman
N_127_Oral
N_128_Hg19
N_128_Bacteria
N_128_Environment
N_129_Hg19
N_129_Bacteria
N_129_Environment
N_130_Hg19
N_130_Bacteria
N_130_Environment
N_131_Hg19
N_131_Bacteria
N_131_Environment
N_132_Hg19
N_132_Bacteria
N_132_Environment
N_133_Hg19
N_133_Bacteria
N_133_Environment
N_134_Hg19
N_134_Bacteria
N_134_Environment
N_135_Hg19
N_135_Bacteria
N_135_AllHuman
N_135_Oral
N_135_Pathogen
N_136_Hg19
N_136_Bacteria
N_136_Environment
N_136_AllHuman
N_136_Oral
N_137_Hg19
N_137_Bacteria
N_137_Environment
N_138_Hg19
N_138_Bacteria
N_138_Environment
N_139_Hg19
N_139_Bacteria
N_139_Environment
N_140_Hg19
N_140_Bacteria
N_140_Environment
N_140_AllHuman
N_140_Pathogen
N_141_Hg19
N_141_Bacteria
N_141_Environment
N_142_Hg19
N_142_Bacteria
N_142_Environment
N_143_Hg19
N_143_Bacteria
N_143_AllHuman
N_143_Oral
N_143_OtherHuman
N_144_Hg19
N_144_Bacteria
N_144_Environment
N_145_Hg19
N_145_Bacteria
N_145_AllHuman
N_145_Oral
N_146_Hg19
N_146_Bacteria
N_146_Environment
N_146_AllHuman
N_146_Pathogen
N_147_Hg19
N_147_Bacteria
N_147_Environment
N_147_AllHuman
N_147_Oral
N_149_Hg19
N_149_Bacteria
N_149_Environment

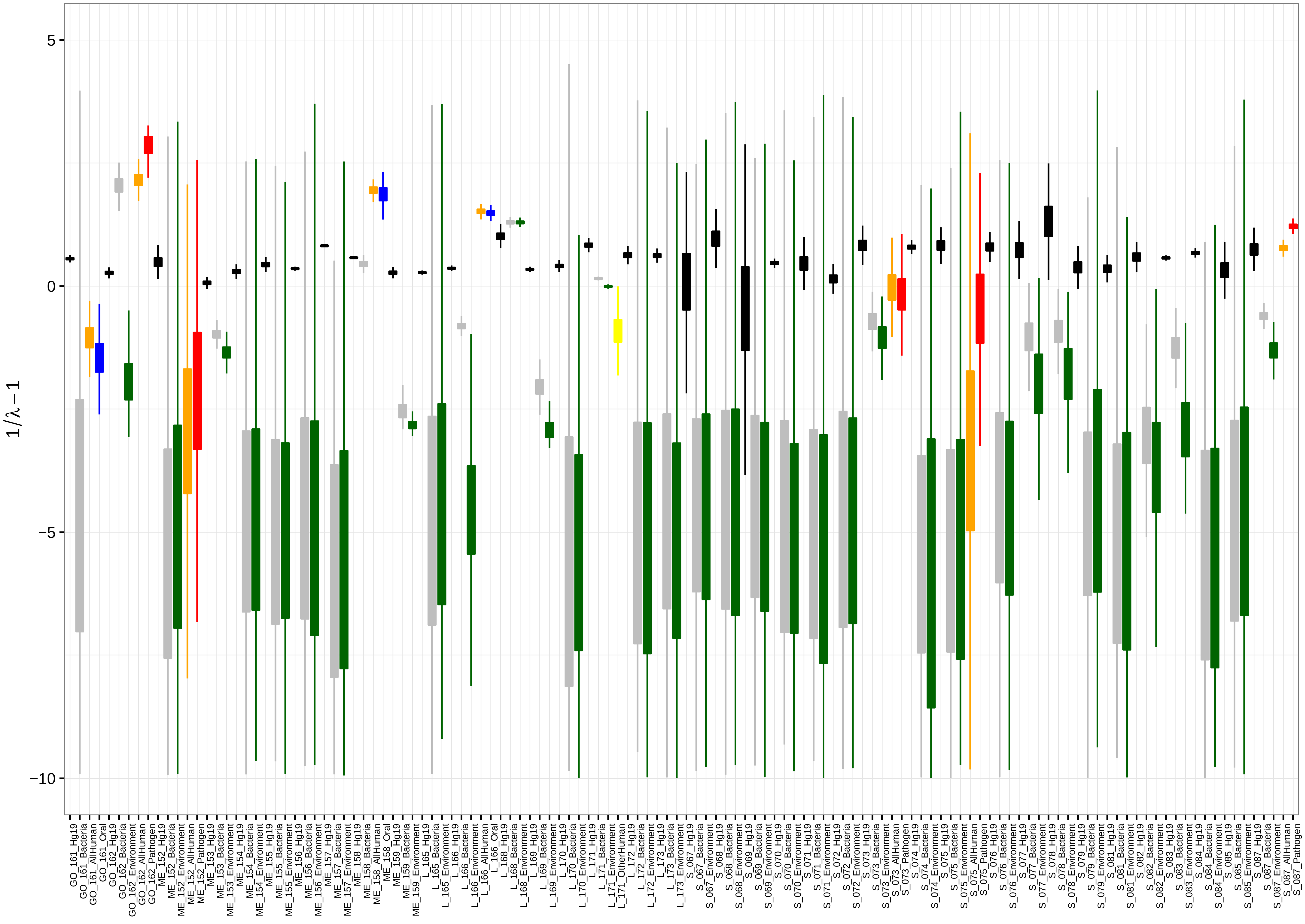
5
0
−5
−10
1/λ − 1
GO_161_Hg19
GO_161_Bacteria
GO_161_AllHuman
GO_161_Oral
GO_162_Hg19
GO_162_Bacteria
GO_162_Environment
GO_162_AllHuman
GO_162_Pathogen
ME_152_Hg19
ME_152_Bacteria
ME_152_Environment
ME_152_AllHuman
ME_152_Pathogen
ME_153_Hg19
ME_153_Bacteria
ME_153_Environment
ME_154_Hg19
ME_154_Bacteria
ME_154_Environment
ME_155_Hg19
ME_155_Bacteria
ME_155_Environment
ME_156_Hg19
ME_156_Bacteria
ME_156_Environment
ME_157_Hg19
ME_157_Bacteria
ME_157_Environment
ME_158_Hg19
ME_158_Bacteria
ME_158_AllHuman
ME_158_Oral
ME_159_Hg19
ME_159_Bacteria
ME_159_Environment
L_165_Hg19
L_165_Bacteria
L_165_Environment
L_166_Hg19
L_166_Bacteria
L_166_Environment
L_166_AllHuman
L_166_Oral
L_168_Hg19
L_168_Bacteria
L_168_Environment
L_169_Hg19
L_169_Bacteria
L_169_Environment
L_170_Hg19
L_170_Bacteria
L_170_Environment
L_171_Hg19
L_171_Bacteria
L_171_Environment
L_171_OtherHuman
L_172_Hg19
L_172_Bacteria
L_172_Environment
L_173_Hg19
L_173_Bacteria
L_173_Environment
S_067_Hg19
S_067_Bacteria
S_067_Environment
S_068_Hg19
S_068_Bacteria
S_068_Environment
S_069_Hg19
S_069_Bacteria
S_069_Environment
S_070_Hg19
S_070_Bacteria
S_070_Environment
S_071_Hg19
S_071_Bacteria
S_071_Environment
S_072_Hg19
S_072_Bacteria
S_072_Environment
S_073_Hg19
S_073_Bacteria
S_073_Environment
S_073_AllHuman
S_073_Pathogen
S_074_Hg19
S_074_Bacteria
S_074_Environment
S_075_Hg19
S_075_Bacteria
S_075_Environment
S_075_AllHuman
S_075_Pathogen
S_076_Hg19
S_076_Bacteria
S_076_Environment
S_077_Hg19
S_077_Bacteria
S_077_Environment
S_078_Hg19
S_078_Bacteria
S_078_Environment
S_079_Hg19
S_079_Bacteria
S_079_Environment
S_081_Hg19
S_081_Bacteria
S_081_Environment
S_082_Hg19
S_082_Bacteria
S_082_Environment
S_083_Hg19
S_083_Bacteria
S_083_Environment
S_084_Hg19
S_084_Bacteria
S_084_Environment
S_085_Hg19
S_085_Bacteria
S_085_Environment
S_087_Hg19
S_087_Bacteria
S_087_Environment
S_087_AllHuman
S_087_Pathogen

Supplementary Figure 10. The differences of DNA damage levels (λ , expressed as: 1/λ-1) of human and bacterial/archaeal DNA in individual samples. Human DNA is in black, All bacterial/archaeal DNA is in grey and bacterial/archaeal DNA belonging to the environmental group is in green, all human-related in orange, oral in blue, other in yellow and pathogen in red.
