# Supplementary material for: Comprehensive analysis of microorganisms accompanying human archaeological remains
Source: Gigascience. 2017 Jun 13;6(7):1–13. doi: 10.1093/gigascience/gix044 (PMC5965364; doi:10.1093/gigascience/gix044)
Supplement: aDNA_microorganisms_Figlerowicz_Supplementary_Tables_leg.docx [file gix044_adna_microorganisms_figlerowicz_supplementary_tables_leg.docx]

Supplementary Table 1. Summarized information on NGS datasets used within this study. The first column form the left lists samples IDs. Column 2 comprises information on C14 dating of selected samples.

Column 3 and column 4 describe the depth of sequencing (number of raw and filtered reads). Columns 5 and 6 describe the reads that map to the human genome (number, percentage). Columns 7 and 8 describe reads mapping to the Metaphlan2 markers DB (number, percentage). Column 9 describes the number of reads that mapped to the prokaryotic markers only. Columns 10-16 describe the percentage (within a sample) of viruses/viroids, eukaryote, all prokaryote, environmental prokaryote, oral prokaryote, other human-related prokaryote and potential pathogens, respectively. Columns 17-22 describe the number of identified bacterial/archaeal taxa and Shannon index on class, family and species level respectively.

Supplementary Table 2. Summarized information on bacterial/archaeal taxa (Column 1) identified within samples (Columns 5-165). Column 2-4 describe taxon gram stain type, respiratory type and its typical habitat, respectively.

Supplementary Table 3. The information on 11 samples used for the validation of results obtained in shallow sequencing experiment. The first column form the left lists samples IDs. Column 2 describes the total number of filtered reads. Column 3 and 4 describe the reads that mapped to the Metaphlan2 markers DB (number, percentage). Column 5 describes the percentage of prokaryote identified in a sample. Column 6 and 7 describe the number of bacterial/archaeal classes and Shannon index.

Supplementary Table 4. Summarized information on 77 bacterial and archaeal species (Column 1) selected for aDNA damage analysis. Columns 2-4 describe species gram stain type, respiratory type and its habitat, respectively. Columns 5-12 describe the number of samples in which the species were present in more % than the threshold (80%, 70%, 60%, 50%, 40%, 30%, 20%, 10%, 1% respectively). Column 13 describes the maximal percentage of a species observed. Column 14 describes the overall percentage of a species in all samples. Columns 15-154 describe the species percentage in an individual samples. A) Table summarizes the number of samples with species present in more than the threshold and their percentage in respect to the all of identified species.
