# Supplementary material for: Comprehensive analysis of microorganisms accompanying human archaeological remains
Source: Gigascience. 2017 Jun 13;6(7):1–13. doi: 10.1093/gigascience/gix044 (PMC5965364; doi:10.1093/gigascience/gix044)
Supplement: Response_to_Reviewer_Comments_Original_Submission.pdf [file gix044_response_to_reviewer_comments_original_submission.pdf]

Author's Response To Reviewer Comments

Dear Editor,
Once again, thank you for giving us the opportunity to revise our manuscript. We found all of your and the Reviewers' comments very useful in guiding us through the revision. Consequently, we considered all the points that were raised and modified our manuscript accordingly.

All introduced changes are marked in the revised manuscript in red. A detailed discussion of the Reviewers' comments is appended below in the point-by-point response. I hope the revised version of our manuscript will meet the high standards of the GigaScience journal.

Yours sincerely,
Marek Figlerowicz

Editor

Your manuscript "Comprehensive analysis of microorganisms accompanying human archaeological remains" (GIGA-D-17-00056) has been assessed by our reviewers. Based on these reports, and my own assessment as Editor, I am pleased to inform you that it is potentially acceptable for publication in GigaScience, once you have carried out some essential revisions suggested by our reviewers.
Their reports, together with any other comments, are below. Please also take a moment to check our website at http://giga.edmgr.com/ for any additional comments that were saved as attachments.
In addition to the reviewer's minor suggested changes below, please ensure you make the following edits to the paper:

1) Please add a Potential Implications section before "Methods" (see: https://academic.oup.com/gigascience//pages/research)

We added Potential Implications section, see page: 14, lines:397-405.

2) Under "Availability of Supporting data" please make the SRA URL into a Reference (#22) and add the URL to the References list.
3) Under "Availability of Supporting data" please also add the following sentence "Other data further supporting this work can be found in the GigaScience repository, GigaDB {Ref#23}." Please add a place holder Ref #23 - to be updated with the GigaDB DOI (to be issued).
As requested, we added the two references, but at the end of the references list (INSTRUCTIONS TO AUTHORS - "All references, including URLs, must be numbered consecutively, in square brackets, in the order in which they are cited in the text").

Once you have made the necessary corrections, please submit a revised manuscript online at: http://giga.edmgr.com/

If you have forgotten your username or password please use the "Send Login Details" link to get

your login information. For security reasons, your password will be reset.

Please include a point-by-point within the 'Response to Reviewers' box in the submission system. Please ensure you describe additional experiments that were carried out and include a detailed rebuttal of any criticisms or requested revisions that you disagreed with. Please also ensure that your revised manuscript conforms to the journal style, which can be found in the Instructions for Authors on the journal homepage.
The due date for submitting the revised version of your article is 10 Jul 2017.
Reviewer 1

Philips and colleagues satisfactorily addressed most of my comments, and I feel the paper has improved a lot.
But I am afraid I still have a few suggestions and one main concern:

1) Regarding my concern number 3 in the previous paper assessment (post-mortem damage in viruses). I understand the amount of virus markers covered by their sequencing data is low, but this should not be a limitation to know if virus DNA is ancient. Once they have identified the virus genera (and they claim to have done so), the authors can always download a bunch virus genomes of this genera, and remap all reads against them. If they manage to identify the correct species (or a closely-related one), the authors can perhaps retain an enough number of reads that is acceptable to assess post-mortem damage. They can just use mapDamage supplying the -no-stats flags, to obtain the raw plots without Bayesian parameter estimation. Simpler approaches can be also informative, like transitions/transversions ratios along the reads, or read length. I am VERY surprised by the large amount of virus DNA found (and also the amount of archea for some samples). My recollection is that we have never found something similar. In my opinion, further non-speculative understanding of this point would be very convenient.

As we have mentioned in our manuscript the most abundant viruses in the analyzed samples were RNA viruses of plants, e.g. Dasheen mosaic virus (58% of all identified viruses/viroids) and Vicia cryptic virus (26.7%). It would be extremely interesting to determine the pattern of their genomes damage, unfortunately at present it is rather impossible because their genomes are composed of RNA. All methods that are used for the damage analysis have been designed for organisms which genomes are composed of DNA. These methods do not take into account several important features that are characteristic for the replication of RNA virus genomes. RNA-based viruses encode their own RNA-dependent RNA polymerases that lack proofreading activity. As established in in vitro experiments, these enzyme exhibits an error rate that ranges from 10−4 to 10−6 per site. In addition to being error prone RNA replication is extremely efficient and in some cases it enables the production of 1012 virions per day in a single individual. As a result of highly effective but low-fidelity replication, the mutation rate of RNA virus genomes is several order of magnitude higher than the mutation rate of DNA genomes. Consequently, RNA virus does not form a homogenous population but circulates in a host organism as a pool of phylogenetically related distinct variants. To describe such a complex structure of virus population the concept of quasispecies is used.

Minor issues:
1) In pag 3 line 80, "rich" should be "reach".

Corrected.

2) I agree it is rare the case that microbial profiling is the main topic of an aDNA paper, but it does not mean ancient human remains have been "poorly" profiled. Perhaps rephrase to say that a comparative survey is lacking.

The Reviewer is right. We rephrased this sentence (see page: 4, line: 83).

3) although conclusions look sound, the statistics supporting their conclusions might not be the most appropriate. For example, no error bars in Fig.7.

As suggested, we added error bars in Figure 7.

What is correlation R? Do they mean Pearson correlation coefficient (r^2)? I cannot find it in methods. ANOVA assumes same variances for the groups being compared. I do not think it is the case in Fig. 1B and Fig 1C, for example. Actually, it would be interesting to know whether the older the site, the more variance in the endogenous content.

The Reviewer is right. R is Pearson correlation – an appropriate definition has been introduced (Methods, see page: 16, line: 455). We also replaced ANOVA test with non-parametric Kruskal-Wallis (also called One-way ANOVA on ranks) test and accordingly, we modified the main text as the Reviewer suggested (see page: 5, lines:118-120; page 5, lines: 125-130).
We also added lacking methods in the Methods paragraph (see page:16, lines: 450-451; page: 16, lines: 453-454).

4) Metaphlan2 database could provide limited power to separate samples according to a categorical variable (age, conservation conditions, etc), because they are based on a limited number of markers from a limited number of taxa. I would strongly recommend trying to cluster samples based on k-mer distances between exogenous reads. This would provide an unbiased and complementary approach to corroborate absence of clustering, as the sample-specificity is one of their major claims. See for example https://bmcbioinformatics.biomedcentral.com/articles/10.1186/s12859-015-0875-7. Hope it helps!

We thank the Reviewer for this idea of samples clustering. As suggested, we run k-mer clustering, to check if approach in which we take into account all exogenous DNA reads will separate samples. Unfortunately we did not obtained clusters corresponding to the samples age, storage or archaeological site, what further supports our previews results. (see new Supplementary Figure 6, page: 9, lines: 237-239). Lastly, we would like to point out that suggested approach allows for analyses of not only microbial DNA reads, but also plants, animals, fungal reads. We suppose that this approach might introduce additional bias to the analyses and even might lead to the misleading results.

Reviewer 2

1) In their manuscript the authors describe a metagenomic assessment of archaeological remains with a focus on bacterial communities. The study targets multiple archaeological samples of varying age and storage conditions. The authors attempt to differentiate between bacterial species that might have been present before the individuals death and environmental contamination. The methods employed by the authors are suitable for metagenomic analyses and the applied statistics are appropriate. However, considering the fact that DNA of human endogenous pathogens is usually only found in very low amounts in archaeological samples, the concept of MetaPhlAn might lack sensitivity. Only reads mapping to predefined marker regions are used and therefore most of the data is discarded. This limits the possibilities for the authentication of species identifications.

We agree with the Reviewer that the identification of pathogens in shallow libraries (using MetaPhlAn) requires further confirmations (e.g. mapping to the whole microbial genome, experimental validation) and might be a good start for further study of certain pathogenic species, what was not the aim of this report see Discussion page: 14, lines: 393-394 and Potential Implications page: 14, lines: 402-404.
Our goal was to characterize the microbiome of archaeological samples and we do believe that identified microbes represent fairly well environmental or human-associated, pathogenic species. This fact is reflected in statistically significant differences in DNA damage observed in those groups (DNA damage analysis were done with reads mapping to the whole microbial genomes, not only marker sequences).

2) The authors discuss these limitations also in the context of the fact that many human related bacteria have close relatives in the soil. I recommend to discuss this a bit more specifically for individual taxa. Some suggestions are given below.

As suggested, we added adequate comments in the Discussion (see page 14, line:381; page: 14, lines:382-384). See also answer to the Point 4.

3) Overall, this study provides a useful comparative resource for the assessment of bacterial communities that accompany human remains in an archaeological context. In the abstract the authors claim that "comprehensive characterization of microbial DNA accompanying human remains has never been done." In fact many studies do comprehensively characterize microbial DNA in the context of human remains in addition to focusing on a specific pathogenic species. I assume what the authors mean by 'comprehensive' is that this has not been done systematically for a wide range of different samples. The authors should make this clearer also in the introduction.

Corrected as the Reviewer suggested (see page: 2, lines:27-28).

4) The authors indicate that they found substantial amounts of Methanobrevibacter and describe it as "typically found in the human digestive system". It is very probable that the species M. oralis was detected here, which is commonly found in the oral cavity. Furthermore, the authors indicate that Bordetella was found as a taxon potentially representing a human pathogen. The authors mention in the discussion that B. bronchiseptica can also survive in the environment. In addition, it should be mentioned that this genus also contains species that are commonly found in

the environment, such as B. petrii. The same is true for other taxa that the authors call 'potential pathogens' such as Stenotrophomonas and Clostridium. In particular for Clostridia it is rather unlikely that one of the individuals was infected with a bacterium of this genus. The reason that it is detected in the context of human remains is not surprising as it colonizes the human body after death. In general the authors only distinguish between human-related and environmental taxa. Bacteria involved in body decomposition overlap with both groups in some way. Therefore, I think that these bacteria should be mentioned as a third component in addition to human endogenous bacteria and environmental taxa.

We thank the Reviewer for this comment. We agree with the Reviewer that distinguishing between environmental and pathogenic species belonging to the same genus (like Bordetella) might be problematic, despites the usage of unique markers. This is why we believe that DNA damage patterns analysis might be helpful in preliminary study of ancient pathogens. See also answer to the Point 1.
We also realize that both environmental and human-associated species take part in body decomposition. However, despites a high number of analyzed samples deeper segregation would decrease statistical power of analyses and would made results non-conclusive. Similar situation was observed for division of human-related group into three subgroups (oral, pathogen, other) which caused the results to be of low statistical power and generally inconclusive.
According to the Reviewers suggestion we added appropriate comments in the Discussion (see page 12, line:319; page 14, line:381; page: 14, lines:382-384).

In the discussion the authors state "Our analyses revealed that some of them showed the DNA damage patterns closer to the environmental microbes than to the human associated ones." This statement does not make sense to me. They might be more similar to environmental taxa on the sequence level, but I do not see what that has to do with damage patterns.

We rephrased this sentence to be clearer (see page: 14, lines: 385-387). In this sentence we do not discuss the similarity between DNA sequences but similarity between DNA damage patterns. If we analyze human remains the DNA damage pattern observed for bacterial DNA is similar to the damage pattern observed for human aDNA one can assume that both are of similar age.

Furthermore, the authors state "This observation may indicate that DNA of some potential pathogens can be acquired from the environment after the organisms death." I do not understand the intention of this statement. Environmental contamination is obvious and one of the subjects of the study. Why should environmental taxa that are similar to human pathogens be excluded from this? I suggest to omit this statement.

We removed this sentence, as suggested.

Also in the discussion it is stated: "We showed that identification of bacteria/archaea species accompanying the organism before death is possible using standard aDNA extraction protocols and shallow shotgun sequencing." This is a very general statement. Other studies have shown that before to various extent. Maybe the authors can be more specific here. In addition, any bacterial taxon identified in this study should rather be seen as a candidate as the amount of data is not sufficient to authenticate the species identifications reliably.

The Reviewer is right. We changed this sentence according to the Reviewers comment (see page: 14, lines: 389).
