# Supplementary material for: Comprehensive analysis of microorganisms accompanying human archaeological remains
Source: Gigascience. 2017 Jun 13;6(7):1–13. doi: 10.1093/gigascience/gix044 (PMC5965364; doi:10.1093/gigascience/gix044)
Supplement: Response_to_Reviewer_Comments_Revision_1.pdf [file gix044_response_to_reviewer_comments_revision_1.pdf]

Author's Response To Reviewer Comments

Dear Dr. Nogoy,
We found all of your and the Reviewers' comments very useful in guiding us through the revision. Consequently, we considered all the points that were raised and modified our manuscript accordingly. As you requested, we added RRIDs for certain tools and introduced minor editorial corrections in the main text.

A detailed discussion of the Reviewers’ comments is appended below in the point-by-point response. I hope the revised version of our manuscript will meet the high standards of the GigaScience journal.

Yours sincerely,
Marek Figlerowicz

Reviewer #1: I highly appreciate authors considered all my comments, and think this paper is should be now suitable for publication. I have a few minor comments that the authors can address without further revisions, under the supervision/decision of the editor.

- line 84, I would change: "until now were not comparatively surveyed" by "have not been exhaustively compared so far"

Corrected.

- line 88, parenthesis not needed in ([41-44]). Same in line 352

Corrected.

- Number of decimals is inconsistent. Sometimes 4, and sometimes 5 floating digits.

Corrected, page: 5 lines: 120, 129; page: 11, lines: 292, 315.

- line 128, I would replace "the minor statistically significant difference" by "Marginal statistical significance was"

Rephrased, as suggested.

- the authors claim "we showed that the composition of microbiome of archeological remains does not depend on the way and time of storage". I do not think this conclusion is necessarily true, given their tests and many other publications contradicting it. What authors found is that the variance amongst samples is so high that it obscures any trend created by the way and time of storage. In other words,

that sample peculiarities are more important for microbial composition than way and time of storage. It does not mean that way or time of storage are not important. Please, check and rephrase across the whole manuscript.

The Reviewer is right, we oversimplify claiming that “the composition of microbiome of archaeological remains does not depend on the way and time of storage". Our intention was to point out the individual character of each sample microbiome. We clarified it through the text, please see: Page 12, lines:340-344; Page 15, lines: 417-418.

And finally what I find more controversial is still the massive virus content. I am not going to ask for the same once again, but in the previous round of reviews, I asked for some kind of damage profiling of virus reads, because I am a bit puzzled about the proportion of viruses in some samples. Although viruses are discarded in many aDNA metagenomic analyses, the fact that some samples show >60% is really shocking. Does it mean that most of the sequencing effort is going for virus sequencing? Given the importance that this could have in aDNA, I do not think the authors can just briefly discuss it, and disregard the virus fraction for the rest of the paper. I felt and I still feel that a more accurate analysis/interpretation is compulsory.
The authors replied they cannot address my suggestion because "unfortunately at present it is rather impossible because their genomes are composed of RNA". I am not a lab expert, but ... how can you sequence RNA viruses using DNA sequencing protocols? Are they retroviruses? Are they integrated in the host genomes? Based on table S1, it seems to be a correlation between virus and endogenous content, but it could be indirectly explained by the fraction of prokaryotes (i.e. more bacteria, less endogenous and less viruses). I have discussed with two colleagues, experts in aDNA metagenomics, and they are equally confused. They even suggested contamination, as apparently you did not sequence the blanks, and the 100-fold DNA enrichment between extracts and blanks is not much for aDNA standards. But we all agree that 1% of contamination cannot drive the >60% of virus found in some aDNA remains, unless there are sequencing artifacts as recently shown for Illumina HiSeq 4000. I am not saying the paper should be rejected. On the contrary, as it is mainly descriptive paper, I think it is more than ok to describe potential caveats in aDNA metagenomics. But I would strongly recommend to strength their discussion/interpretation for this particular point, as it looks crucial, at least to avoid suspicious interpretations (as my colleagues suggested).

According to the Reviewer suggestion, we added an additional paragraph in the Discussion (see page: 13, lines: 367-377) to deeper discuss identification of RNA viruses issue.

Reviewer #2: Philips and colleagues provide a revised version of their manuscript "Comprehensive analysis of microorganisms accompanying human archaeological remains" where most of the comments have been addressed.

In line 209 Methanobrevibacter, which was found in abundance in the samples, is still described as "typically found in the human digestive system". I still think that M. oralis needs to be pointed out as the

candidate here, as it is commonly found in the oral cavity. In the context of this work the detection of M. oralis makes also much more sense than detecting gut bacteria.

As suggested, we added an appropriate comment, please see page: 8, lines: 209-213.

Minor points:

Line 382: "Bordetella genus also contains species that is commonly found in the environment, Bordetella petrii."
For clarity I suggest to rephrase as:
"The genus Bordetella also contains species that are commonly found in the environment, such as Bordetella petrii."

Rephrased as suggested, page: 14, lines: 400-401.
