# Supplementary material for: Comprehensive analysis of microorganisms accompanying human archaeological remains
Source: Gigascience. 2017 Jun 13;6(7):1–13. doi: 10.1093/gigascience/gix044 (PMC5965364; doi:10.1093/gigascience/gix044)
Supplement: Reviewer_1_Report_(Original_Submission).pdf [file gix044_reviewer_1_report_(original_submission).pdf]

Reviewer Report

Title: Comprehensive analysis of microorganisms accompanying human archaeological remains

Version: Original Submission **Date:** 3/31/2017

Reviewer name: Pablo Librado

Reviewer Comments to Author:

Philips and colleagues satisfactorily addressed most of my comments, and I feel the paper has improved a lot. But I am afraid I still have a few suggestions and one main concern:

Regarding my concern number 3 in the previous paper assessment (post-mortem damage in viruses). I understand the amount of virus markers covered by their sequencing data is low, but this should not be a limitation to know if virus DNA is ancient. Once they have identified the virus genera (and they claim to have done so), the authors can always download a bunch virus genomes of this genera, and remap all reads against them. If they manage to identify the correct species (or a closely-related one), the authors can perhaps retain an enough number of reads that is acceptable to assess post-mortem damage. They can just use mapDamage supplying the -no-stats flags, to obtain the raw plots without Bayesian parameter estimation. Simpler approaches can be also informative, like transitions/transversions ratios along the reads, or read length. I am VERY surprised by the large amount of virus DNA found (and also the amount of archea for some samples). My recollection is that we have never found something similar. In my opinion, further non-speculative understanding of this point would be very convenient.

Minor issues:

- In pag 3 line 80, "rich" should be "reach".

- I agree it is rare the case that microbial profiling is the main topic of an aDNA paper, but it does not mean ancient human remains have been "poorly" profiled. Perhaps rephrase to say that a comparative survey is lacking.

- although conclusions look sound, the statistics supporting their conclusions might not be the most appropriate. For example, no error bars in Fig.7. What is correlation R? Do they mean Pearson correlation coefficient (r^2)? I cannot find it in methods. ANOVA assumes same variances for the groups being compared. I do not think it is the case in Fig. 1B and Fig 1C, for example. Actually, it would be interesting to know whether the older the site, the more variance in the endogenous content.

- Metaphlan2 database could provide limited power to separate samples according to a categorical variable (age, conservation conditions, etc), because they are based on a limited number of markers from a limited number of taxa. I would strongly recommend trying to cluster samples based on k-mer distances between exogenous reads. This would provide an unbiased and complementary approach to

corroborate absence of clustering, as the sample-specificity is one of their major claims. See for example https://bmcbioinformatics.biomedcentral.com/articles/10.1186/s12859-015-0875-7.

Hope it helps!

Methods

Are the methods appropriate to the aims of the study, are they well described, and are necessary controls included? Yes

Conclusions

Are the conclusions adequately supported by the data shown? Yes

Reporting Standards

Does the manuscript adhere to the journal's guidelines on minimum standards of reporting? Yes Choose an item.

Statistics

Are you able to assess all statistics in the manuscript, including the appropriateness of statistical tests used? Yes, and I have assessed the statistics in my report.

Quality of Written English

Please indicate the quality of language in the manuscript: Needs some language corrections before being published

Declaration of Competing Interests

Please complete a declaration of competing interests, considering the following questions:

- Have you in the past five years received reimbursements, fees, funding, or salary from an organisation that may in any way gain or lose financially from the publication of this manuscript, either now or in the future?
- Do you hold any stocks or shares in an organisation that may in any way gain or lose financially from the publication of this manuscript, either now or in the future?
- Do you hold or are you currently applying for any patents relating to the content of the manuscript?
- Have you received reimbursements, fees, funding, or salary from an organization that holds or has applied for patents relating to the content of the manuscript?
- Do you have any other financial competing interests?
- Do you have any non-financial competing interests in relation to this paper?

If you can answer no to all of the above, write 'I declare that I have no competing interests' below. If your reply is yes to any, please give details below.

I declare that I have no competing interests

I agree to the open peer review policy of the journal. I understand that my name will be included on my report to the authors and, if the manuscript is accepted for publication, my named report including any attachments I upload will be posted on the website along with the authors' responses. I agree for my report to be made available under an Open Access Creative Commons CC-BY license (http://creativecommons.org/licenses/by/4.0/). I understand that any comments which I do not wish to be included in my named report can be included as confidential comments to the editors, which will not be published.

I agree to the open peer review policy of the journal

To further support our reviewers, we have joined with Publons, where you can gain additional credit to further highlight your hard work (see: https://publons.com/journal/530/gigascience). On publication of this paper, your review will be automatically added to Publons, you can then choose whether or not to claim your Publons credit. I understand this statement.

Yes
