# Supplementary material for: Comprehensive analysis of microorganisms accompanying human archaeological remains
Source: Gigascience. 2017 Jun 13;6(7):1–13. doi: 10.1093/gigascience/gix044 (PMC5965364; doi:10.1093/gigascience/gix044)
Supplement: Reviewer_1_Report_(Revision_1).pdf [file gix044_reviewer_1_report_(revision_1).pdf]

Reviewer Report

Title: Comprehensive analysis of microorganisms accompanying human archaeological remains

Version: Revision 1 **Date:** 5/17/2017

Reviewer name: Pablo Librado

Reviewer Comments to Author:

I highly appreciate authors considered all my comments, and think this paper is should be now suitable for publication. I have a few minor comments that the authors can address without further revisions, under the supervision/decision of the editor.

- line 84, I would change: "until now were not comparatively surveyed" by "have not been exhaustively compared so far"
- line 88, parenthesis not needed in ([41-44]). Same in line 352
- Number of decimals is inconsistent. Sometimes 4, and sometimes 5 floating digits.
- line 128, I would replace "the minor statistically significant difference" by "Marginal statistical significance was"
- the authors claim "we showed that the composition of microbiome of archeological remains does not depend on the way and time of storage". I do not think this conclusion is necessarily true, given their tests and many other publications contradicting it. What authors found is that the variance amongst samples is so high that it obscures any trend created by the way and time of storage. In other words, that sample peculiarities are more important for microbial composition than way and time of storage. It does not mean that way or time of storage are not important. Please, check and rephrase across the whole manuscript.

And finally what I find more controversial is still the massive virus content. I am not going to ask for the same once again, but in the previous round of reviews, I asked for some kind of damage profiling of virus reads, because I am a bit puzzled about the proportion of viruses in some samples. Although viruses are discarded in many aDNA metagenomic analyses, the fact that some samples show >60% is really shocking. Does it mean that most of the sequencing effort is going for virus sequencing? Given the importance that this could have in aDNA, I do not think the authors can just briefly discuss it, and disregard the virus fraction for the rest of the paper. I felt and I still feel that a more accurate analysis/interpretation is compulsory.

The authors replied they cannot address my suggestion because "unfortunately at present it is rather impossible because their genomes are composed of RNA". I am not a lab expert, but ... how can you sequence RNA viruses using DNA sequencing protocols? Are they retroviruses? Are they integrated in the host genomes? Based on table S1, it seems to be a correlation between virus and endogenous content, but it could be indirectly explained by the fraction of prokaryotes (i.e. more bacteria, less endogenous and less viruses). I have discussed with two colleagues, experts in aDNA metagenomics, and

they are equally confused. They even suggested contamination, as apparently you did not sequence the blanks, and the 100-fold DNA enrichment between extracts and blanks is not much for aDNA standards. But we all agree that 1% of contamination cannot drive the >60% of virus found in some aDNA remains, unless there are sequencing artifacts as recently shown for Illumina HiSeq 4000. I am not saying the paper should be rejected. On the contrary, as it is mainly descriptive paper, I think it is more than ok to describe potential caveats in aDNA metagenomics. But I would strongly recommend to strength their discussion/interpretation for this particular point, as it looks crucial, at least to avoid suspicious interpretations (as my colleagues suggested).

Methods

Are the methods appropriate to the aims of the study, are they well described, and are necessary controls included? Yes

Conclusions

Are the conclusions adequately supported by the data shown? Yes

Reporting Standards

Does the manuscript adhere to the journal's guidelines on minimum standards of reporting? Yes Choose an item.

Statistics

Are you able to assess all statistics in the manuscript, including the appropriateness of statistical tests used? Yes, and I have assessed the statistics in my report.

Quality of Written English

Please indicate the quality of language in the manuscript: Acceptable

Declaration of Competing Interests

Please complete a declaration of competing interests, considering the following questions:

- Have you in the past five years received reimbursements, fees, funding, or salary from an organisation that may in any way gain or lose financially from the publication of this manuscript, either now or in the future?
- Do you hold any stocks or shares in an organisation that may in any way gain or lose financially from the publication of this manuscript, either now or in the future?
- Do you hold or are you currently applying for any patents relating to the content of the manuscript?

- Have you received reimbursements, fees, funding, or salary from an organization that holds or has applied for patents relating to the content of the manuscript?
- Do you have any other financial competing interests?
- Do you have any non-financial competing interests in relation to this paper?

If you can answer no to all of the above, write 'I declare that I have no competing interests' below. If your reply is yes to any, please give details below.

I declare that I have no competing interests

I agree to the open peer review policy of the journal. I understand that my name will be included on my report to the authors and, if the manuscript is accepted for publication, my named report including any attachments I upload will be posted on the website along with the authors' responses. I agree for my report to be made available under an Open Access Creative Commons CC-BY license (http://creativecommons.org/licenses/by/4.0/). I understand that any comments which I do not wish to be included in my named report can be included as confidential comments to the editors, which will not be published.

I agree to the open peer review policy of the journal

To further support our reviewers, we have joined with Publons, where you can gain additional credit to further highlight your hard work (see: https://publons.com/journal/530/gigascience). On publication of this paper, your review will be automatically added to Publons, you can then choose whether or not to claim your Publons credit. I understand this statement.

Yes
