# Supplementary material for: Comprehensive analysis of microorganisms accompanying human archaeological remains
Source: Gigascience. 2017 Jun 13;6(7):1–13. doi: 10.1093/gigascience/gix044 (PMC5965364; doi:10.1093/gigascience/gix044)
Supplement: Reviewer_2_Report_(Original_Submission).pdf [file gix044_reviewer_2_report_(original_submission).pdf]

**Reviewer Report**

**Title:** Comprehensive analysis of microorganisms accompanying human archaeological remains

**Version:** Original Submission **Date:** 4/11/2017

**Reviewer name:** Alexander Herbig

**Reviewer Comments to Author:**

In their manuscript the authors describe a metagenomic assessment of archaeological remains with a focus on bacterial communities. The study targets multiple archaeological samples of varying age and storage conditions. The authors attempt to differentiate between bacterial species that might have been present before the individuals death and environmental contamination.
The methods employed by the authors are suitable for metagenomic analyses and the applied statistics are appropriate. However, considering the fact that DNA of human endogenous pathogens is usually only found in very low amounts in archaeological samples, the concept of MetaPhlAn might lack sensitivity. Only reads mapping to predefined marker regions are used and therefore most of the data is discarded. This limits the possibilities for the authentication of species identifications. The authors discuss these limitations also in the context of the fact that many human related bacteria have close relatives in the soil. I recommend to discuss this a bit more specifically for individual taxa. Some suggestions are given below.
Overall, this study provides a useful comparative resource for the assessment of bacterial communities that accompany human remains in an archaeological context.

In the abstract the authors claim that "comprehensive characterization of microbial DNA accompanying human remains has never been done."
In fact many studies do comprehensively characterize microbial DNA in the context of human remains in addition to focusing on a specific pathogenic species. I assume what the authors mean by 'comprehensive' is that this has not been done systematically for a wide range of different samples.
The authors should make this clearer also in the introduction.

The authors indicate that they found substantial amounts of Methanobrevibacter and describe it as "typically found in the human digestive system".
It is very probable that the species M. oralis was detected here, which is commonly found in the oral cavity.

Furthermore, the authors indicate that Bordetella was found as a taxon potentially representing a human pathogen. The authors mention in the discussion that B. bronchiseptica can also survive in the environment. In addition, it should be mentioned that this genus also contains species that are commonly found in the environment, such as B. petrii.
The same is true for other taxa that the authors call 'potential pathogens' such as Stenotrophomonas and Clostridium.

In particular for Clostridia it is rather unlikely that one of the individuals was infected with a bacterium of this genus. The reason that it is detected in the context of human remains is not surprising as it colonizes the human body after death.
In general the authors only distinguish between human-related and environmental taxa. Bacteria involved in body decomposition overlap with both groups in some way. Therefore, I think that these bacteria should be mentioned as a third component in addition to human endogenous bacteria and environmental taxa.

In the discussion the authors state "Our analyses revealed that some of them showed the DNA damage patterns closer to the environmental microbes than to the human associated ones."
This statement does not make sense to me. They might be more similar to environmental taxa on the sequence level, but I do not see what that has to do with damage patterns.
Furthermore, the authors state "This observation may indicate that DNA of some potential pathogens can be acquired from the environment after the organisms death."
I do not understand the intention of this statement. Environmental contamination is obvious and one of the subjects of the study. Why should environmental taxa that are similar to human pathogens be excluded from this? I suggest to omit this statement.

Also in the discussion it is stated: "We showed that identification of bacteria/archaea species accompanying the organism before death is possible using standard aDNA extraction protocols and shallow shotgun sequencing."
This is a very general statement. Other studies have shown that before to various extent.
Maybe the authors can be more specific here.
In addition, any bacterial taxon identified in this study should rather be seen as a candidate as the amount of data is not sufficient to authenticate the species identifications reliably.

**Methods**

Are the methods appropriate to the aims of the study, are they well described, and are necessary controls included? Yes

**Conclusions**

Are the conclusions adequately supported by the data shown? Yes

**Reporting Standards**

Does the manuscript adhere to the journal's guidelines on minimum standards of reporting? Yes Choose an item.

**Statistics**

Are you able to assess all statistics in the manuscript, including the appropriateness of statistical tests used? Yes, and I have assessed the statistics in my report.

**Quality of Written English**

Please indicate the quality of language in the manuscript: Acceptable

**Declaration of Competing Interests**

Please complete a declaration of competing interests, considering the following questions:

- Have you in the past five years received reimbursements, fees, funding, or salary from an organisation that may in any way gain or lose financially from the publication of this manuscript, either now or in the future?
- Do you hold any stocks or shares in an organisation that may in any way gain or lose financially from the publication of this manuscript, either now or in the future?
- Do you hold or are you currently applying for any patents relating to the content of the manuscript?
- Have you received reimbursements, fees, funding, or salary from an organization that holds or has applied for patents relating to the content of the manuscript?
- Do you have any other financial competing interests?
- Do you have any non-financial competing interests in relation to this paper?

If you can answer no to all of the above, write 'I declare that I have no competing interests' below. If your reply is yes to any, please give details below.

I declare that I have no competing interests

I agree to the open peer review policy of the journal. I understand that my name will be included on my report to the authors and, if the manuscript is accepted for publication, my named report including any attachments I upload will be posted on the website along with the authors' responses. I agree for my report to be made available under an Open Access Creative Commons CC-BY license (http://creativecommons.org/licenses/by/4.0/). I understand that any comments which I do not wish to be included in my named report can be included as confidential comments to the editors, which will not be published.

I agree to the open peer review policy of the journal

To further support our reviewers, we have joined with Publons, where you can gain additional credit to further highlight your hard work (see: https://publons.com/journal/530/gigascience). On publication of this paper, your review will be automatically added to Publons, you can then choose whether or not to claim your Publons credit. I understand this statement.

Yes
